# Genetically predicted dietary intake and risks of colorectal cancer: a Mendelian randomisation study

**DOI:** 10.1186/s12885-024-12923-1

**Published:** 2024-09-17

**Authors:** Tung Hoang, Sooyoung Cho, Ji-Yeob Choi, Daehee Kang, Aesun Shin

**Affiliations:** 1 Department of Preventive Medicine, Seoul National University College of Medicine, Seoul, 03080 South Korea; 2 Integrated Major in Innovative Medical Science, Seoul National University Graduate School, Seoul, Korea; 3 University of Health Sciences, Vietnam National University Ho Chi Minh City, Binh Duong, Vietnam; 4 Medical Research Center, Genomic Medicine Institute, Seoul National University College of Medicine, Seoul, Korea; 5 Department of Biomedical Science, Seoul National University Graduate School, Seoul, Korea; 6 BK4 Smart Healthcare, Seoul National University College of Medicine, Seoul, Korea; 7 Institute of Health Policy and Management, Seoul National University Medical Research Center, Seoul, Korea; 8 Cancer Research Institute, Seoul National University, Seoul, Korea

**Keywords:** Diet, Colorectal cancer, Genome-wide association study, Mendelian randomisation

## Abstract

**Background:**

Effects of confounders on associations between diet and colorectal cancer (CRC) in observational studies can be minimized in Mendelian randomization (MR) approach. This study aimed to investigate observational and genetically predicted associations between dietary intake and CRC using one-sample MR.

**Methods:**

Using genetic data of over 93 million variants, we performed a genome-wide association study to find genomic risk loci associated with dietary intake in participants from the UK Biobank. Then we calculated genetic risk scores of diet-related variants and used them as instrumental variables in the two-stage least square MR framework to estimate the hazard ratios (HRs) and 95% confidence intervals (CIs) for associations. We also performed observational analyses using age as a time-scale in Cox proportional hazard models.

**Results:**

Allele scores were calculated from 399 genetic variants associated with the consumption of of red meat, processed meat, poultry, fish, milk, cheese, fruits, vegetables, coffee, tea, and alcohol in participants from the UK Biobank. In MR analysis, genetically predicted fruit intake was significantly associated with a 21% decreased risk of CRC (HR = 0.79, 95% CI = 0.66–0.95), and there was a marginally inverse association between vegetable intake and CRC (HR = 0.85, 95% CI = 0.71–1.02). However, null findings were observed in multivariable analysis, with HRs (95% CIs) of 0.99 (0.98–1.01) and 0.99 (0.98–1.00) per increment of daily servings of fruits and vegetables, respectively.

**Conclusion:**

Dietary habits were attributable to genetic variations, which can be used as instrumental variables in the MR framework. Our study supported a causal relationship between fruit intake and a decreased risk of CRC and suggested an effective strategy of consuming fruits in the primary prevention of CRC.

**Supplementary Information:**

The online version contains supplementary material available at 10.1186/s12885-024-12923-1.

## Introduction

With a global burden of 1.9 million new cases and 0.9 million deaths estimated in 2020, colorectal cancer (CRC) is the third most common cancer type and the second most common cancer death due to this malignancy in the world [1]. Regarding the prevention of CRC, the World Cancer Research Fund/American Institute for Cancer Research (WCRF/AICR) launched the guidance every 10 years based on up-to-date systematic reviews and meta-analyses and reported the level of evidence for the association of different dietary factors with CRC risk [2]. Observational studies may be vulnerable to residual confounding by factors that cannot be measured, and this may limit it in interpreting such an observed association as a causal relationship [3, 4]. In the meantime, by examining genetic variants such as single nucleotide polymorphisms (SNPs) as instrument variables (IVs) that act as proxies for environmental factors, Mendelian randomisation (MR) was suggested to provide a useful approach to minimise the bias of the effect estimate between risk factors and CRC risk [5–7].

A previous MR study comprehensively examined the causal inference of modifiable factors with the CRC risk [8]. Among 39 risk factors, only coffee consumption was included in the analysis due to unavailable or unsuitable SNPs for the use as instrumental variables (IVs) for other dietary factors [8]. Given a substantial proportion of the preference for foods was explained by genetic variations, individual food preferences and dietary habits have been identified to be affected by the senses of taste and smell and metabolic processes [9–11]. Additionally, a previous comprehensive genome-wide association study (GWAS) reported hundreds of significant loci for single foods and dietary patterns in participants of the UK Biobank [12]. However, underlying biological mechanisms contributing to genetic variations for the intake of several food items (e.g., pork vs. beef vs. lamb/mutton, oily vs. nonoily fish, fresh vs. dried fruits, cooked vs. raw vegetables) have been still unclear. Therefore, we first carried out a GWAS of food intake to identify genetic variants associated with the intake of total red meat, processed meat, poultry, total fish, milk, cheese, total fruits, total vegetables, coffee, tea, and alcohol, using updated data of more than double number of SNPs compared to the previous study. We then performed a one-sample MR study to elucidate the association between genetically predicted dietary intake and CRC risk using GWAS-identified genomic risk loci as IVs.

## Materials and Methods

### Study population

The UK Biobank is a prospective cohort study that included 502,389 participants aged 37–73 years who resided within 25 miles of 22 recruiting centers between 2006 and 2010. The study was approved by the North West Multi-centre Research Ethics Committee. The methodological details and rationale of the UK Biobank have been published elsewhere [13–15].

**Fig. 1:**
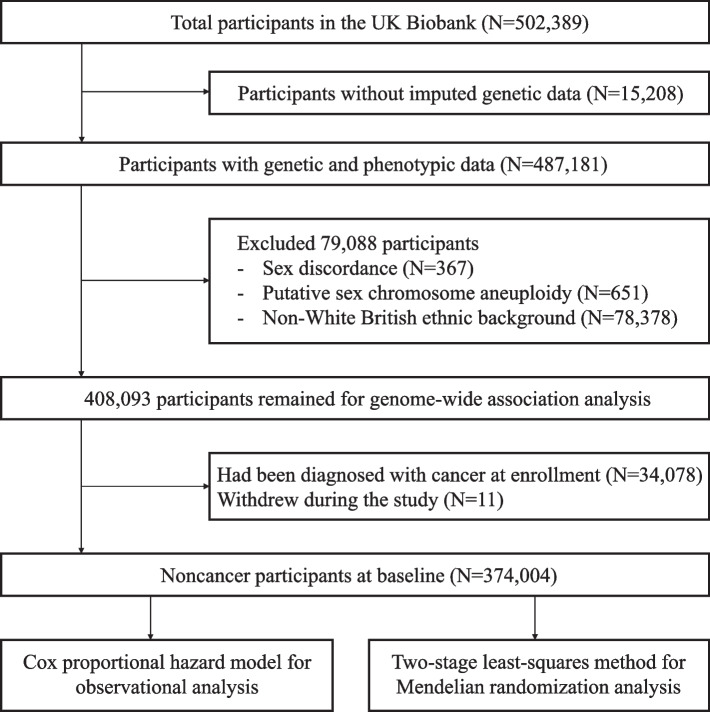
Flow diagrams of study participants and analytical framework

In the present study, we mutually excluded participants without genetic information (*N* = 15,208), sex mismatch (*N* = 367), putative sex chromosome aneuploidy (*N* = 651), and those who were either genetically identified or self-reported as having ethnic backgrounds other than White British (including White, British, Irish, and any other White backgrounds) (*N* = 78,378). After exclusion, the sample available for the genome-wide association analysis was restricted to 408,093 individuals. Finally, we excluded participants who were diagnosed with any cancers at enrolment (*N* = 34,078) and those who withdrew from the study during the follow-up (*N* = 11), leaving a total of 374,001 individuals (Fig. 1).

### Genotyping and quality control

Genotyping was performed using either the custom UK Biobank Axiom Array or the Affymetrix Axiom Array, as described elsewhere [14, 15]. Genotyping data were imputed using both the UK10K and 1000 Genomes Phase 3 and the Haplotype Reference Consortium reference panel, which resulted in a total of 93,095,623 markers [14]. Following the quality control procedure, we excluded SNPs with low imputation quality (imputed score < 0.3, *n* = 15,368,777), high missingness (geno > 0.05, *n* = 909,502), low minor allele frequency (maf < 0.0002, *n* = 55,398,429) and those that deviated from the expected Hardy–Weinberg equilibrium (*p* < 1e-6, *n* = 8,717,604) [16]. A total of 27,503,596 SNPs that passed the quality filtering remained.

### Dietary intake assessment

A touchscreen food frequency questionnaire (FFQ) was used to assess food and beverage intake in the preceding year [17]. Details of the questionnaire were publicly available [18]. In this study, we included foods that were documented in the WCRF report for their associations with CRC risk at various levels of evidence. We also selected foods for which consumption could reasonably be attributed to genetic variations (Additional file 1: eInformation). A linear mixed model was applied to adjust for familial relatedness in genome-wide association analysis of food intake; thus, we converted dietary outcomes into quantitative traits (Table 1). Of these, frequency traits of beef, pork, lamb, processed meat, poultry, oily fish, nonoily fish, cheese, and alcohol intake, and quantitative traits of fresh and dried fruits, cooked and raw vegetables, and coffee and tea consumption were included in our analyses. For categorical phenotypes, we used the corresponding numeric values (times/week) for the analysis. To justify the selection of dietary factors, we combined food items into more common food groups which are similar to those from the WCRF report. We grouped single items to obtain the total intake of red meat (including pork, beef, and lamb), total fish (including oily and nonoily fish), total fruits (including fresh and dried fruits), and total vegetables (including cooked and raw vegetables) [19]. Milk consumption (mL/day) was estimated based on the type of milk, breakfast cereal, coffee, and tea intake [19]. The 24-h dietary data were used to validate the estimation of milk intake, and 94% of the total milk consumption was found to come from milk added to breakfast cereal, coffee, and tea [19]. Overall, the Shapiro–Wilk test was applied to assess the normality of the data, and for data not following a normal distribution, the median and interquartile range was reported for data that did not follow normal distribution.

**Table 1:** Summary of the process converting dietary items from the food frequency questionnaire into quantitative traits

Food item	Response and conversion	Food group
Pork	Never: 0 Less than once a week: 0.5 time/week Once a week: 1 time/week 2–4 times a week: 3 times/week 5–6 times a week: 5.5 times/week Once or more daily: 7 times/week	Red meat (times/week) = pork + beef + lamb
Beef		
Lamb		
Processed meat		-
Poultry		-
Oily fish		Total fish (times/week) = oily fish + non-oily fish
Non-oily fish		
Cheese		-
Milk type	Never/ rarely having, full cream, semi-skimmed, skimmed milk Milk (mL/day) = 0 if never/ rarely having milk Milk (mL/day) = 100 x bowls of breakfast cereals + 25 x cups of coffee + 35 x cups of tea	-
Fresh fruits	Pieces/day	Total fruits (servings/day) = fresh fruits + ½ x dried fruits
Dried fruits	Pieces/day	
Cooked vegetables	Tablespoons/day	Total vegetables (servings/day) = cooked vegetables + raw vegetables
Raw vegetables	Tablespoons/day	
Coffee	Cups/day	-
Tea	Cups/day	-
Alcohol	Never: 0 Special occasions only: 0.125 time/week One to three times a month: 0.5 time/week One to two times a week: 1.5 times/week Three to four times a week: 3.5 times/week Five to six times a week: 5.5 times/week Daily or almost daily: 7 times/week	-

### Outcome ascertainment

Incident CRC cases were determined via the ICD-10 code, in which CRC was defined as either colon cancer (C18.0-C18.9) or rectal cancer (C19 and C20). Time to follow-up was defined as the date of study enrolment until the date of CRC diagnosis, death, lost-to-follow-up, or end of follow-up (June 25, 2021), whichever came first.

### Instrumental variables for dietary phenotype

To identify genetic variants associated with dietary traits, we performed a GWAS for food intake (Additional file 1: eMethod). In brief, we performed a genome-wide association analysis under the linear mixed model approach [20]. We incorporated age, sex, and the first 6 first principal component scores released by the UK Biobank [14] as covariates. In the large-scale UK Biobank dataset, more than 30% of study participants were genetically defined to relate with another participant [14]. Therefore, we further adjusted for the cryptic relatedness among participants by calculating the sparse genetic relatedness matrix (GRM) using genotyping data of 93,183 SNPs, which were used for the final kinship inference of the released UK Biobank data [14, 21, 22]. The list of genomic risk loci and their functions were determined under the SNP2GENE and GENE2FUNC functions of the web-based FUMA tool [23].

In sensitivity analysis, we excluded genetic variants, which were associated with more than two dietary traits from the list of IVs for dietary intake to minimise the possibility of horizontal pleiotropy. Additionally, for the exclusion restriction assumption, we further excluded SNPs that were associated with CRC risks (*p*-value < 0.05) from the list of candidate IVs to minimise the possibility of genetic variants affecting CRC other than through dietary intake. Details on the estimation of beta coefficients for the effect of variants on CRC risks adjusting for familial relatedness were available at Additional file 1: eMethod.

The internally weighted allele score for each participant was calculated by multiplying the number of effect alleles that the participant carried by the corresponding beta-coefficient of the association between the genetic variant and dietary intake estimated from the genome-wide association. Then we summed up the weighted allele score of individual genetic variants and used them as IVs in the MR analysis.

To assess the weak instrument problem, an *F*-statistic was implemented for IVs of allele scores and their corresponding individual genetic variants [24]. *F*-statistic was approximated by a squared estimate for IVs on dietary intake frequency divided by its variance.

### Mendelian randomisation analysis

We carried out a one-sample MR in the UKB to assess the effect of dietary intake on CRC using the two-stage least square method [25, 26]. In the first stage, we regressed each food frequency consumption on its respective allele score using a linear regression model to obtain a set of fitted values for exposure of interest. In the second stage, we regressed the CRC outcome on the fitted values obtained in stage 1 using an age-scale Cox proportional hazard model. Additionally, we used the MR pleiotropy residual sum and outlier test (MR-PRESSO) to detect the presence of pleiotropy [27] and the MR-Egger regression to identify whether directional pleiotropy may influence the causal estimates [28]. Subgroup analyses were conducted by sex and CRC subsites.

In sensitivity analysis, we carried out a multivariable MR, which included multiple dietary factors which their allele scores were substantially correlated or had relatively high genetic correlations.

### Observational association

We sought to evaluate the association between dietary intake (in a continuous form) and CRC risk using age as a time-scale in Cox proportional hazard models. In the multivariable analysis, we adjusted for confounders, including sex, family history of CRC, household income, smoking, alcohol consumption (except for alcohol consumption exposure), body mass index, and physical activity, which were associated with CRC risk in the univariate analysis.

## Results

### Study population characteristics

Table 2 summarises the general characteristics and dietary habits of 174,576 men and 199,428 women without any cancers at enrolment. At recruitment, participants were aged 56.6 years (mean ages 56.5 years for men and 56.8 years for women). After a median follow-up of 12.4 years (interquartile range 11.6–13.1 years), 3,131 colon cancer and 1,555 rectal cancer cases were newly detected.

**Table 2:** Baseline characteristics and dietary habits of study participants in the UK Biobank

Factor	Total (*N* = 374,004)	Men (*N* = 174,576)	Women (*N* = 199,428)
Age at recruitment (years), mean ± standard deviation	56.6 ± 8.0	56.5 ± 7.9	56.8 ± 8.1
Follow-up time (years), median (Q1-Q3)	12.36 (11.63–13.05)	12.32 (11.57–13.03)	12.38 (11.68–13.07)
Incident colorectal cancer, N (%)	4,686 (1.3%)	1,979 (1.0%)	2,707 (1.6%)
Incident colon cancer, N (%)	3,131 (0.8%)	1,437 (0.7%)	1,694 (1.0%)
Incident rectal cancer, N (%)	1,555 (0.4%)	542 (0.3%)	1,013 (0.6%)
Red meat			
- Frequency (times/week), median (Q1-Q3)	2 (1.5–2.5)	2 (1.5–2.5)	1.5 (1.5–2.5)
- Missing, N (%)	511 (0.14%)	273 (0.16%)	238 (0.12%)
Processed meat			
- Frequency (times/week), median (Q1-Q3)	1 (0.5–3)	1 (1–3)	1 (0.5–1)
- Missing, N (%)	529 (0.14%)	261 (0.15%)	268 (0.13%)
Poultry			
- Frequency (times/week), median (Q1-Q3)	1 (1–3)	1 (1–3)	1 (1–3)
- Missing, N (%)	595 (0.16%)	334 (0.19%)	261 (0.13%)
Total fish			
- Frequency (times/week), median (Q1-Q3)	2 (1–3.5)	1.5 (1–3.5)	2 (1.5–3.5)
- Missing, N (%)	444 (0.12%)	254 (0.15%)	190 (0.10%)
Milk			
- Frequency (100 mL/day), median (Q1-Q3)	2.3 (1.71–2.91)	2.35 (1.75–2.96)	2.3 (1.7–2.9)
- Missing, N (%)	19,103 (5.11%)	6,567 (3.76%)	12,536 (6.29%)
Cheese			
- Frequency (times/week), median (Q1-Q3)	3 (1–3)	3 (1–3)	3 (1–3)
- Missing, N (%)	8,522 (2.79%)	3,841 (2.20%)	4,681 (2.35%)
Total fruits			
- Frequency (servings/day), median (Q1-Q3)	2.25 (1–3.5)	2 (1–3)	2.5 (1.5–3.5)
- Missing, N (%)	559 (0.15%)	373 (0.21%)	186 (0.09%)
Total vegetables			
- Frequency (servings/day), median (Q1-Q3)	4 (3–6)	4 (3–6)	4 (3–6)
- Missing, N (%)	2,131 (0.57%)	1,454 (0.83%)	677 (0.34%)
Coffee			
- Frequency (cups/day), median (Q1-Q3)	2 (0.5–3)	2 (0.5–3)	1 (0.5–3)
- Missing, N (%)	608 (0.16%)	325 (0.19%)	283 (0.14%)
Tea			
- Frequency (cups/day), median (Q1-Q3)	3 (1–5)	3 (1–5)	3 (1–5)
- Missing, N (%)	703 (0.19%)	325 (0.19%)	378 (0.19%)
Alcohol			
- Frequency (times/day), median (Q1-Q3)	1.5 (0.5–3.5)	3.5 (1.5–7)	1.5 (0.5–3.5)
- Missing, N (%)	261 (0.07%)	132 (0.08%)	129 (0.06%)

### Loci and annotation of SNPs related to dietary intake

**Fig. 2:**
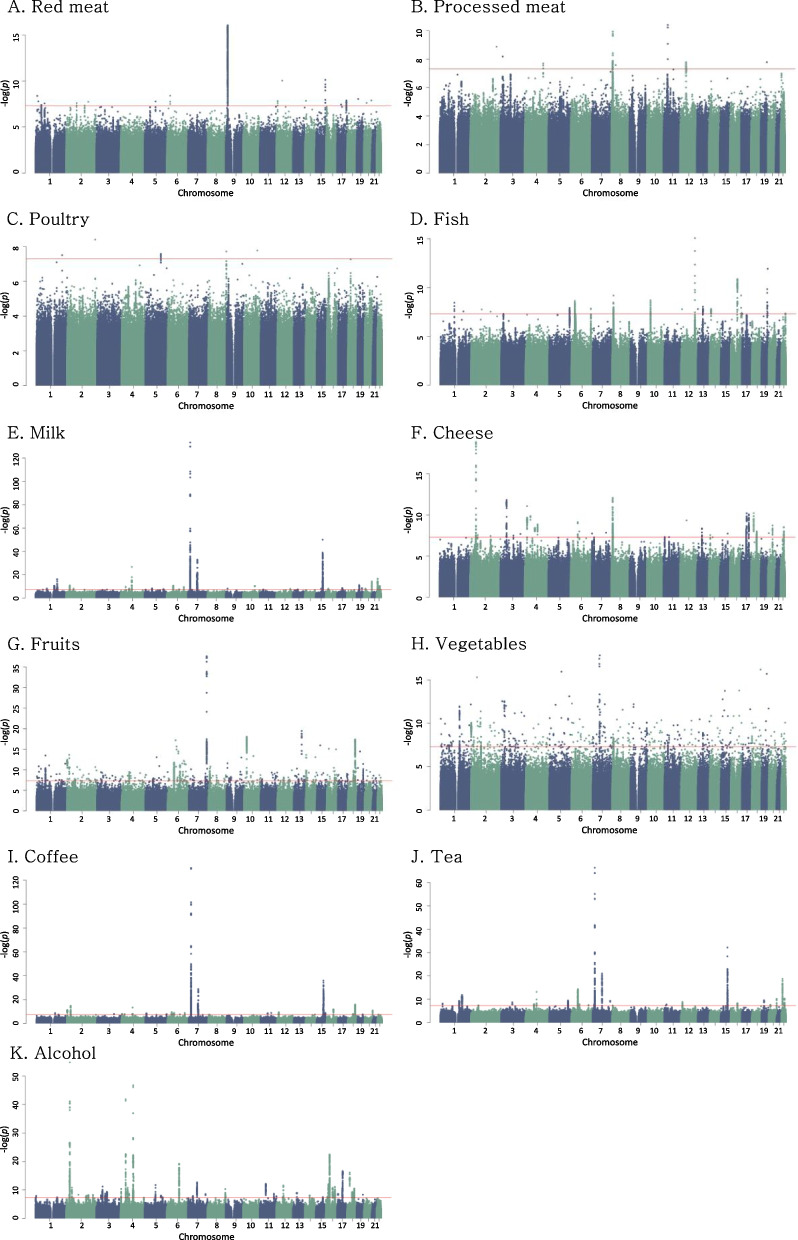
Manhattan plot of genome-wide association analyses of **A** red meat, **B** processed meat, **C** poultry, **D** fish, **E** milk, **F** cheese, **G** fruit, **H** vegetable, **I** coffee, **J** tea, and **K** alcohol consumption using linear mixed model. X-axis shows chromosome positions, Y-axis shows -log10 of *p*-values. Red dashed lines indicate significant threshold (*p* = 5e-8)

The results from the genome-wide association analysis for significant SNPs (*p* < 5 × 10^–8^) associated with food intake are presented as Manhattan plots (Fig. 2). We identified a total of 402 genomic risk loci for the consumption of red meat (*n* = 15), processed meat (*n* = 12), poultry (*n* = 1), total fish (*n* = 28), milk (*n* = 50), cheese (*n* = 59), total fruits (*n* = 82), total vegetables (*n* = 50), coffee (*n* = 33), tea (*n* = 40), and alcohol (*n* = 57) in the linear mixed model adjusting for familial relatedness (Additional file 2: Table S1). Of these, variants rs2199936 (chromosome 4, *ABCG2* gene), rs139797380 (chromosome 6, *SLC35D3* gene), and rs4410790 (chromosome 7, *AC003075.4* gene) were associated with milk, coffee, and tea consumption. Variant 2:27,748,992 (chromosome 2, *GCKR* gene) was associated with the consumption of milk, coffee, and alcohol. Variant rs8103840 (chromosome 19, *FUT1* gene) was associated with the intake of processed meat, fish, and fruits. In addition, some SNPs were associated with two dietary factors, including rs201406724 (milk and tea), rs11940694 (milk and alcohol), rs2465018 (milk and tea), rs17685 (milk and tea), rs4726481 (tea and alcohol), rs7012814 (cheese and tea), 8:73,433,232 (milk and tea), rs11032362 (processed meat and fruits), 12:11,271,915 (coffee and tea), rs12591786 (milk and tea), rs12909335 (milk and tea), rs9937521 (tea and alcohol), rs12459249 (milk and coffee), and rs429358 (fish and fruits).

Biological processes, molecular functions, and Wikipathways that may involve in insights into genetic effects on the intake of fish, milk, cheese, fruits, coffee, tea, and alcohol are presented in Additional file 2: Figures S1-S7. Overall, the heritability was highest for the consumption of cheese (h^2^ = 10.48%), alcohol (h^2^ = 9.71%), and milk (h^2^ = 9.01%), followed by tea (h^2^ = 8.34%) and fruits (h^2^ = 7.83%). Other foods had a heritability of approximately 5%-6%, except poultry (h^2^ = 3.50%) (Additional file 2: Table S2). Furthermore, we found a relatively high genetic relationship for the intake between milk and tea (r = 0.86), fish and vegetables (r = 0.52), fruits and vegetables (r = 0.49), red meat and processed meat (r = 0.48), processed meat and fruits (r = -0.46), cheese and alcohol (r = 0.44), and red meat and poultry (r = 0.43) (Additional file 2: Figure S8). The highest Pearson correlation coefficients between food consumption were found for coffee and tea (r = -0.32) and milk and tea (r = 0.30) (Additional file 2: Figure S8).

### Mendelian randomisation analysis of dietary intake and colorectal *cancer* risk

All genetic instruments of SNPs and allele scores predicted dietary intake frequency, with *F*-statistics greater than 10, are presented in Tables 3, Additional file 2: Tables S1 and S3. Since only one variant was associated with poultry intake, we did not calculate the MR estimate for the effect of poultry intake on CRC.

**Table 3:** Summary of all eligible instrumental variables used in this study

Dietary factor	No. SNPs	F-statistics		
		**Total**	**Men**	**Women**
Red meat	15	477	280	206
Processed meat	12	357	215	142
Total fish	28	889	387	506
Milk	50	2,964	1,337	1,634
Cheese	59	2,076	929	1,170
Total fruits	81	2,867	1,679	1,274
Total vegetables	50	1,398	389	1,097
Coffee	33	2,210	1,014	1,201
Tea	40	1,987	848	1,145
Alcohol	57	2,297	1,135	1,302

*SNP* Single nucleotide polymorphism

Table 4 shows the estimates of the causal effect of dietary intake on CRC risks in the one-sample MR approach using the full lists of genetic variants. Overall, genetically proxied fruit intake was associated with 21% decreased risks of both CRC (HR = 0.79, 95% CI = 0.66–0.95) and colon cancer (HR = 0.79, 95% CI = 0.63–0.99). Findings for other dietary factors were not significant: red meat (HR = 0.72, 95% CI = 0.40–1.28), processed meat (HR = 0.57, 95% CI = 0.29–1.11), fish (HR = 1.05, 95% CI = 0.72–1.53), milk (HR = 1.19, 95% CI = 0.86–1.63), cheese (HR = 0.98, 95% CI = 0.78–1.23), coffee (HR = 1.16, 95% CI = 0.96–1.40), tea (HR = 0.95, 95% CI = 0.82–1.11), and alcohol (HR = 1.01, 95% CI = 0.86–1.20). These associations remained after excluding genetic variants associated with more than one dietary phenotype or related to CRC risks (Additional file 2: Table S4). In sex-specific subgroups, CRC reduction was only observed in women for an increment of 1 serving/day of consuming fruits in both the main analysis of including all eligible variants and the sensitivity analysis of the reduced list of variants, with HRs (95% CIs) of 0.72 (0.53–0.98) and 0.69 (0.50–0.96), respectively. Furthermore, genetically proxied alcohol consumption was associated with a 22% increased risk of CRC in men. However, this association disappeared in the sensitivity analysis using the reduced list of variants.

**Table 4:** Mendelian randomisation estimates for associations of genetically dietary intake with colorectal cancer risk using full list of variants

Dietary factor	Outcome	Total		Men		Women	
		**HR (95% CI)**	**P**_**pleiotropy**_	**HR (95% CI)**	**P**_**pleiotropy**_	**HR (95% CI)**	**P**_**pleiotropy**_
Red meat	Colorectal cancer	0.72 (0.40–1.28)	0.14	0.78 (0.41–1.50)	0.12	0.63 (0.22–1.78)	0.31
	Colon cancer	0.93 (0.46–1.88)	0.34	1.06 (0.47–2.40)	0.12	0.76 (0.23–2.56)	0.49
	Rectal cancer	0.42 (0.16–1.15)	0.45	0.47 (0.16–1.37)	0.73	0.38 (0.05–2.77)	0.58
Processed meat	Colorectal cancer	0.57 (0.29–1.11)	0.22	0.59 (0.29–1.21)	0.19	0.50 (0.12–2.07)	0.94
	Colon cancer	0.79 (0.35–1.78)	0.68	0.79 (0.32–1.93)	0.72	0.76 (0.14–4.03)	0.85
	Rectal cancer	**0.29 (0.09–0.93)**	0.84	0.37 (0.12–1.18)	**0.01**	0.16 (0.01–2.45)	0.63
Total fish	Colorectal cancer	1.05 (0.72–1.53)	0.11	1.20 (0.72–2.01)	0.18	0.86 (0.49–1.52)	0.56
	Colon cancer	1.06 (0.67–1.68)	0.24	1.22 (0.64–2.32)	0.34	0.89 (0.46–1.73)	0.59
	Rectal cancer	1.03 (0.54–1.99)	0.60	1.18 (0.51–2.73)	0.79	0.78 (0.27–2.31)	0.27
Milk	Colorectal cancer	1.19 (0.86–1.63)	0.69	1.13 (0.74–1.73)	0.07	1.26 (0.77–2.06)	0.92
	Colon cancer	1.21 (0.82–1.79)	0.22	1.12 (0.66–1.91)	0.24	1.32 (0.74–2.34)	0.87
	Rectal cancer	1.14 (0.66–1.99)	0.69	1.16 (0.58–2.30)	0.24	1.10 (0.43–2.87)	0.50
Cheese	Colorectal cancer	0.98 (0.78–1.23)	0.24	0.93 (0.69–1.26)	0.45	1.07 (0.76–1.50)	0.81
	Colon cancer	1.02 (0.78–1.34)	0.09	1.03 (0.71–1.51)	0.16	1.01 (0.68–1.50)	0.56
	Rectal cancer	0.91 (0.62–1.34)	0.59	0.78 (0.48–1.27)	0.46	1.24 (0.65–2.35)	0.53
Total fruits	Colorectal cancer	**0.79 (0.66–0.95)**	0.90	0.85 (0.68–1.05)	0.81	**0.72 (0.53–0.98)**	0.99
	Colon cancer	**0.79 (0.63–0.99)**	0.77	0.84 (0.64–1.10)	0.63	0.73 (0.51–1.05)	0.98
	Rectal cancer	0.79 (0.58–1.09)	0.99	0.86 (0.60–1.21)	0.96	0.68 (0.38–1.24)	0.99
Total vegetables	Colorectal cancer	0.85 (0.71–1.02)	0.93	0.78 (0.58–1.06)	> 0.99	0.91 (0.73–1.15)	0.67
	Colon cancer	0.80 (0.64–1.01)	0.99	0.73 (0.49–1.09)	> 0.99	0.85 (0.64–1.13)	0.99
	Rectal cancer	0.95 (0.71–1.26)	0.98	0.86 (0.54–1.38)	> 0.99	1.05 (0.73–1.50)	0.99
Coffee	Colorectal cancer	1.16 (0.96–1.40)	0.51	1.18 (0.93–1.50)	0.62	1.14 (0.84–1.53)	0.79
	Colon cancer	1.24 (0.99–1.56)	0.09	1.16 (0.85–1.56)	0.23	1.36 (0.95–1.93)	0.55
	Rectal cancer	1.02 (0.74–1.42)	0.58	1.22 (0.82–1.80)	0.66	0.71 (0.40–1.26)	**0.02**
Tea	Colorectal cancer	0.95 (0.82–1.11)	0.98	0.90 (0.73–1.11)	0.56	1.03 (0.81–1.30)	0.65
	Colon cancer	0.87 (0.72–1.05)	0.57	0.79 (0.61–1.03)	0.41	0.97 (0.74–1.28)	0.41
	Rectal cancer	1.14 (0.87–1.50)	0.19	1.11 (0.79–1.57)	0.73	1.19 (0.76–1.86)	0.36
Alcohol	Colorectal cancer	1.11 (0.96–1.29)	0.28	**1.22 (1.01–1.47)**	0.82	1.00 (0.79–1.26)	0.23
	Colon cancer	1.11 (0.93–1.34)	0.06	1.17 (0.93–1.49)	0.10	1.06 (0.81–1.38)	0.62
	Rectal cancer	1.11 (0.86–1.44)	0.76	1.29 (0.95–1.76)	0.82	0.86 (0.56–1.34)	0.14

*HR* Hazard ratio, *CI* Confidence interval

*P*-values for pleiotropy effects are obtained from global test in Mendelian Randomization Pleiotropy RESidual Sum and Outlier (MR-PRESSO). Bold font indicates significant difference

Marginally inverse associations were found for vegetable intake and CRC (HR = 0.85, 95% CI = 0.71–1.02) and colon cancer (HR = 0.80, 95% CI = 0.64–1.01) risks. Using genetic variants associated with a single dietary phenotype and not related to CRC, the magnitude of associations was similar to that of all eligible variants, with HRs (95% CIs) of 0.84 (0.70–1.01) and 0.80 (0.63–1.01) for CRC and colon cancer, respectively.

In the sensitivity analysis of using multivariable MR with the inclusion of multiple dietary factors which their allele scores were substantially correlated (r > 0.10, Additional file 2: Figure S9A) or had relatively high genetic correlations (r > 0.30, Additional file 2: Figure S9B), the sets of red meat and processed meat; fish, total fruit, and total vegetables; milk, tea, and coffee; and cheese and alcohol were considered in the model. Accordingly, genetically predicted consumption of red meat, processed meat, and cheese was associated with an increased risk of CRC, with HRs (95% CIs) of 1.30 (1.19–1.43), 1.29 (1.18–1.41), and 1.36 (1.21–1.53), respectively (Additional file 2: Table S9). Furthermore, inverse associations were observed for associations between genetically predicted vegetable (HR = 0.94, 95% CI = 0.90–0.98) and tea (HR = 0.97, 95% CI = 0.95–0.99) consumption (Additional file 2: Table S9).

### Evaluation of pleiotropy effects

Although MR-PRESSO global tests suggested a possible bias from horizontal pleiotropy in associations of processed meat intake in men and coffee consumption in women with rectal cancer (Table 3, p_pleiotropy_ = 0.01), the estimates after correcting for outliers remained in similar directions of associations, with HRs (95% CIs) of 0.30 (0.03–3.21) and 0.72 (0.35–1.47), respectively. The MR-PRESSO distortion test showed that the distortion in the effect estimates before and after removing outliers was not significant. These possible pleiotropy effects disappeared in our sensitivity analysis of restricting genetic variants for IVs (Additional file 2: Table S4).

### Observational association

Additional file 2: Table S5 shows the observational effect of dietary intake on the risk of CRC. Red meat (HR = 1.05, 95% CI = 1.03–1.07, per 1 time/week), processed meat (HR = 1.03, 95% CI = 1.01–1.05, per 1 time/week), and alcohol consumption (HR = 1.03, 95% CI = 1.01–1.04, per 1 time/week) were positively associated with CRC risks. In contrast, more frequently milk (HR = 0.95, 95% CI = 0.92–0.97, per 100 mL/day) and tea (HR = 0.98, 95% CI = 0.97–0.99, per 1 cup/day) consumers had decreased risk of CRC. However, null associations were observed in multivariable analysis, with HRs (95% CIs) of 0.99 (0.98–1.01) and 0.99 (0.98–1.00) per increment of daily servings of fruits and vegetables, respectively. When stratified by sex, the effects of red meat, processed meat, and alcohol consumption remained for the men subgroup, whereas only the inverse association between milk intake and CRC risk was observed in women. Nevertheless, null findings were observed in multivariable analysis, with HRs (95% CIs) of 0.99 (0.98–1.01) and 0.99 (0.98–1.00) per increment of daily servings of fruits and vegetables, respectively.

In the analysis by CRC subsites, positive associations of red meat intake and inverse associations of milk and tea consumption were observed with both colon cancer and rectal cancer (Additional file 2: Tables S6-S7). Furthermore, processed meat (HR = 1.03, 95% CI = 1.01–1.05, per 1 time/day) and alcohol (HR = 1.03, 95% CI = 1.01–1.04, per 1 time/day) consumption showed an increased risk of colon cancer.

## Discussion

In this study, we identified 399 genomic risk loci for self-reported traits reflecting daily consumption of food items included in the WCRF report for CRC prevention (Additional file 2: Figure S10). Using these genomic risk loci in the one-sample MR framework, we found that genetically predicted dietary intake of fruits was associated with a lower risk of CRC, with a similar magnitude of an inverse association with colon cancer. Additionally, marginally inverse associations between vegetable intake with CRC and colon cancer were observed in the total study population. When compared with our observational analysis of a prospective cohort study design, these associations appeared to be weaker and did not reach the level of significance (Additional file 2: Figure S11).

When we searched PubMed up to September 2023 for the GWAS of dietary traits, a total of 23 GWAS were identified, and seven studies included the population of the UK Biobank (Additional file 2: Table S8). Our study extended to the previous research by accounted for familial relatedness, which was not adjusted in most previous GWAS. Besides, to justify the selection of dietary factors, we combined food items into more common food groups that underlying biological mechanisms contributing to genetic variations existed. In addition, we analysed updated data with more than double SNPs from the most comprehensive GWAS for dietary intake [12]. Moreover, we carried out functional analyses to inform possible biological mechanisms between genetic factors and food consumption. A detailed comparison of the identified variants and the heritability of genetic factors between our present GWAS and Cole’s study is further provided in Additional file 3: Appendix.

By obtaining dietary habits from the questionnaire, we considered the amount of food consumption in the continuous form and applied the linear mixed model. A previous study converted food-liking traits into numerical values (range 0–9) without justification [29]. Given the transformation of food preference phenotypes into the hedonic scale into numeric values is not appropriate, the proportional odds logistic mixed model (POLMM) has been shown to handle ordinal categorical phenotypes, especially when the phenotype is extremely imbalanced [30]. The authors applied the POLMM for the frequent consumption of food items (never or almost never, once every few months, once a month, once a week, 2–4 times per week, and almost daily) in the UK Biobank without converting into numeric values [30]. In our present study, modelling dietary intake frequencies as continuous variables may violate the assumption of linearity relationship between SNPs and food consumption due to the restriction of outcome variable ranges. Nevertheless, findings on the top 10 genes were similar to those identified from our current study (e.g., *CCDC171* for beef, pork, and lamb, *XKR6* for processed meat, *LY6H* for poultry, and *MLLT10* for oily fish).

The anti-cancer effects of fruits and vegetables were suggested due to their bioactive compounds, such as fiber, folate, vitamins, minerals, and flavonoids [31]. Of these, fiber is fermented by several bacteria to produce short-chain fatty acids (SCFAs), including acetate (central appetite regulation), propionate (gluconeogenesis and satiety signaling regulation), and butyrate (a main energy source for human colonocytes) [32, 33]. Higher fiber intake was associated with the increase of SCFAs, and SCFA-producing bacteria, which regulate the immune system and metabolism and reduce the CRC risk [33]. According to the WCRF/AICR, there was limited evidence for the effect of fruit and non-starchy vegetable intake on CRC prevention [34]. According to pooled estimates from prospective cohort studies, per daily 100 g of fruit and vegetable intakes were associated with a decreased risk of CRC by 4% (relative risk (RR) 0.96, 95% CI = 0.93–0.99) and 2% (RR = 0.98, 95% CI = 0.96–0.99), respectively [35]. However, individual studies tended to show null associations. A previous case–control analysis of nine observational studies within the Genetics Epidemiology of Colorectal Cancer Consortium and the Colon Cancer Family Registry did not observe any significant associations between fruit (odds ratio (OR) 1.04, 95% CI = 0.93–1.15) and vegetable (OR = 0.92, 95% CI = 0.82–1.03) intakes with overall CRC risk [36]. Similarly, nonsignificant associations between fruit (HR = 1.00, 95% CI = 0.94–1.05) and vegetable (HR = 1.01, 95% CI = 0.93–1.11) intakes and CRC risks were recently reported in a prospective cohort analysis of the UK Biobank [19]. These inconsistent findings with our MR estimates may be partly due to differences in study design and analytical framework. In general, observational studies are more prone to residual confounding, reverse causation, and measurement error than MR analyses, which randomly assign the exposure of interest-related IVs among individuals [4, 26]. Such sources of bias may attenuate associations toward the null [4, 26]. Furthermore, while the MR estimates reflect the effect of lifelong perturbations in risk factors, observational results may reflect more acute effects, during the follow-up period since the enrolment time point of a cohort) [37]. Our present observational analysis with a longer follow-up period (12.4 vs. 5.7 years) suggested stronger favorable effects of fruits (HR = 0.99, 95% CI = 0.91–1.01) and vegetables (HR = 0.99, 95% CI = 0.98–1.00), thus supports the evidence of long-term beneficial effects [19].

Among dietary factors, the International Agency for Research on Cancer classified processed meat as a human carcinogen (Group 1) and red meat as a probable carcinogen (Group 2A) [38]. Carcinogenic effects of red meat and processed meat were introduced via several chemicals such as *N*-nitroso compounds, heterocyclic aromatic amines, and polycyclic aromatic hydrocarbons formed in red meat and when cooking meat at high temperatures [39]. The WCRF/AICR also reported probable to convincing evidence of red meat and processed meat intake in association with CRC risks [34]. However, our present study observed the association between red meat and processed meat with CRC risk in observational analyses and multivariable MR. Besides differences in study design and analytical framework, the explained variation of IVs for the exposure of interest may affect our estimates. Although the allele score IVs explained variations of dietary intake (F-statistics greater than 90), the number of SNPs used for the calculation of allele scores for red meat and processed meat was relatively small, which may not allow us to detect any significant associations. We further observed an inverse association between processed meat intake and rectal cancer risk. These findings disappeared in sex-specific subgroups and need to be interpreted cautiously, possibly due to the small proportion of rectal cancer cases among whole study participants.

To date, very few MR studies reported the effect of dietary factors on CRC risk. Most of them considered blood concentrations of nutrients (carotenoids, calcium, copper, fatty acids, folate, iron, magnesium, methionine, phosphorus, selenium, sodium, vitamin B6, vitamin B12, vitamin D, vitamin E, and zinc) as exposure of interest [8, 40–44]. Only the MR study conducted by Cornish et al. examined the causal estimate between diet consumption of coffee and CRC risk. Although we used much more SNPs in the allele score calculation, our study revealed a similar direction of the estimates (33 SNPs, HR = 1.16, 95% CI = 0.96–1.40 in the current study vs. 4 SNPs, OR = 1.17, 95% CI = 0.88–1.55 in the previous study) [8].

Furthermore, we found inconclusive evidence of the MR estimates of total fish, milk, cheese, coffee, tea, and alcohol consumption on CRC. Of these, pooled estimates from observational studies showed significantly or suggestively inverse associations of fish (RR = 0.89, 95% CI = 0.80–0.99), milk (RR = 0.94, 95% CI = 0.92–0.96), cheese (RR = 0.94, 95% CI = 0.87–1.02), coffee (RR = 1.00, 95% CI = 0.99–1.02), tea (RR = 0.99, 95% CI = 0.97–1.01), and alcohol (RR = 1.07, 95% = 1.05–1.08) intake with CRC risk [35]. Compared to observational analysis, estimates from MR may commonly have wider CIs and thus toward null findings [37].

This study has several strengths. Having large-scale individual-level data with much more genetic information of imputed SNPs compared to earlier GWAS, we applied the recent methodology to account for confounding effects of both population stratification and cryptic relatedness to identify loci associated with food intake. We also performed a comprehensive MR analysis to suggest evidence for the causal estimate of dietary intake and CRC risk. Genetic variants had adequate strengths; thus, bias due to small *F*-statistics or small sample size can be minimised. Undertaking sensitivity analyses to evaluate the plausibility of IV assumptions and robustness to pleiotropy and outliers, our findings from MR analyses may be less biased by residual confounding and reverse causation than observational results. Additionally, combining many SNPs into a single allele score may increase the power of the analysis and reduce the risk of bias from possible weak instruments [26]. Furthermore, available data for one-sample MR analysis allowed us to consider the effect estimate in several subgroups, such as sex and CRC subsites.

Despite providing new evidence about the causal effect of dietary intake on CRC risk, this study has some limitations that need to be addressed. One limitation of the study is the fact that we analysed CRC risk only using the dietary information measured at a single time point, which may not reflect the lifelong dietary intake, thus, our findings were based on the assumption that such dietary habits might not change or be equally changed during follow-up. The effect of dietary factors might be underestimated due to random measurement errors [45]. Previous study investigated the reproducibility of the touchscreen questionnaire of average diet over the previous 12 months used in the current study with the 24-h dietary assessment [45]. Overall, the intra-correlation of food groups was reported to range between 0.38 to 0.63, which was comparable with the overall reproducibility of FFQs in nutritional epidemiology studies (macronutrients: 0.44–0.79; micronutrients: 0.51–0.74) [46]. However, among all participants completed the touchscreen questionnaire, only approximately 42% study participants provided the 24-h dietary assessment [45]. Nevertheless, our findings were limited for 24-h dietary data. Besides, given that disparities in dietary intake according to different ethnic groups may exist due to cultural knowledge and food-related skills [47, 48], analyses for individuals from ethnic backgrounds other than White British require additional investigations. Furthermore, we derived SNPs and weights for IVs in all participants after quality control and performed the two-stage least square analysis in participants without any cancer at baseline. There could still be a winner’s curse on our estimate due to the overlap between the dataset in which genetic variants were selected and the dataset in which genetically predicted associations were determined [49]. However, the winner’s curse bias in our study can be mitigated by selecting more stringent SNPs based on not only significant threshold but also linkage disequilibrium among variants. Moreover, to obtain GWAS-identified variants for the MR analysis, our study assumed linear associations between dietary intake and risk of developing CRC.

## Conclusion

In summary, the present study comprehensively assessed the influence of genetic variants and their functional mechanisms on the dietary behaviors of participants in the UK Biobank. By cautiously accounting for population stratification and cryptic relatedness in this large-scale of recently released imputation data, we identified several loci for food consumption. These genetic variants associated were used as IVs in the MR framework to address the relationship between dietary intake and CRC risk. Our findings supported a relationship between fruit intake and a decreased risk of CRC and suggested an effective strategy of consuming fruits in the primary prevention of CRC. Further studies in individuals from ethnic backgrounds other than White British are needed to validate our findings.

## Supplementary Information

Supplementary Material 1.

Supplementary Material 2.

Supplementary Material 3.

## Data Availability

The UK Biobank is an open access resource, available at https://www.ukbiobank.ac.uk/researchers/. Data used in this project can be obtained from the UK Biobank by submitting a data request proposal.

## Abbreviations

GWAS: Genome-wide association study
MR: Mendelian randomisation
IV: Instrumental variable
WCRF: World Cancer Research Fund
AICR: American Institute for Cancer Research
FFQ: Food frequency questionnaire
SNP: Single nucleotide polymorphism
POLMM: proportional odds logistic mixed model
HR: Hazard ratio
RR: Relative risk
CI: Confidence interval
PC: Principal component
