# Supplementary material for: Genetically predicted dietary intake and risks of colorectal cancer: a Mendelian randomisation study
Source: BMC Cancer. 2024 Sep 17;24:1153. doi: 10.1186/s12885-024-12923-1 (PMC11409808; doi:10.1186/s12885-024-12923-1)
Supplement: Supplementary file 1 — Supplementary Material 1. [file 12885_2024_12923_MOESM1_ESM.docx]

**Additional file 1: Supplementary Information and Method**

**eInformation**

Nutrigenetics is defined the science that explores the interactions between genetic factors and the inter-individual variability of the disease response to specific diets (1, 2). To date, findings from twin studies suggested that the selection of diets was partially contributed by genetic effects (3-5). The heritability of food preference was reported between 0.36 to 0.54 according to food items as follows (3-5):

| **Study** | **Population** | **Food item** | **Heritability** |
| --- | --- | --- | --- |
| Smith et al., 2016 (5) | 2,865 British twins aged 18-19 years | Meat or fish  Dairy  Fruits  Vegetables | 0.49  0.44  0.49  0.54 |
| Pallister et al., 2015 (4) | 2,569 UK twins aged 19 to 88 years | Meat  Fruits and vegetables | 0.52  0.36 |
| Breen et al., 2006 (3) | 214 UK twins aged 4 to 5 years | Fruits  Vegetables | 0.51-0.53  0.37-0.54 |

Several genes and corresponding polymorphisms which link to eating preferences, food addiction, taste sensations, fat and carbohydrate absorption, food intolerances, vitamin metabolism, and xenobiotic metabolism have been identified from studies of gene candidate approach (6):

| **Function** | **Gene (polymorphism)** |
| --- | --- |
| Eating preferences | *FTO* (rs9939609), *MC4R* (rs17782313), *DRD2* (rs1800497) |
| Food addiction | *ADH1B* (rs1229984), *ALDH2* (rs671), *CHRNA5* (rs16969968), *CHRNA3* (rs1051730) |
| Taste sensations | *GLUT2* (rs5400), *TAS2R38* (rs1726866), *CD36* (rs1761667), *ADD1* (rs4961), *CYP11B2* (rs1799998) |
| Fat and carbohydrate absorption | *ADRB2* (rs1042714, rs1042713), *TCF7L2* (rs12255372, rs7903146), *FABP2* (rs1799883), *PPARG* (rs1801282), *CETP* (rs5882), *ADRB3* (rs4994), *ApoA5* (rs662799, rs3135506), *LEPR* (rs1137101), *ApoE* (rs429358, rs7412) |
| Food intolerances | *HLA-DQ* (*HLA*-*DQA1 HLA*-*DQB1*), *MCM6* (rs4988235) |
| Vitamin metabolism | *BCMO1* (rs7501331, rs12934922, rs119478057), *ALPL* (rs1256335), *NBPF3* (rs4654748), *MTHFR* (rs1801133), *FUT2* (rs602662), *VDR* (rs1544410), *GC* (rs2282679), *FADS1* (rs174547) |
| Xenobiotic metabolism | *MnSOD* (rs4880), *GSTP1* (rs947894), *CYP1A2* (rs762551) |

In particular, individual food preferences and dietary habits have been identified to be affected by the senses of taste and smell as well as metabolic processes (7). Haplotypes of polymorphisms in the *TAS2R38* gene (rs1726866, rs10246939, and rs713598), which contributed to individual difference in bitter taste perception of 6-n-propylthiouracil and phenylthiocarbamide, were shown to be linked with cured meat, brassica and cruciferous vegetables, and green tea consumption (8). Analysis of bitter receptor *TAS2R42* gene, which involved in bitter aftertaste of sulfonyl amide sweeteners, showed the correlation between its variants and coffee liking (9). Besides, several taste receptors have been identified within taste cell membranes on the surface of the tongue, including *T2R* family for bitter, *T1R* for sweet and umami, ion channels *PKD1L3* and *PKD2L1* for sour taste, and epithelial sodium channels and *TRPV1* for salty taste perceptions (10). Individuals who have enhanced perception of bitter taste may avoid the intake of specific fruits and vegetables, which contain many bitter-tasting flavonoids and polyphenols (11). Bitter taste perception of propylthiouracil (rs1726866), quinine (rs10772420), and caffeine (rs2597979) was further determined to be associated with the consumption of bitter beverages, including coffee and tea (12).

A functional chemosensory receptor of *TAAR5* gene was found to express in olfactory mucosa and affect odor perception via the p.Ser95Pro sequence variant (13). *TAAR5* variant carriers tended to rate the fish odor as less unpleasant, but more likely to use non-seafood-related descriptors with neutral or positive odor valence, and thus more likely to consume fish (13). A recent high-throughput sequencing of the whole olfactory receptor extensively determined several genetic variations associated with different phenotypes (14). Among olfactory receptor genes, SNPs of the *OR7D4* gene, which are responsible for two amino acid substitutions that reduce androstenone perception, were found to be associated with the preference of pork meat containing androstenone rather than other meats (14-16). *Cis*-3-hexen-1-ol is an odorant with green or grassy smell and presents in several fruits and vegetables. Another olfactory gene, *OR2J3*, which involved in individual variations in the detection of *cis*-3-hexen-1-ol, might thus correlated with differences in fruits and vegetable preferences (17).

Furthermore, mutations of genes involving in the metabolism of foods also affected food choice behaviors. In marine fish, trimethylamine-*N*-oxide presents with substantial amounts, and is metabolized by gut bacteria into trimethylamine, which is then oxidized by *FMO3* liver enzyme (18). When *FMO3* is mutated, trimethylamine will be accumulated, which results in the characteristic smell of rotting fish and presents people from consuming fish (19). Another example is the presence of the lactose-digesting enzyme lactase in the small intestine, which make people drink milks without any discomforts (20, 21). However, when there is the lactase persistence or lactose tolerance, which is defined when there are mutations in lactase gene (e.g., LCT-13910 C>T, A-22018, C-14010, G-13907, and G-13915) and lactase activity is more than 10 units per gram protein, people are less likely to consume milks (20, 21).

**Additional file 1: eMethod**

*Genome-wide association analyses*

In the genome-wide association between SNPs and food intake phenotypes, beta coefficients were estimated for each increment of the minor allele in a linear mixed model framework (22, 23). Accordingly, analyses were adjusted for age, sex, and the first 6 genetic principal component scores, which were released by the UK Biobank and defined the White British ancestry subset (24), as fixed effects.

To quantify the cryptic relatedness for the variance of genetic effects, we extracted genotyping data of 93,183 SNPs, which were used for the final kinship inference (n=93,511) (24) and available after our quality control process (n=27,503,596). These markers were used to estimate the GRM of pairwise relatedness between individuals. A cutoff value of 0.05 was selected to remove one of a pair of individuals with a relatedness greater than 0.05 (25). The computed GRM was included as a random effect in the linear mixed effect model.

GWA analyses under the linear mixed model were conducted using the fastGWA tool (23), and the Manhattan plot of summary statistics was created in the R program (package ‘qqman’ (26)). The results were further compared with findings from the plink2 tool (27, 28), which did not account for individual relatedness.

*Genomic risk loci and regional annotation*

The identification of genomic risk loci and SNP annotation were performed by the SNP2GENE of the web-based FUMA tool (29). Using summary statistics from the GWAS, independent significant SNPs were identified as those with p<5x10^-8^ and r^2^<0.6. Those with r^2^<0.1 were then defined as lead SNPs, and of these, SNPs that were in linkage disequilibrium (LD) with independent significant SNPs (r^2^≥0.6) were defined as genomic risk loci. The 1000G Phase3 UER was used as the reference panel population, and the maximum distance between LD blocks to merge into a genomic locus was set at 250 kb (29).

To map candidate SNPs to genes, three methods were used, including positional, eQTL, and chromatin interaction mapping. For positional mapping, SNPs were mapped to genes based on annotations from ANNOVAR. For eQTL mapping, both independent significant SNPs and their LD SNPs were mapped to eQTLs in types of user-defined tissues. For chromatin interaction mapping, overlapping SNPs of independent significant variants and their LD variants were mapped to genes with promoters that overlapped with another end of significant interactions (29).

*Functional analysis*

For each input gene, the GENE2FUNC of the FUMA tool provided the enrichment of public gene sets and tested the representation in different functional gene sets using databases such as the Molecular Signatures Database (MsigDB) and the WikiPathways (29). Individual genes that shared certain biological functions were grouped and evaluated for associations with the dietary trait. The cutoff for adjusted p values was set at 0.05 and minimum overlapping genes with gene sets were assigned at 2 in the functional analysis to detect significant gene ontology biological processes and molecular functions and WikiPathways.

*Estimation of heritability and genetic and phenotypic correlation*

The tagging file, which records the expected heritability tagged by each SNP, was obtained for the LDAK-Thin model (30). Summary statistics from the genome-wide association analysis for food intake together with the tagged file were used to estimate the heritability (h^2^) and genetic correlation (r^2^) of food intake (30). Pearson correlation coefficients for the consumption between two dietary factors were calculated and visualized as a heatmap using the R program (package ‘ggplot2’ (31)).

*Effect estimates for associations between genetic variants with CRC incidence adjusting for familial relatedness*

Beta coefficients for the effect of variants on CRC risks were estimated by fitting a frailty model approach of the Genetic Analysis of Time-to-Event (GATE) outcome (32). In GATE, a null frailty model was first fitted to compute the variance component ratio and other parameters including GRM elements (32). Then, score statistics were applied to test the association between each variant and CRC (32). The model was also adjusted for age, sex, and the first 6 first principal component scores.

**References**

1. de Toro-Martin J, Arsenault BJ, Despres JP, Vohl MC. Precision nutrition: a review of personalized nutritional approaches for the prevention and management of metabolic syndrome. Nutrients. 2017;9(8).

2. Barrea L, Annunziata G, Bordoni L, Muscogiuri G, Colao A, Savastano S, et al. Nutrigenetics-personalized nutrition in obesity and cardiovascular diseases. Int J Obes Suppl. 2020;10(1):1-13.

3. Breen FM, Plomin R, Wardle J. Heritability of food preferences in young children. Physiol Behav. 2006;88(4-5):443-7.

4. Pallister T, Sharafi M, Lachance G, Pirastu N, Mohney RP, MacGregor A, et al. Food preference patterns in a UK Twin Cohort. Twin Res Hum Genet. 2015;18(6):793-805.

5. Smith AD, Fildes A, Cooke L, Herle M, Shakeshaft N, Plomin R, et al. Genetic and environmental influences on food preferences in adolescence. Am J Clin Nutr. 2016;104(2):446-53.

6. Vesnina A, Prosekov A, Kozlova O, Atuchin V. Genes and eating preferences, their roles in personalized nutrition. Genes (Basel). 2020;11(4).

7. Boesveldt S, de Graaf K. The differential role of smell and taste for eating behavior. Perception. 2017;46(3-4):307-19.

8. Calancie L, Keyserling TC, Taillie LS, Robasky K, Patterson C, Ammerman AS, et al. TAS2R38 predisposition to bitter taste associated with differential changes in vegetable Intake in response to a community-based dietary intervention. G3 (Bethesda). 2018;8(6):2107-19.

9. Pirastu N, Kooyman M, Traglia M, Robino A, Willems SM, Pistis G, et al. Association analysis of bitter receptor genes in five isolated populations identifies a significant correlation between TAS2R43 variants and coffee liking. PLoS One. 2014;9(3):e92065.

10. Garcia-Bailo B, Toguri C, Eny KM, El-Sohemy A. Genetic variation in taste and its influence on food selection. OMICS. 2009;13(1):69-80.

11. Dotson CD, Babich J, Steinle NI. Genetic predisposition and taste preference: impact on food intake and risk of chronic disease. Genetics. 2012;1:175-83.

12. Ong JS, Hwang LD, Zhong VW, An J, Gharahkhani P, Breslin PAS, et al. Understanding the role of bitter taste perception in coffee, tea and alcohol consumption through Mendelian randomization. Sci Rep. 2018;8(1):16414.

13. Gisladottir RS, Ivarsdottir EV, Helgason A, Jonsson L, Hannesdottir NK, Rutsdottir G, et al. Sequence variants in TAAR5 and other loci affect human odor perception and naming. Curr Biol. 2020;30(23):4643-53 e3.

14. Trimmer C, Keller A, Murphy NR, Snyder LL, Willer JR, Nagai MH, et al. Genetic variation across the human olfactory receptor repertoire alters odor perception. Proc Natl Acad Sci U S A. 2019;116(19):9475-80.

15. Robino A, Concas MP, Catamo E, Gasparini P. A brief review of genetic approaches to the study of food preferences: current knowledge and future directions. Nutrients. 2019;11(8).

16. Lunde K, Egelandsdal B, Skuterud E, Mainland JD, Lea T, Hersleth M, et al. Genetic variation of an odorant receptor OR7D4 and sensory perception of cooked meat containing androstenone. PLoS One. 2012;7(5):e35259.

17. McRae JF, Mainland JD, Jaeger SR, Adipietro KA, Matsunami H, Newcomb RD. Genetic variation in the odorant receptor OR2J3 is associated with the ability to detect the "grassy" smelling odor, cis-3-hexen-1-ol. Chem Senses. 2012;37(7):585-93.

18. Nanayakkara PWB, Meijboom M, Kramer MHH, Wevers RA, Jakobs C. What have we here? A man or a fish? Lancet. 2010;376(9753):1710.

19. Figueiredo JC, Hsu L, Hutter CM, Lin Y, Campbell PT, Baron JA, et al. Genome-wide diet-gene interaction analyses for risk of colorectal cancer. PLoS Genet. 2014;10(4):e1004228.

20. Comerford KB, Pasin G. Gene-dairy food interactions and health outcomes: a review of nutrigenetic studies. Nutrients. 2017;9(7).

21. Travis RC, Appleby PN, Siddiq A, Allen NE, Kaaks R, Canzian F, et al. Genetic variation in the lactase gene, dairy product intake and risk for prostate cancer in the European prospective investigation into cancer and nutrition. Int J Cancer. 2013;132(8):1901-10.

22. Yang J, Lee SH, Goddard ME, Visscher PM. GCTA: a tool for genome-wide complex trait analysis. Am J Hum Genet. 2011;88(1):76-82.

23. Jiang L, Zheng Z, Qi T, Kemper KE, Wray NR, Visscher PM, et al. A resource-efficient tool for mixed model association analysis of large-scale data. Nat Genet. 2019;51(12):1749-55.

24. Bycroft C, Freeman C, Petkova D, Band G, Elliott LT, Sharp K, et al. The UK Biobank resource with deep phenotyping and genomic data. Nature. 2018;562(7726):203-9.

25. Yang J, Benyamin B, McEvoy BP, Gordon S, Henders AK, Nyholt DR, et al. Common SNPs explain a large proportion of the heritability for human height. Nat Genet. 2010;42(7):565-9.

26. Turner S. Package ‘qqman’: Q-Q and Manhattan plots for GWAS data (version 0.1.8) 2022 [Available from: <https://github.com/stephenturner/qqman>.

27. Purcell S, Chang C. plink2 [Available from: <www.cog-genomics.org/plink/2.0/>.

28. Chang CC, Chow CC, Tellier LC, Vattikuti S, Purcell SM, Lee JJ. Second-generation PLINK: rising to the challenge of larger and richer datasets. Gigascience. 2015;4:7.

29. Watanabe K, Taskesen E, van Bochoven A, Posthuma D. Functional mapping and annotation of genetic associations with FUMA. Nat Commun. 2017;8(1):1826.

30. Speed D, Kaphle A, Balding DJ. SNP-based heritability and selection analyses: Improved models and new results. Bioessays. 2022;44(5):e2100170.

31. Wickham H, Chang W, Henry L, Pedersen TL, Takahashi K, Wilke C, et al. Package ‘ggplot2’: Create elegant data visualisations using the grammar of graphics 2022 [Available from: <https://ggplot2.tidyverse.org>.

32. Dey R, Zhou W, Kiiskinen T, Havulinna A, Elliott A, Karjalainen J, et al. Efficient and accurate frailty model approach for genome-wide survival association analysis in large-scale biobanks. Nat Commun. 2022;13(1):5437.
