# Supplementary material for: Genetically predicted dietary intake and risks of colorectal cancer: a Mendelian randomisation study
Source: BMC Cancer. 2024 Sep 17;24:1153. doi: 10.1186/s12885-024-12923-1 (PMC11409808; doi:10.1186/s12885-024-12923-1)
Supplement: Supplementary file 2 — Supplementary Material 2. [file 12885_2024_12923_MOESM2_ESM.docx]

**Additional file 2: Supplementary Tables and Figures**

**Table S1.** Summary statistics of instrumental variables for their associations with dietary intake and colorectal cancer risk

| **Chr** | **Variant** | **Position** | **Ref. / Alt. allele** | **Nearest gene** | **Instrument - exposure** | | | **F-statistic** | **Instrument - outcome** | | |
| --- | --- | --- | --- | --- | --- | --- | --- | --- | --- | --- | --- |
|  |  |  |  |  | **Beta** | **SE** | **P-value** |  | **Beta** | **SE** | **P-value** |
| **Red meat** | | | | | | | | | | | |
| 1 | rs2055145 ^†^ | 45926495 | C/G | *TESK2* | 0.022 | 0.0036 | 2.06e-09 | 36 | -0.074 | 0.0249 | 0.003 |
| 1 | rs10789340 | 72940273 | A/G | *RPL31P12* | 0.020 | 0.0032 | 8.15e-10 | 38 | 0.004 | 0.0216 | 0.870 |
| 1 | rs11210240 | 73896101 | C/A | *RP4-598G3.1* | 0.022 | 0.0039 | 2.46e-08 | 31 | 0.000 | 0.0271 | 0.997 |
| 1 | rs12144834 | 97352352 | T/G | *AL592205.1* | -0.068 | 0.0118 | 1.08e-08 | 33 | -0.014 | 0.0857 | 0.871 |
| 2 | rs1451077 | 147901207 | G/A | *snoU13* | 0.019 | 0.0031 | 2.63e-09 | 35 | 0.035 | 0.0213 | 0.102 |
| 3 | rs61791721 ^†^ | 135804550 | T/A | *PPP2R3A* | 0.021 | 0.0037 | 7.51e-09 | 33 | -0.050 | 0.0252 | 0.049 |
| 5 | rs576361041 | 43358175 | G/A | *RP11-159F24.2* | -0.726 | 0.1329 | 4.73e-08 | 30 | 0.283 | 3.4493 | 0.935 |
| 5 | 5:124101388 | 124101388 | ATAT/A | *ZNF608* | 0.033 | 0.0059 | 1.48e-08 | 32 | 0.045 | 0.0412 | 0.272 |
| 6 | rs4486004 | 26167710 | G/T | *HIST1H2BD* | 0.025 | 0.0037 | 1.09e-11 | 46 | -0.036 | 0.0255 | 0.158 |
| 7 | rs554034302 | 138227194 | G/T | *TRIM24* | -0.267 | 0.0487 | 4.12e-08 | 30 | -0.200 | 0.3579 | 0.575 |
| 9 | rs141229573 | 15576114 | T/TATC | *CCDC171* | 0.028 | 0.0031 | 7.74e-20 | 83 | -0.028 | 0.0209 | 0.175 |
| 13 | rs150877559 | 50701733 | G/A | *DLEU1* | -0.054 | 0.0098 | 4.83e-08 | 30 | -0.036 | 0.0677 | 0.593 |
| 16 | rs12931387 | 5676432 | A/C | *RP11-420N3.2* | -0.025 | 0.0039 | 2.26e-10 | 40 | -0.011 | 0.0269 | 0.682 |
| 17 | rs12938702 | 74607174 | C/T | *ST6GALNAC1* | 0.022 | 0.0037 | 5.70e-09 | 34 | 0.003 | 0.0242 | 0.912 |
| 19 | rs7251466 | 42576952 | G/C | *ZNF574* | -0.030 | 0.0049 | 2.29e-09 | 36 | 0.025 | 0.0339 | 0.459 |
| **Processed meat** | | | | | | | | | | | |
| 2 | rs17676243 | 173322809 | G/A | *ITGA6* | 0.020 | 0.0037 | 3.59e-08 | 30 | -0.040 | 0.0264 | 0.132 |
| 3 | rs9809856 | 18227421 | A/G | *TBC1D5* | -0.018 | 0.0030 | 2.11e-09 | 36 | -0.017 | 0.0211 | 0.407 |
| 3 | rs6765179 | 25276416 | G/A | *RARB* | 0.018 | 0.0032 | 9.07e-09 | 33 | 0.016 | 0.0228 | 0.492 |
| 3 | rs13091492 | 81891476 | A/G | *RP11-359D24.1* | -0.019 | 0.0031 | 1.40e-09 | 37 | 0.031 | 0.0218 | 0.151 |
| 4 | rs7695118 | 149012794 | A/G | *NR3C2* | 0.018 | 0.0030 | 1.51e-09 | 37 | -0.019 | 0.0210 | 0.367 |
| 8 | rs2980508 | 8171732 | G/A | *SGK223* | 0.019 | 0.0030 | 5.76e-11 | 43 | -0.018 | 0.0211 | 0.397 |
| 8 | rs113442811 | 10772255 | C/CACA  GAAGA | *XKR6* | -0.023 | 0.0030 | 9.45e-15 | 60 | 0.032 | 0.0211 | 0.128 |
| 9 | rs147845411 ^†^ | 24756659 | G/T | *AL157830.1* | -0.066 | 0.0120 | 3.68e-08 | 30 | -0.176 | 0.0899 | 0.050 |
| 11 | rs11030328 | 28447142 | G/A | *RP11-22P4.1* | -0.017 | 0.0030 | 1.84e-08 | 32 | 0.016 | 0.0211 | 0.456 |
| 11 | rs61880662 | 30901989 | A/G | *DCDC1* | 0.025 | 0.0043 | 9.52e-09 | 33 | -0.001 | 0.0312 | 0.965 |
| 11 | rs11032362 ^*^ | 33759092 | G/A | *CD59* | 0.032 | 0.0052 | 3.61e-10 | 39 | 0.009 | 0.0368 | 0.811 |
| 19 | rs8103840 ^*,†^ | 49254955 | C/T | *FUT1* | -0.020 | 0.0030 | 5.90e-11 | 43 | 0.042 | 0.0209 | 0.046 |
| **Total fish** | | | | | | | | | | | |
| 1 | rs1460943 | 72813129 | C/T | *RPL31P12* | -0.020 | 0.0035 | 2.31e-08 | 31 | 0.009 | 0.0215 | 0.677 |
| 1 | rs12566777 ^†^ | 112590831 | T/C | *RP11-88H9.2* | 0.021 | 0.0036 | 3.85e-09 | 35 | 0.057 | 0.0222 | 0.010 |
| 2 | rs4600686 | 79145715 | T/C | *RNU6-812P* | 0.026 | 0.0047 | 2.96e-08 | 31 | -0.035 | 0.0287 | 0.219 |
| 3 | rs6577598 | 17885531 | T/A | *TBC1D5* | -0.021 | 0.0035 | 1.17e-09 | 37 | -0.008 | 0.0210 | 0.705 |
| 3 | rs12630658 | 25122238 | T/C | *AC133680.1* | -0.020 | 0.0035 | 6.46e-09 | 34 | 0.000 | 0.0211 | 0.993 |
| 3 | 3:114916925 | 114916925 | CT/C | *ZBTB20* | 0.021 | 0.0038 | 4.98e-08 | 30 | -0.027 | 0.0224 | 0.228 |
| 5 | rs469250 | 102140615 | G/C | *PAM* | 0.020 | 0.0036 | 4.48e-08 | 30 | 0.023 | 0.0219 | 0.288 |
| 6 | rs3734543 | 26468545 | G/C | *BTN2A1* | 0.037 | 0.0053 | 1.81e-12 | 50 | -0.008 | 0.0323 | 0.800 |
| 7 | rs11767283 ^†^ | 121947456 | A/G | *FEZF1:FEZF1-*  *AS1* | -0.023 | 0.0042 | 4.99e-08 | 30 | -0.065 | 0.0257 | 0.012 |
| 8 | 8:8661026 | 8661026 | CA/C | *MFHAS1* | -0.022 | 0.0035 | 2.65e-10 | 40 | 0.017 | 0.0208 | 0.422 |
| 8 | rs9650651 | 10267540 | C/A | *MSRA* | -0.023 | 0.0035 | 6.49e-11 | 43 | -0.002 | 0.0214 | 0.931 |
| 8 | rs5891918 | 64603887 | G/GT | *RP11-32K4.1* | -0.024 | 0.0039 | 1.14e-09 | 37 | -0.003 | 0.0224 | 0.899 |
| 10 | rs12251016 | 21821918 | A/T | *MLLT10* | 0.024 | 0.0036 | 1.68e-11 | 45 | -0.028 | 0.0223 | 0.217 |
| 11 | rs535669 | 28729030 | A/G | *RP11-115J23.1* | 0.020 | 0.0037 | 4.99e-08 | 30 | 0.010 | 0.0229 | 0.658 |
| 12 | rs35287743 | 110057250 | G/T | *MVK* | 0.048 | 0.0054 | 1.17e-18 | 78 | -0.014 | 0.0334 | 0.669 |
| 13 | rs7336980 | 59437165 | C/G | *DNAJA1P1* | 0.023 | 0.0039 | 2.16e-09 | 36 | -0.021 | 0.0237 | 0.378 |
| 14 | rs7146955 | 29750244 | A/G | *RP11-562L8.1* | 0.022 | 0.0035 | 2.66e-10 | 40 | 0.039 | 0.0215 | 0.070 |
| 14 | rs200738790 | 80721903 | G/GA | *DIO2:DIO2-AS1* | 0.409 | 0.0745 | 3.93e-08 | 30 | -0.446 | 0.4901 | 0.363 |
| 14 | rs12887132 | 100284679 | C/G | *EML1* | 0.020 | 0.0035 | 2.41e-08 | 31 | 0.016 | 0.0215 | 0.466 |
| 16 | rs9889161 | 51495068 | G/T | *RP11-437L7.1* | 0.022 | 0.0036 | 1.49e-09 | 37 | -0.027 | 0.0220 | 0.217 |
| 16 | rs7187250 | 53810546 | C/A | *FTO* | -0.026 | 0.0035 | 1.35e-13 | 55 | -0.020 | 0.0216 | 0.364 |
| 16 | rs11859365 | 83683945 | A/C | *CDH13* | -0.025 | 0.0040 | 1.64e-10 | 41 | 0.025 | 0.0243 | 0.310 |
| 18 | rs7238896 | 1840658 | A/G | *RP11-161I6.2* | -0.030 | 0.0050 | 2.31e-09 | 36 | -0.004 | 0.0307 | 0.897 |
| 18 | rs7240986 | 53195249 | G/A | *TCF4* | -0.021 | 0.0036 | 7.40e-09 | 33 | 0.009 | 0.0219 | 0.680 |
| 18 | rs242641 | 58853208 | C/T | *CDH20* | 0.021 | 0.0038 | 1.29e-08 | 32 | 0.003 | 0.0230 | 0.889 |
| 19 | rs429358 ^*^ | 45411941 | T/C | *APOE* | -0.038 | 0.0047 | 1.16e-15 | 64 | -0.051 | 0.0292 | 0.078 |
| 19 | rs8103840 ^†^ | 49254955 | C/T | *FUT1* | -0.028 | 0.0035 | 1.34e-15 | 64 | 0.042 | 0.0209 | 0.046 |
| 22 | 22:31871253 | 31871253 | GA/G | *DRG1:EIF4ENIF1* | -0.020 | 0.0038 | 4.83e-08 | 30 | -0.014 | 0.0229 | 0.529 |
| **Milk** | | | | | | | | | | | |
| 1 | rs201406724 ^*^ | 150781915 | T/TA | *ARNT* | -0.017 | 0.0024 | 5.41e-12 | 48 | 0.026 | 0.0222 | 0.249 |
| 1 | rs11264235 | 154631081 | C/T | *ADAR* | -0.016 | 0.0025 | 7.03e-10 | 38 | 0.024 | 0.0236 | 0.316 |
| 1 | rs2901785 | 174104743 | G/A | *RP11-160H22.5* | 0.018 | 0.0023 | 9.65e-16 | 65 | 0.016 | 0.0212 | 0.463 |
| 2 | 2:27748992 ^*^ | 27748992 | AT/A | *GCKR* | -0.016 | 0.0024 | 6.51e-12 | 47 | -0.036 | 0.0212 | 0.089 |
| 2 | rs183195584 | 48345073 | T/A | *AC079807.4* | 0.075 | 0.0133 | 1.55e-08 | 32 | -0.027 | 0.1407 | 0.847 |
| 2 | 2:58364214 | 58364214 | CAT/C | *VRK2* | 0.015 | 0.0026 | 9.98e-09 | 33 | -0.022 | 0.0239 | 0.366 |
| 2 | rs4611605 | 157112515 | A/T | *NR4A2* | -0.015 | 0.0026 | 1.06e-08 | 33 | 0.013 | 0.0246 | 0.604 |
| 3 | rs13082065 | 35731993 | C/T | *ARPP21* | -0.013 | 0.0023 | 2.77e-08 | 31 | -0.019 | 0.0214 | 0.378 |
| 3 | rs57462170 | 50239803 | G/A | *SLC38A3* | -0.020 | 0.0037 | 2.06e-08 | 31 | -0.045 | 0.0339 | 0.179 |
| 4 | rs11940694 ^*^ | 39414993 | A/G | *KLB* | 0.015 | 0.0023 | 1.84e-10 | 41 | -0.014 | 0.0211 | 0.506 |
| 4 | rs6819372 | 67970101 | A/G | *RNU6-699P* | 0.013 | 0.0023 | 1.33e-08 | 32 | 0.028 | 0.0211 | 0.190 |
| 4 | rs2199936 ^*^ | 89045331 | A/G | *ABCG2* | -0.041 | 0.0036 | 9.59e-30 | 128 | -0.020 | 0.0329 | 0.536 |
| 5 | rs7730403 | 7429254 | G/A | *ADCY2* | 0.014 | 0.0023 | 1.79e-09 | 36 | -0.015 | 0.0213 | 0.471 |
| 5 | rs12518404 | 60341996 | T/C | *NDUFAF2* | -0.015 | 0.0023 | 1.24e-10 | 41 | -0.002 | 0.0213 | 0.920 |
| 5 | rs12658032 | 103904226 | A/G | *RP11-6N13.1* | -0.013 | 0.0024 | 1.56e-08 | 32 | 0.008 | 0.0220 | 0.729 |
| 5 | 5:124728469 | 124728469 | AG/A | *RP11-395P13.6* | -0.014 | 0.0024 | 6.40e-09 | 34 | -0.032 | 0.0219 | 0.144 |
| 5 | rs35124713 | 151951108 | G/A | *AC091969.1* | 0.015 | 0.0026 | 4.45e-09 | 34 | 0.008 | 0.0238 | 0.726 |
| 6 | rs2465018 ^*^ | 51241140 | G/A | *RP3-437C15.2* | -0.019 | 0.0027 | 3.56e-12 | 48 | 0.007 | 0.0253 | 0.786 |
| 6 | rs9490002 | 98331134 | G/A | *RP11-436D23.1* | -0.015 | 0.0023 | 2.54e-10 | 40 | 0.024 | 0.0211 | 0.247 |
| 6 | rs1101559 | 127047683 | C/T | *RPS4XP9* | 0.013 | 0.0023 | 3.99e-08 | 30 | 0.007 | 0.0211 | 0.730 |
| 6 | rs139797380 | 137244957 | C/G | *SLC35D3* | 0.098 | 0.0133 | 1.93e-13 | 54 | -0.077 | 0.1354 | 0.569 |
| 7 | rs4410790 ^*^ | 17284577 | T/C | *AC003075.4* | -0.065 | 0.0024 | 4.72e-164 | 745 | -0.015 | 0.0220 | 0.503 |
| 7 | rs1476765 | 32320458 | G/T | *PDE1C* | 0.013 | 0.0024 | 4.92e-08 | 30 | 0.024 | 0.0208 | 0.254 |
| 7 | 7:73042302 | 73042302 | GCTTT/  G | *MLXIPL* | -0.036 | 0.0034 | 2.57e-26 | 113 | -0.019 | 0.0313 | 0.544 |
| 7 | rs17685 ^*^ | 75616105 | G/A | *POR* | -0.035 | 0.0025 | 3.45e-42 | 185 | 0.006 | 0.0235 | 0.792 |
| 8 | 8:73433232 ^*^ | 73433232 | GGTA/G | *KCNB2* | -0.014 | 0.0025 | 4.31e-08 | 30 | -0.009 | 0.0227 | 0.697 |
| 8 | rs2737216 | 116630079 | A/T | *TRPS1* | 0.014 | 0.0023 | 3.26e-09 | 35 | -0.028 | 0.0210 | 0.176 |
| 10 | 10:87339574 | 87339574 | CA/C | *GRID1* | 0.014 | 0.0023 | 4.36e-09 | 34 | 0.000 | 0.0211 | 0.986 |
| 10 | rs10786069 | 94845546 | C/T | *CYP26A1* | 0.016 | 0.0023 | 8.18e-12 | 47 | -0.039 | 0.0211 | 0.062 |
| 10 | rs12256016 | 131476007 | A/G | *MGMT* | -0.016 | 0.0026 | 1.01e-09 | 37 | -0.021 | 0.0242 | 0.385 |
| 11 | rs71480157 | 27610041 | T/C | *BDNF-AS* | 0.019 | 0.0030 | 3.91e-10 | 39 | 0.046 | 0.0276 | 0.093 |
| 11 | rs78059714 | 46157568 | C/T | *RP11-702F3.2* | -0.017 | 0.0030 | 2.87e-08 | 31 | 0.001 | 0.0279 | 0.983 |
| 12 | 12:111854285 | 111854285 | GT/G | *SH2B3* | -0.018 | 0.0029 | 1.40e-10 | 41 | -0.010 | 0.0268 | 0.699 |
| 12 | rs7957424 ^†^ | 120894318 | G/A | *AL021546.6:GATC* | 0.013 | 0.0023 | 1.81e-08 | 32 | 0.049 | 0.0210 | 0.020 |
| 13 | rs2937338 | 55999207 | A/G | *MIR5007* | 0.015 | 0.0026 | 8.38e-09 | 33 | 0.028 | 0.0236 | 0.231 |
| 14 | rs71101691 | 29716334 | C/CT | *RP11-562L8.1* | -0.013 | 0.0023 | 4.35e-08 | 30 | -0.011 | 0.0210 | 0.606 |
| 14 | rs4509952 | 98869431 | G/A | *RN7SL714P* | -0.013 | 0.0023 | 3.92e-08 | 30 | -0.003 | 0.0215 | 0.886 |
| 15 | rs12591786 ^*^ | 60902512 | C/T | *RP11-219B17.1:RORA* | 0.020 | 0.0032 | 1.57e-10 | 41 | 0.027 | 0.0297 | 0.369 |
| 15 | rs12909335 ^*^ | 75214789 | T/A | *COX5A* | -0.039 | 0.0023 | 1.71E-63 | 283 | 0.016 | 0.3902 | 0.967 |
| 16 | 16:18840320 | 18840320 | CA/C | *SMG1* | 0.016 | 0.0023 | 1.60e-11 | 45 | -0.003 | 0.0215 | 0.892 |
| 16 | rs378421 | 28754684 | G/A | *RP11-57A19.4* | -0.014 | 0.0023 | 4.62e-09 | 34 | 0.027 | 0.0213 | 0.205 |
| 16 | rs62039910 | 64694053 | G/A | *RP11-467L24.1* | 0.023 | 0.0042 | 3.56e-08 | 30 | 0.053 | 0.0390 | 0.170 |
| 17 | rs12600469 | 40834073 | G/T | *CCR10* | -0.016 | 0.0024 | 2.28e-11 | 45 | -0.022 | 0.0218 | 0.307 |
| 18 | 18:57835314 | 57835314 | CT/C | *RNU4-17P* | -0.016 | 0.0025 | 2.58e-10 | 40 | 0.014 | 0.0226 | 0.538 |
| 19 | rs11555274 | 13207284 | G/C | *NFIX* | -0.020 | 0.0035 | 9.78e-09 | 33 | 0.036 | 0.0330 | 0.281 |
| 19 | rs3764567 | 19440066 | C/T | *MAU2* | 0.018 | 0.0024 | 1.61e-13 | 54 | 0.005 | 0.0224 | 0.805 |
| 19 | rs12459249 ^*^ | 41339896 | T/C | *CTC-490E21.12:*  *CTC-490E21.10* | -0.015 | 0.0024 | 7.82e-10 | 38 | 0.019 | 0.0223 | 0.396 |
| 20 | rs6033239 | 11845930 | G/T | *LINC00687* | 0.014 | 0.0024 | 3.91e-09 | 35 | 0.023 | 0.0219 | 0.289 |
| 20 | rs6129077 | 59791342 | C/G | *CDH4* | -0.014 | 0.0024 | 2.79e-08 | 31 | -0.031 | 0.0225 | 0.173 |
| 20 | rs73147887 | 62890294 | C/G | *PCMTD2* | -0.024 | 0.0028 | 2.97e-18 | 76 | -0.021 | 0.0258 | 0.417 |
| **Cheese** | | | | | | | | | | | |
| 1 | rs7513705 | 93666349 | T/C | *CCDC18* | -0.024 | 0.0040 | 2.05e-09 | 36 | 0.012 | 0.0221 | 0.593 |
| 1 | rs6685323 | 154295592 | C/T | *AQP10* | 0.023 | 0.0042 | 2.35e-08 | 31 | 0.036 | 0.0229 | 0.111 |
| 2 | rs72790304 | 24108557 | G/A | *ATAD2B* | 0.032 | 0.0051 | 3.69e-10 | 39 | 0.049 | 0.0279 | 0.080 |
| 2 | rs543059375 | 43910835 | C/A | *PLEKHH2* | 0.528 | 0.0965 | 4.53e-08 | 30 | 0.970 | 0.6113 | 0.113 |
| 2 | rs549814 | 45153508 | C/T | *RP11-89K21.1* | -0.044 | 0.0041 | 1.82e-26 | 113 | -0.014 | 0.0226 | 0.542 |
| 2 | rs12475594 | 58433375 | A/G | *FANCL* | -0.030 | 0.0051 | 2.09e-09 | 36 | -0.008 | 0.0277 | 0.770 |
| 2 | rs12472445 | 161309867 | G/C | *RBMS1* | 0.025 | 0.0044 | 3.17e-08 | 31 | 0.035 | 0.0241 | 0.150 |
| 2 | rs1014444 | 162868858 | A/G | *DPP4* | -0.024 | 0.0041 | 5.27e-09 | 34 | 0.000 | 0.0226 | 0.984 |
| 2 | rs1514755 | 166299635 | A/G | *CSRNP3* | -0.025 | 0.0045 | 4.65e-08 | 30 | 0.010 | 0.0247 | 0.694 |
| 3 | rs191087010 | 36713977 | T/C | *RN7SKP227* | 0.112 | 0.0205 | 4.31e-08 | 30 | -0.096 | 0.1273 | 0.453 |
| 3 | 3:49800212 | 49800212 | CT/C | *IP6K1* | 0.030 | 0.0039 | 1.25e-14 | 59 | 0.040 | 0.0209 | 0.054 |
| 3 | rs62245792 | 68410652 | T/A | *FAM19A1* | 0.031 | 0.0055 | 1.05e-08 | 33 | 0.026 | 0.0300 | 0.378 |
| 3 | rs2271054 | 107763040 | G/A | *CD47* | 0.028 | 0.0051 | 3.19e-08 | 31 | 0.019 | 0.0284 | 0.507 |
| 3 | rs2054710 | 161090616 | C/T | *SPTSSB* | -0.023 | 0.0042 | 3.14e-08 | 31 | 0.037 | 0.0229 | 0.105 |
| 3 | rs79184944 | 161549397 | T/A | *RP11-774I5.1* | -0.034 | 0.0057 | 1.33e-09 | 37 | 0.030 | 0.0311 | 0.335 |
| 4 | rs113591949 | 17851945 | A/G | *LCORL* | 0.038 | 0.0059 | 5.48e-11 | 43 | -0.007 | 0.0324 | 0.838 |
| 4 | rs10938397 ^†^ | 45182527 | A/G | *RP11-362I1.1* | 0.024 | 0.0039 | 3.94e-10 | 39 | 0.064 | 0.0214 | 0.003 |
| 4 | rs11425887 | 80938689 | C/CA | *ANTXR2* | -0.026 | 0.0044 | 2.16e-09 | 36 | 0.033 | 0.0239 | 0.173 |
| 4 | rs13107325 | 103188709 | C/T | *SLC39A8* | 0.047 | 0.0074 | 2.48e-10 | 40 | 0.015 | 0.0403 | 0.716 |
| 4 | rs4692708 | 170228542 | A/C | *SH3RF1* | -0.026 | 0.0045 | 5.51e-09 | 34 | -0.007 | 0.0244 | 0.780 |
| 5 | rs2202268 | 62966917 | T/G | *RP11-158J3.2* | 0.023 | 0.0040 | 7.59e-09 | 33 | 0.008 | 0.0216 | 0.709 |
| 5 | rs12522093 | 153603147 | T/A | *GALNT10* | 0.022 | 0.0039 | 3.52e-08 | 30 | -0.001 | 0.0213 | 0.951 |
| 6 | rs975303 | 19028788 | A/G | *RP11-254A17.1* | -0.031 | 0.0050 | 4.96e-10 | 39 | -0.001 | 0.0275 | 0.968 |
| 6 | rs75397441 ^†^ | 26022392 | C/T | *HIST1H4A* | -0.038 | 0.0067 | 1.65e-08 | 32 | -0.074 | 0.0368 | 0.043 |
| 6 | rs62412526 | 56553969 | G/T | *DST* | 0.026 | 0.0046 | 9.37e-09 | 33 | -0.041 | 0.0250 | 0.105 |
| 7 | 7:2206114 | 2206114 | TA/T | *MAD1L1* | 0.030 | 0.0046 | 1.15e-10 | 42 | 0.014 | 0.0253 | 0.588 |
| 7 | 7:18152033 | 18152033 | CA/C | *HDAC9* | 0.028 | 0.0049 | 9.95e-09 | 33 | -0.034 | 0.0271 | 0.208 |
| 7 | rs12672200 | 115461436 | G/A | *Y_RNA* | 0.024 | 0.0041 | 8.28e-09 | 33 | 0.001 | 0.0226 | 0.952 |
| 7 | rs56182580 | 132691858 | T/C | *CHCHD3* | 0.026 | 0.0043 | 2.26e-09 | 36 | 0.036 | 0.0235 | 0.121 |
| 7 | rs60198071 | 140144929 | A/G | *MKRN1* | -0.025 | 0.0044 | 1.60e-08 | 32 | -0.040 | 0.0240 | 0.099 |
| 8 | rs7012814 ^*^ | 9173358 | G/A | *RP11-115J16.1* | 0.031 | 0.0039 | 2.98e-15 | 62 | -0.007 | 0.0210 | 0.734 |
| 8 | rs77883185 | 85620323 | C/T | *RALYL* | 0.048 | 0.0087 | 2.72e-08 | 31 | -0.049 | 0.0484 | 0.312 |
| 9 | rs7047365 ^†^ | 124630333 | T/C | *TTLL11* | -0.021 | 0.0039 | 3.71e-08 | 30 | 0.047 | 0.0211 | 0.025 |
| 11 | 11:43623050 | 43623050 | CTTTT/C | *HSD17B12* | -0.023 | 0.0040 | 4.29e-09 | 34 | 0.009 | 0.0211 | 0.676 |
| 11 | rs17854357 | 65601560 | G/C | *CFL1:SNX32* | 0.031 | 0.0052 | 1.80e-09 | 36 | 0.014 | 0.0283 | 0.612 |
| 11 | 11:77297159 | 77297159 | CTGAG/C | *CLNS1A* | 0.046 | 0.0072 | 2.48e-10 | 40 | 0.025 | 0.0408 | 0.545 |
| 11 | rs11604424 | 116651115 | C/T | *ZNF259* | -0.027 | 0.0048 | 2.93e-08 | 31 | 0.006 | 0.0263 | 0.806 |
| 12 | rs4963739 | 24191827 | T/C | *SOX5* | 0.021 | 0.0039 | 3.64e-08 | 30 | 0.000 | 0.0211 | 0.998 |
| 12 | rs112890481 | 49859149 | C/CT | *SPATS2* | -0.030 | 0.0050 | 3.08e-09 | 35 | 0.016 | 0.0277 | 0.551 |
| 12 | rs1024853 | 107291383 | C/G | *EEF1B2P4* | 0.022 | 0.0039 | 1.40e-08 | 32 | 0.007 | 0.0211 | 0.726 |
| 12 | rs61953351 | 121456616 | G/T | *OASL* | -0.025 | 0.0045 | 3.66e-08 | 30 | 0.005 | 0.0244 | 0.846 |
| 12 | rs7299943 | 123593485 | T/A | *PITPNM2* | -0.027 | 0.0049 | 1.85e-08 | 32 | 0.036 | 0.0266 | 0.178 |
| 13 | 13:58354282 | 58354282 | TA/T | *PCDH17* | -0.030 | 0.0044 | 7.84e-12 | 47 | 0.013 | 0.0232 | 0.572 |
| 13 | rs34190997 | 59350679 | G/GTA | *DNAJA1P1* | -0.035 | 0.0061 | 1.20e-08 | 32 | 0.057 | 0.0345 | 0.098 |
| 13 | rs4886168 | 60073056 | T/C | *RNU7-88P* | 0.029 | 0.0053 | 3.65e-08 | 30 | 0.003 | 0.0290 | 0.910 |
| 13 | rs116066016 | 66293017 | A/C | *HNRNPA3P5* | -0.640 | 0.1160 | 3.44e-08 | 30 | 0.315 | 0.6521 | 0.629 |
| 14 | rs10143659 | 30145432 | A/T | *PRKD1* | 0.023 | 0.0039 | 6.74e-09 | 34 | -0.020 | 0.0214 | 0.345 |
| 15 | rs1473781 | 41818917 | G/A | *RPAP1* | 0.024 | 0.0041 | 4.36e-09 | 34 | -0.013 | 0.0223 | 0.558 |
| 16 | rs76632611 | 7742888 | C/T | *RBFOX1* | -0.031 | 0.0057 | 4.45e-08 | 30 | 0.005 | 0.0319 | 0.869 |
| 16 | rs276950 | 86243437 | T/C | *LINC01082* | 0.030 | 0.0054 | 4.17e-08 | 30 | -0.014 | 0.0297 | 0.630 |
| 17 | rs684214 ^†^ | 40696915 | C/T | *RP11-400F19.8* | 0.027 | 0.0043 | 5.53e-10 | 38 | -0.055 | 0.0235 | 0.019 |
| 17 | rs79475968 | 44326895 | T/C | *RP11-259G18.2* | 0.034 | 0.0050 | 1.24e-11 | 46 | 0.013 | 0.0275 | 0.643 |
| 17 | rs919109 | 46675977 | G/C | *HOXB3:HOXB-AS3:HOXB6* | -0.034 | 0.0056 | 8.94e-10 | 38 | 0.003 | 0.0306 | 0.929 |
| 17 | 17:62009101 | 62009101 | CTT/C | *CD79B* | -0.030 | 0.0044 | 1.45e-11 | 46 | -0.008 | 0.0243 | 0.752 |
| 18 | rs2960578 | 21143739 | T/G | *NPC1* | -0.027 | 0.0039 | 2.27e-12 | 49 | 0.010 | 0.0211 | 0.646 |
| 18 | rs7237482 | 44317079 | A/T | *ST8SIA5* | -0.024 | 0.0039 | 7.36e-10 | 38 | 0.001 | 0.0214 | 0.950 |
| 20 | rs6029941 | 35519475 | G/A | *TLDC2:SAMHD1* | 0.027 | 0.0039 | 2.89e-12 | 49 | -0.010 | 0.0212 | 0.626 |
| 22 | rs35821760 | 32398185 | A/T | *RN7SL305P* | 0.024 | 0.0044 | 2.91e-08 | 31 | -0.006 | 0.0240 | 0.793 |
| 22 | rs28741121 | 42025823 | G/A | *XRCC6* | -0.036 | 0.0052 | 5.71e-12 | 47 | -0.005 | 0.0284 | 0.864 |
| **Total fruits** | | | | | | | | | | | |
| 1 | rs144200595 | 17859305 | G/A | *ARHGEF10L* | -1.737 | 0.2810 | 6.37e-10 | 38 | -2.109 | 1.7543 | 0.229 |
| 1 | 1:26199000 | 26199000 | ATT/A | *PAQR7* | 0.023 | 0.0042 | 4.64e-08 | 30 | -0.023 | 0.0209 | 0.272 |
| 1 | rs1620977 | 72729142 | A/G | *NEGR1* | 0.041 | 0.0047 | 7.53e-19 | 79 | -0.002 | 0.0239 | 0.949 |
| 1 | rs61421373 | 204560440 | C/T | *RP11-430C7.4* | -0.028 | 0.0050 | 1.14e-08 | 33 | 0.012 | 0.0255 | 0.632 |
| 1 | rs112087954 | 205179021 | C/T | *DSTYK* | -2.206 | 0.3933 | 2.03e-08 | 31 | 0.680 | 3.1050 | 0.827 |
| 2 | rs10188334 | 653874 | C/T | *TMEM18* | 0.040 | 0.0055 | 3.02e-13 | 53 | 0.036 | 0.0284 | 0.202 |
| 2 | rs535987588 | 16794221 | G/A | *FAM49A* | -0.973 | 0.1334 | 2.99e-13 | 53 | 1.101 | 0.7743 | 0.155 |
| 2 | rs4953150 | 45157336 | C/T | *RP11-89K21.1* | 0.024 | 0.0044 | 4.71e-08 | 30 | -0.020 | 0.0223 | 0.377 |
| 2 | rs12477242 | 58452792 | C/G | *FANCL* | -0.026 | 0.0044 | 2.39e-09 | 36 | 0.001 | 0.0223 | 0.963 |
| 2 | rs6731782 | 60224329 | A/T | *RP11-444A22.1* | 0.026 | 0.0041 | 2.16e-10 | 40 | 0.021 | 0.0211 | 0.323 |
| 2 | rs2710641 | 63149265 | G/A | *EHBP1* | -0.024 | 0.0043 | 1.85e-08 | 32 | 0.041 | 0.0219 | 0.062 |
| 2 | rs576565334 | 102164766 | T/A | *AC092570.4* | -1.607 | 0.2556 | 3.22e-10 | 40 | 1.360 | 1.6034 | 0.396 |
| 2 | rs111915841 | 225467840 | G/C | *CUL3* | -0.025 | 0.0044 | 1.21e-08 | 32 | 0.014 | 0.0227 | 0.545 |
| 2 | rs568572432 | 232572795 | C/T | *PTMA* | -0.883 | 0.1617 | 4.72e-08 | 30 | 1.007 | 1.0390 | 0.333 |
| 3 | rs13069655 | 21025726 | G/C | *AC104441.1* | -0.023 | 0.0041 | 3.49e-08 | 30 | -0.017 | 0.0209 | 0.427 |
| 3 | rs10490869 | 35635145 | A/T | *ARPP21* | 0.031 | 0.0051 | 1.09e-09 | 37 | -0.009 | 0.0261 | 0.721 |
| 3 | rs12637791 | 85525323 | T/G | *CADM2* | 0.024 | 0.0043 | 3.02e-08 | 31 | -0.003 | 0.0221 | 0.894 |
| 3 | rs7647305 | 185834290 | T/C | *DGKG* | -0.028 | 0.0050 | 2.92e-08 | 31 | 0.003 | 0.0248 | 0.892 |
| 4 | rs547606538 | 7704769 | A/G | *SORCS2* | -0.272 | 0.0496 | 4.36e-08 | 30 | 0.333 | 0.2815 | 0.237 |
| 4 | rs357842 | 60004974 | A/G | *RP11-622J8.1* | 0.027 | 0.0044 | 1.09e-09 | 37 | -0.003 | 0.0223 | 0.891 |
| 4 | rs183649060 | 69697924 | A/T | *UGT2B10* | -1.095 | 0.1969 | 2.64e-08 | 31 | 1.065 | 1.6274 | 0.513 |
| 4 | rs140675951 | 108300048 | T/A | *RNU6-551P* | -1.175 | 0.2138 | 3.89e-08 | 30 | 0.939 | 1.1593 | 0.418 |
| 5 | rs6864095 | 60545515 | G/T | *CTC-436P18.3* | -0.025 | 0.0041 | 1.19e-09 | 37 | 0.007 | 0.0210 | 0.757 |
| 5 | rs114489117 | 92948485 | T/A | *FAM172A* | -0.038 | 0.0067 | 1.48e-08 | 32 | -0.039 | 0.0344 | 0.259 |
| 5 | rs10070258 | 93980014 | T/G | *ANKRD32* | 0.027 | 0.0045 | 1.35e-09 | 37 | 0.027 | 0.0229 | 0.233 |
| 6 | rs764511845 | 51215155 | TAAAAG  AGTAAA  AGAAAA  G/T | *RP3-437C15.2* | -0.040 | 0.0049 | 1.66e-16 | 68 | 0.015 | 0.0250 | 0.542 |
| 6 | rs149365686 | 98783306 | T/TTCTC | *RP11-436D23.1* | 0.028 | 0.0041 | 1.53e-11 | 45 | -0.005 | 0.0209 | 0.810 |
| 6 | rs561713124 | 104498086 | G/A | *NPM1P10* | -0.936 | 0.1653 | 1.48e-08 | 32 | 0.965 | 1.1207 | 0.389 |
| 6 | rs539376176 | 122169947 | A/C | *HMGB3P18* | -0.843 | 0.1451 | 6.18e-09 | 34 | -0.815 | 0.7895 | 0.302 |
| 6 | rs183000662 | 129167476 | C/T | *LAMA2* | -1.138 | 0.1753 | 8.34e-11 | 42 | 1.153 | 1.0659 | 0.279 |
| 6 | rs559782145 | 134001863 | C/G | *RP3-323P13.2* | -1.280 | 0.2056 | 4.79e-10 | 39 | 1.226 | 1.6802 | 0.465 |
| 7 | rs587612927 | 74035427 | C/T | *RP5-1186P10.2* | -0.856 | 0.1503 | 1.22e-08 | 32 | 1.072 | 0.8994 | 0.233 |
| 7 | rs572190897 | 99186455 | A/C | *GS1-259H13.10* | -1.656 | 0.2959 | 2.17e-08 | 31 | 0.892 | 2.3109 | 0.700 |
| 7 | rs34475839 | 133622287 | C/A | *EXOC4* | 0.032 | 0.0054 | 2.36e-09 | 36 | -0.047 | 0.0279 | 0.096 |
| 7 | rs6967154 | 143713124 | A/T | *OR6B1* | -0.059 | 0.0042 | 4.93e-44 | 194 | 0.017 | 0.0218 | 0.434 |
| 7 | rs2533200 | 153489074 | C/G | *DPP6* | 0.024 | 0.0041 | 8.08e-09 | 33 | 0.025 | 0.0209 | 0.226 |
| 8 | rs531625906 | 10197291 | T/TC | *MSRA* | 0.032 | 0.0049 | 8.42e-11 | 42 | -0.020 | 0.0254 | 0.427 |
| 8 | rs7005201 | 59901813 | G/T | *TOX* | -0.025 | 0.0045 | 2.89e-08 | 31 | 0.010 | 0.0227 | 0.649 |
| 9 | rs115540638 | 34191992 | A/T | *UBAP1* | 0.029 | 0.0050 | 5.31e-09 | 34 | -0.015 | 0.0256 | 0.566 |
| 9 | rs943617 | 114937852 | A/G | *SUSD1* | 0.028 | 0.0049 | 2.44e-08 | 31 | 0.019 | 0.0251 | 0.443 |
| 9 | rs144105946 | 119148887 | T/G | *PAPPA* | -1.641 | 0.2438 | 1.67e-11 | 45 | 0.305 | 1.8013 | 0.865 |
| 10 | rs10828266 | 22098701 | A/G | *DNAJC1* | -0.046 | 0.0046 | 9.45e-24 | 101 | 0.030 | 0.0231 | 0.198 |
| 10 | rs144373510 | 55166107 | A/G | *RNA5SP318* | -0.599 | 0.1072 | 2.32e-08 | 31 | 0.722 | 0.5774 | 0.211 |
| 10 | rs12245149 | 65321147 | C/A | *REEP3* | 0.023 | 0.0041 | 1.52e-08 | 32 | -0.001 | 0.0212 | 0.954 |
| 10 | 10:126684759 | 126684759 | GC/G | *CTBP2* | -0.041 | 0.0064 | 1.78e-10 | 41 | -0.026 | 0.0284 | 0.369 |
| 11 | rs10128597 | 8694830 | G/A | *TRIM66* | -0.028 | 0.0046 | 8.33e-10 | 38 | 0.003 | 0.0239 | 0.898 |
| 11 | rs3763874 | 9546587 | G/A | *ZNF143* | -0.027 | 0.0042 | 1.38e-10 | 41 | 0.008 | 0.0214 | 0.707 |
| 11 | rs61877052 | 10074294 | A/C | *SBF2* | 0.059 | 0.0106 | 2.15e-08 | 31 | 0.015 | 0.0541 | 0.776 |
| 11 | rs11032362 ^*^ | 33759092 | G/A | *CD59* | -0.041 | 0.0072 | 9.96e-09 | 33 | 0.009 | 0.0368 | 0.811 |
| 11 | rs569920322 | 72856001 | A/G | *FCHSD2* | -0.925 | 0.1648 | 1.96e-08 | 32 | 0.878 | 1.3342 | 0.511 |
| 11 | rs563891191 | 98324748 | C/T | *CTD-2342I9.1* | -1.339 | 0.2328 | 8.74e-09 | 33 | 1.401 | 3.0386 | 0.645 |
| 12 | rs546444490 | 18725611 | C/A | *PIK3C2G* | -0.903 | 0.1508 | 2.18e-09 | 36 | 1.260 | 0.9850 | 0.201 |
| 12 | 12:90604769 | 90604769 | GA/G | *RP11-753N8.1* | 0.027 | 0.0046 | 1.01e-08 | 33 | -0.007 | 0.0237 | 0.783 |
| 13 | rs573488836 | 88887863 | A/G | *RP11-545P6.2* | -1.358 | 0.2278 | 2.48e-09 | 36 | 0.993 | 1.4373 | 0.490 |
| 14 | rs34162196 | 22038125 | C/T | *OR10G3* | 0.059 | 0.0068 | 3.30e-18 | 76 | 0.009 | 0.0350 | 0.797 |
| 14 | 14:43982395 ^‡^ | 43982395 | TA/T | *KRT8P2* | -1.677 | 0.3028 | 3.08e-08 | 31 | NA | NA | NA |
| 14 | rs2370982 | 79890677 | C/T | *NRXN3* | -0.028 | 0.0050 | 4.64e-08 | 30 | 0.008 | 0.0260 | 0.762 |
| 15 | rs561549058 | 37047924 | G/A | *C15orf41* | -0.760 | 0.1335 | 1.24e-08 | 32 | -0.018 | 0.7321 | 0.980 |
| 15 | rs11073269 | 38120848 | T/A | *TMCO5A* | 0.023 | 0.0041 | 4.51e-08 | 30 | 0.005 | 0.0209 | 0.826 |
| 16 | rs140227131 | 5910535 | G/A | *RP11-420N3.2* | -0.625 | 0.1113 | 1.95e-08 | 32 | -0.121 | 0.5952 | 0.839 |
| 16 | rs7205037 ^†^ | 7624265 | G/C | *RBFOX1* | 0.023 | 0.0041 | 3.36e-08 | 30 | -0.059 | 0.0212 | 0.006 |
| 16 | 16:19269275 ^†^ | 19269275 | GT/G | *SYT17* | 0.024 | 0.0043 | 2.57e-08 | 31 | -0.058 | 0.0211 | 0.006 |
| 16 | rs572940299 | 25421313 | T/C | *CTA-249B10.1* | -1.334 | 0.2289 | 5.59e-09 | 34 | 1.185 | 2.5571 | 0.643 |
| 16 | rs112539866 | 30050078 | C/CA | *FAM57B* | -0.024 | 0.0042 | 1.05e-08 | 33 | -0.010 | 0.0210 | 0.634 |
| 16 | rs567194467 | 57467817 | G/T | *CIAPIN1* | -1.009 | 0.1719 | 4.28e-09 | 34 | 0.184 | 0.9346 | 0.844 |
| 16 | rs2967193 | 64288692 | T/C | *AC012322.1* | 0.025 | 0.0042 | 3.15e-09 | 35 | 0.029 | 0.0211 | 0.175 |
| 16 | rs67541953 | 73605538 | A/AT | *CTD-2009A10.1* | 0.025 | 0.0041 | 2.93e-09 | 35 | -0.012 | 0.0208 | 0.558 |
| 16 | rs531881647 | 77577429 | G/C | *RP11-571O6.1* | -1.288 | 0.2090 | 7.23e-10 | 38 | 0.859 | 1.3414 | 0.522 |
| 17 | rs529552269 | 25669886 | T/C | *RP11-173M1.4* | -0.785 | 0.1366 | 8.97e-09 | 33 | 0.141 | 0.8555 | 0.869 |
| 18 | rs34156224 | 24588876 | G/GT | *AQP4-AS1:CHST9* | 0.023 | 0.0042 | 4.69e-08 | 30 | -0.024 | 0.0215 | 0.264 |
| 18 | rs949017 | 44814404 | A/G | *CTD-2130O13.1* | -0.029 | 0.0042 | 5.57e-12 | 47 | 0.003 | 0.0208 | 0.893 |
| 18 | rs17773329 | 57944239 | A/G | *RP11-396N11.1* | -0.037 | 0.0045 | 1.70e-16 | 68 | 0.009 | 0.0228 | 0.689 |
| 18 | rs527897590 | 65038135 | T/G | *RP11-563H6.1* | -1.032 | 0.1694 | 1.10e-09 | 37 | 0.952 | 1.1009 | 0.387 |
| 19 | rs555698994 | 3068897 | A/T | *AES* | -1.168 | 0.1881 | 5.33e-10 | 39 | 1.096 | 1.3713 | 0.424 |
| 19 | rs429358 ^*^ | 45411941 | T/C | *APOE* | -0.048 | 0.0057 | 1.27e-17 | 73 | -0.051 | 0.0292 | 0.078 |
| 19 | rs2302593 | 46196634 | C/G | *QPCTL* | -0.026 | 0.0041 | 5.61e-10 | 38 | 0.003 | 0.0211 | 0.899 |
| 19 | rs8103840 ^*,†^ | 49254955 | C/T | *FUT1* | 0.023 | 0.0042 | 3.04e-08 | 31 | 0.042 | 0.0209 | 0.046 |
| 20 | rs78580419 | 42107712 | C/T | *RNU6-1251P* | -1.494 | 0.2574 | 6.58e-09 | 34 | 0.997 | 1.6686 | 0.550 |
| 20 | rs2425835 | 44901836 | A/G | *CDH22* | -0.024 | 0.0041 | 1.04e-08 | 33 | 0.023 | 0.0212 | 0.282 |
| 20 | rs549171695 | 52300532 | C/T | *RNU7-14P* | -1.075 | 0.1925 | 2.33e-08 | 31 | 1.245 | 1.7782 | 0.484 |
| 22 | rs6006228 | 30112070 | C/G | *RP1-76B20.11:RP1-76B20.12* | 0.023 | 0.0042 | 1.69e-08 | 32 | -0.024 | 0.0214 | 0.259 |
| **Total vegetables** | | | | | | | | | | | |
| 1 | rs531494375 | 11461163 | C/T | *RP11-149P14.1* | -1.828 | 0.3015 | 1.34e-09 | 37 | 1.133 | 1.2231 | 0.354 |
| 1 | rs372842590 | 91241265 | G/A | *RP4-665J23.1* | -1.268 | 0.2265 | 2.14e-08 | 31 | 1.065 | 0.7216 | 0.140 |
| 1 | 1:153761809 | 153761809 | AT/A | *Y_RNA* | 0.050 | 0.0069 | 5.62e-13 | 52 | 0.008 | 0.0207 | 0.696 |
| 2 | rs12714415 | 651430 | T/C | *TMEM18* | 0.062 | 0.0093 | 3.42e-11 | 44 | 0.030 | 0.0297 | 0.310 |
| 2 | rs548801702 | 4646677 | G/A | *AC022311.1* | -1.598 | 0.2862 | 2.35e-08 | 31 | -0.261 | 1.1680 | 0.823 |
| 2 | rs564723867 | 20714286 | C/T | *AC012065.4* | -1.234 | 0.2221 | 2.78e-08 | 31 | 0.501 | 0.7353 | 0.495 |
| 2 | rs1963398 | 79706878 | A/G | *CTNNA2* | 0.045 | 0.0072 | 5.51e-10 | 38 | -0.006 | 0.0225 | 0.801 |
| 2 | rs552320005 | 134226620 | C/A | *NCKAP5* | -1.048 | 0.1822 | 8.86e-09 | 33 | -1.086 | 0.7311 | 0.137 |
| 3 | rs570287640 | 7801663 | G/T | *GRM7* | -4.922 | 0.7238 | 1.05e-11 | 46 | 0.300 | 3.8124 | 0.937 |
| 3 | rs7619139 | 25110415 | T/A | *AC133680.1* | -0.056 | 0.0069 | 3.72e-16 | 66 | -0.008 | 0.0212 | 0.696 |
| 3 | rs140084370 | 109434812 | C/G | *RP11-457K10.1* | -1.984 | 0.3445 | 8.37e-09 | 33 | 0.912 | 1.3710 | 0.506 |
| 3 | rs150482236 | 116476055 | C/T | *LSAMP* | -1.372 | 0.2378 | 8.02e-09 | 33 | -1.191 | 0.8766 | 0.174 |
| 3 | rs532503391 | 117675492 | G/A | *LSAMP:RP11-384F7.2* | -1.196 | 0.2129 | 1.94e-08 | 32 | -0.017 | 0.7589 | 0.982 |
| 3 | rs527623148 | 122761126 | T/C | *SEMA5B* | -1.557 | 0.2380 | 6.08e-11 | 43 | 1.097 | 1.1810 | 0.353 |
| 3 | rs558817978 | 148472234 | G/C | *AGTR1* | -1.739 | 0.2689 | 1.01e-10 | 42 | 1.176 | 0.9257 | 0.204 |
| 3 | rs9822804 | 186159741 | G/T | *RP11-78H24.1* | -2.832 | 0.4540 | 4.44e-10 | 39 | 0.844 | 1.6036 | 0.598 |
| 4 | rs13115614 ^†^ | 136488540 | A/G | *TARS2P1* | -0.047 | 0.0083 | 1.42e-08 | 32 | 0.054 | 0.0260 | 0.037 |
| 5 | rs562634338 | 4691055 | A/C | *RP11-445O3.2* | -1.236 | 0.2107 | 4.38e-09 | 34 | 0.920 | 0.7497 | 0.220 |
| 7 | rs139549768 | 54932540 | A/T | *SNORA73* | -3.783 | 0.4597 | 1.89e-16 | 68 | 1.127 | 1.7340 | 0.516 |
| 7 | rs145929636 | 57391143 | C/G | *MIR3147* | -4.085 | 0.5178 | 3.04e-15 | 62 | 1.218 | 2.0238 | 0.547 |
| 7 | rs140838198 | 64802985 | C/G | *RSL24D1P3* | -2.379 | 0.4108 | 7.01e-09 | 34 | 1.142 | 1.4324 | 0.425 |
| 7 | rs139017236 | 67258155 | T/C | *RP11-421N10.1* | -2.555 | 0.3999 | 1.67e-10 | 41 | -1.019 | 1.4448 | 0.480 |
| 7 | rs541682859 | 138591620 | T/C | *KIAA1549* | -1.405 | 0.2370 | 3.07e-09 | 35 | 1.052 | 0.9562 | 0.271 |
| 8 | rs1879957 | 8544808 | T/C | *CLDN23* | 0.038 | 0.0068 | 1.76e-08 | 32 | -0.009 | 0.0211 | 0.660 |
| 8 | rs10101292 | 10856474 | T/C | *XKR6* | 0.041 | 0.0069 | 3.49e-09 | 35 | -0.016 | 0.0213 | 0.462 |
| 8 | rs547244654 | 37341987 | T/C | *RP11-150O12.1* | -1.683 | 0.2991 | 1.85e-08 | 32 | -0.803 | 1.2385 | 0.517 |
| 8 | rs190344992 | 63934378 | G/A | *GGH* | -1.962 | 0.3459 | 1.41e-08 | 32 | 0.869 | 2.0751 | 0.675 |
| 8 | 8:83459367 | 83459367 | GTTTC/  G | *RP11-653B10.1* | -1.970 | 0.2872 | 6.96e-12 | 47 | 0.030 | 0.9573 | 0.975 |
| 9 | rs561147080 | 15400432 | G/C | *SNAPC3* | -1.582 | 0.2628 | 1.76e-09 | 36 | 0.945 | 1.3175 | 0.473 |
| 9 | 9:23474196 | 23474196 | CACA/C | *SUMO2P2* | -2.718 | 0.4869 | 2.38e-08 | 31 | 0.804 | 1.8994 | 0.672 |
| 9 | rs577213322 | 120405271 | G/GA | *RP11-500B12.1* | -2.345 | 0.3675 | 1.76e-10 | 41 | 1.233 | 2.8150 | 0.661 |
| 9 | rs1411350 | 128670179 | A/G | *PBX3* | 0.045 | 0.0072 | 4.11e-10 | 39 | -0.014 | 0.0223 | 0.530 |
| 10 | rs34646324 | 22255363 | A/AT | *DNAJC1* | -0.046 | 0.0076 | 9.13e-10 | 38 | 0.033 | 0.0217 | 0.133 |
| 10 | rs189761289 | 45193614 | G/A | *RP11-733D4.1* | -2.209 | 0.3168 | 3.13e-12 | 49 | 0.969 | 1.1389 | 0.395 |
| 11 | rs73446263 | 37271445 | T/C | *SNORA31* | -1.270 | 0.2288 | 2.81e-08 | 31 | 0.586 | 0.7332 | 0.424 |
| 11 | rs149255293 | 106270546 | G/A | *RP11-680E19.2* | -1.531 | 0.2610 | 4.50e-09 | 34 | 1.005 | 0.8364 | 0.230 |
| 11 | rs567235405 | 132903412 | A/C | *OPCML* | -2.445 | 0.4323 | 1.55e-08 | 32 | 0.939 | 1.7960 | 0.601 |
| 12 | rs12821585 | 109886361 | T/C | *MYO1H* | 0.067 | 0.0111 | 2.21e-09 | 36 | -0.019 | 0.0348 | 0.577 |
| 13 | rs142801749 | 59469245 | A/AAAG | *HMGN2P39* | 0.044 | 0.0073 | 2.64e-09 | 35 | -0.016 | 0.0228 | 0.478 |
| 15 | rs567612175 | 35780145 | A/T | *DPH6* | -1.870 | 0.2771 | 1.49e-11 | 46 | 0.129 | 1.0107 | 0.899 |
| 15 | rs181495534 | 54039101 | A/T | *WDR72* | -3.338 | 0.4483 | 9.60e-14 | 55 | -1.719 | 1.7111 | 0.315 |
| 15 | rs144175081 | 69808671 | G/A | *RP11-279F6.1* | -1.061 | 0.1945 | 4.89e-08 | 30 | -0.167 | 0.6160 | 0.787 |
| 16 | rs112653268 ^†^ | 68115810 | A/G | *RP11-67A1.2* | -2.869 | 0.4046 | 1.32e-12 | 50 | -4.002 | 1.8068 | 0.027 |
| 17 | rs543035465 | 10160118 | T/C | *RPS27AP1* | -1.590 | 0.2872 | 3.06e-08 | 31 | 1.267 | 1.3584 | 0.351 |
| 18 | rs376756052 | 12226371 | C/T | *RP11-64C12.3* | -2.678 | 0.4739 | 1.59e-08 | 32 | 0.952 | 1.9258 | 0.621 |
| 18 | rs145430506 | 63572933 | T/C | *RP11-389J22.3* | -2.135 | 0.3662 | 5.54e-09 | 34 | -0.330 | 1.2136 | 0.786 |
| 18 | rs111538418 | 77498088 | T/C | *CTDP1* | -2.322 | 0.3793 | 9.21e-10 | 37 | 0.805 | 1.3863 | 0.561 |
| 21 | rs574416335 | 33908541 | C/T | *SNORA33* | -0.978 | 0.1760 | 2.71e-08 | 31 | -0.043 | 0.5951 | 0.943 |
| 22 | rs1023469 | 42759341 | T/C | *Z83851.1* | 0.038 | 0.0070 | 4.63e-08 | 30 | 0.018 | 0.0216 | 0.416 |
| 22 | rs572729180 | 46670726 | T/G | *TTC38* | -1.962 | 0.3413 | 9.00e-09 | 33 | -0.852 | 1.3898 | 0.540 |
| **Coffee** | | | | | | | | | | | |
| 1 | rs10305752 | 150783239 | A/AG | *ARNT* | 0.083 | 0.0147 | 1.84e-08 | 32 | -0.124 | 0.0708 | 0.081 |
| 1 | rs574367 ^†^ | 177873210 | G/T | *SEC16B* | -0.031 | 0.0054 | 1.35e-08 | 32 | -0.074 | 0.0258 | 0.004 |
| 2 | rs71415991 | 630089 | A/ATCT  ATAATC  TATCTC  TATG | *TMEM18* | -0.051 | 0.0059 | 5.50e-18 | 75 | -0.017 | 0.0270 | 0.537 |
| 2 | 2:27748992 ^*^ | 27748992 | AT/A | *GCKR* | -0.040 | 0.0045 | 2.75e-18 | 76 | -0.036 | 0.0212 | 0.089 |
| 2 | rs12989746 | 49368391 | G/T | *FSHR* | -0.028 | 0.0051 | 2.83e-08 | 31 | 0.005 | 0.0244 | 0.828 |
| 3 | rs71326934 | 50536383 | C/G | *CACNA2D2* | 0.038 | 0.0065 | 5.86e-09 | 34 | -0.016 | 0.0313 | 0.616 |
| 4 | rs1263412 | 2858005 | A/G | *ADD1* | 0.030 | 0.0048 | 3.58e-10 | 39 | -0.006 | 0.0229 | 0.808 |
| 4 | rs140590745 | 23930409 | A/C | *PPARGC1A* | 0.043 | 0.0078 | 2.05e-08 | 31 | 0.074 | 0.0381 | 0.053 |
| 4 | rs2199936 ^*^ | 89045331 | A/G | *ABCG2* | -0.053 | 0.0069 | 2.05e-14 | 58 | -0.020 | 0.0329 | 0.536 |
| 5 | rs12519880 | 7391434 | C/A | *ADCY2* | 0.034 | 0.0049 | 2.12e-12 | 49 | -0.007 | 0.0234 | 0.759 |
| 5 | rs304132 | 88215594 | A/G | *MEF2C-AS1* | 0.026 | 0.0044 | 6.69e-09 | 34 | -0.032 | 0.0214 | 0.133 |
| 6 | rs2465037 | 51179260 | C/A | *RP3-437C15.2* | 0.032 | 0.0046 | 7.68e-12 | 47 | 0.013 | 0.0223 | 0.574 |
| 6 | rs139797380 | 137244957 | C/G | *SLC35D3* | 0.152 | 0.0256 | 2.78e-09 | 35 | -0.077 | 0.1354 | 0.569 |
| 7 | rs4410790 ^*^ | 17284577 | T/C | *AC003075.4* | -0.122 | 0.0046 | 8.90e-157 | 712 | -0.015 | 0.0220 | 0.503 |
| 7 | rs2067150 | 70051412 | A/AGTA  TAATTA  TGATTA  G | *AUTS2* | 0.033 | 0.0059 | 2.42e-08 | 31 | 0.021 | 0.0289 | 0.466 |
| 7 | rs34060476 | 73037956 | A/G | *MLXIPL* | -0.061 | 0.0065 | 2.67e-21 | 90 | -0.017 | 0.0311 | 0.591 |
| 7 | rs1057868 | 75615006 | C/T | *POR* | -0.062 | 0.0049 | 3.27e-37 | 162 | 0.001 | 0.0233 | 0.952 |
| 8 | rs13271359 | 109114426 | C/T | *RSPO2* | 0.028 | 0.0050 | 1.94e-08 | 32 | 0.011 | 0.0242 | 0.647 |
| 9 | rs1014307 | 16053793 | C/T | *CCDC171* | 0.025 | 0.0045 | 1.45e-08 | 32 | -0.035 | 0.0210 | 0.090 |
| 11 | rs597045 | 56272114 | A/T | *OR5M7P* | 0.026 | 0.0048 | 4.45e-08 | 30 | -0.002 | 0.0231 | 0.939 |
| 11 | rs181359370 | 84774265 | T/C | *DLG2* | -0.416 | 0.0701 | 3.07e-09 | 35 | -0.355 | 0.4201 | 0.398 |
| 12 | 12:11271915 ^*^ | 11271915 | CA/C | *PRR4:TAS2R14* | 0.042 | 0.0061 | 4.89e-12 | 48 | 0.004 | 0.0252 | 0.877 |
| 12 | rs11060255 | 122677724 | G/A | *LRRC43* | -0.024 | 0.0044 | 4.13e-08 | 30 | 0.006 | 0.0208 | 0.783 |
| 15 | rs2017998 | 74721905 | G/C | *SEMA7A* | -0.066 | 0.0045 | 3.57e-48 | 213 | -0.015 | 0.0216 | 0.480 |
| 15 | rs2682909 | 77880927 | G/C | *RP11-307C19.2* | 0.025 | 0.0046 | 3.05e-08 | 31 | 0.004 | 0.0220 | 0.840 |
| 15 | rs2521501 | 91437388 | A/T | *FES* | 0.030 | 0.0047 | 3.96e-10 | 39 | 0.020 | 0.0227 | 0.376 |
| 16 | rs28562191 | 53799303 | C/T | *FTO* | -0.036 | 0.0045 | 1.47e-15 | 64 | -0.024 | 0.0214 | 0.263 |
| 17 | rs2905855 | 46081915 | G/A | *RP11-6N17.10* | 0.027 | 0.0044 | 1.24e-09 | 37 | 0.008 | 0.0212 | 0.711 |
| 17 | rs57918684 | 60150383 | G/A | *MED13* | -0.035 | 0.0061 | 1.07e-08 | 33 | -0.041 | 0.0294 | 0.159 |
| 18 | rs476828 | 57852587 | T/C | *RP11-795H16.2* | -0.048 | 0.0052 | 8.93e-21 | 87 | 0.024 | 0.0248 | 0.342 |
| 19 | rs12459249 ^*^ | 41339896 | T/C | *CTC-490E21.12:CTC-490E21.10* | -0.035 | 0.0047 | 1.12e-13 | 55 | 0.019 | 0.0223 | 0.396 |
| 20 | rs6062362 | 62911229 | A/G | *PCMTD2* | -0.034 | 0.0045 | 4.39e-14 | 57 | 0.003 | 0.0212 | 0.881 |
| 22 | rs181251778 | 24901968 | A/G | *UPB1* | 0.126 | 0.0188 | 2.14e-11 | 45 | 0.088 | 0.0895 | 0.328 |
| **Tea** | | | | | | | | | | | |
| 1 | rs201406724 ^*^ | 150781915 | T/TA | *ARNT* | -0.039 | 0.0060 | 5.48e-11 | 43 | 0.026 | 0.0222 | 0.249 |
| 1 | 1:154608308 | 154608308 | TGACTA  AATTCT  GTCTGT  CCTTTG  /T | *ADAR* | -0.037 | 0.0068 | 3.89e-08 | 30 | 0.024 | 0.0254 | 0.347 |
| 1 | rs146180628 | 174413325 | G/GT | *RABGAP1L* | 0.064 | 0.0082 | 7.81e-15 | 60 | 0.037 | 0.0310 | 0.234 |
| 1 | rs2813703 | 216913261 | G/A | *ESRRG* | -0.033 | 0.0057 | 1.09e-08 | 33 | -0.015 | 0.0214 | 0.486 |
| 2 | rs114278367 | 47958631 | A/G | *MSH6:AC006509.7* | 0.104 | 0.0182 | 1.30e-08 | 32 | -0.134 | 0.0690 | 0.053 |
| 2 | rs725452 | 58511300 | A/G | *AC007250.4* | 0.039 | 0.0069 | 2.12e-08 | 31 | -0.007 | 0.0260 | 0.803 |
| 3 | 3:42244986 ^†^ | 42244986 | CTTT/C | *TRAK1* | -0.035 | 0.0064 | 4.47e-08 | 30 | 0.047 | 0.0232 | 0.044 |
| 3 | rs6771054 | 89489529 | T/C | *EPHA3* | -0.035 | 0.0058 | 7.68e-10 | 38 | -0.021 | 0.0215 | 0.331 |
| 4 | rs28863537 | 67932876 | T/A | *RNU6-699P* | -0.034 | 0.0058 | 2.30e-09 | 36 | -0.011 | 0.0215 | 0.619 |
| 4 | rs2199936 ^*^ | 89045331 | A/G | *ABCG2* | -0.070 | 0.0089 | 2.36e-15 | 63 | -0.020 | 0.0329 | 0.536 |
| 5 | rs76823566 | 60413507 | T/TA | *NDUFAF2* | -0.032 | 0.0057 | 2.01e-08 | 31 | 0.010 | 0.0213 | 0.631 |
| 5 | rs192084998 | 152077481 | G/A | *AC091969.1* | 0.041 | 0.0062 | 5.32e-11 | 43 | 0.003 | 0.0232 | 0.892 |
| 6 | rs2465018 ^*^ | 51241140 | G/A | *RP3-437C15.2* | -0.055 | 0.0067 | 2.05e-16 | 68 | 0.007 | 0.0253 | 0.786 |
| 6 | rs139797380 | 137244957 | C/G | *SLC35D3* | 0.234 | 0.0329 | 1.13e-12 | 51 | -0.077 | 0.1354 | 0.569 |
| 7 | rs1010123 | 13318566 | T/A | *AC011288.2* | -0.041 | 0.0067 | 9.07e-10 | 38 | 0.028 | 0.0251 | 0.265 |
| 7 | rs4410790 ^*^ | 17284577 | T/C | *AC003075.4* | -0.115 | 0.0059 | 9.38E-86 | 385 | -0.015 | 0.0220 | 0.503 |
| 7 | rs6462899 | 39296489 | T/A | *POU6F2* | -0.032 | 0.0058 | 2.87e-08 | 31 | -0.005 | 0.0216 | 0.811 |
| 7 | rs17685 ^*^ | 75616105 | G/A | *POR* | -0.066 | 0.0063 | 3.96e-26 | 112 | 0.006 | 0.0235 | 0.792 |
| 7 | rs4726481 ^*^ | 141668403 | G/T | *MGAM* | -0.037 | 0.0058 | 1.72e-10 | 41 | 0.003 | 0.0215 | 0.885 |
| 8 | rs7012814 ^*^ | 9173358 | G/A | *RP11-115J16.1* | 0.032 | 0.0057 | 1.29e-08 | 32 | -0.007 | 0.0210 | 0.734 |
| 8 | rs80318442 | 34285545 | T/G | *RP1-84O15.2* | -0.075 | 0.0133 | 1.92e-08 | 32 | 0.008 | 0.0504 | 0.881 |
| 8 | 8:73433232 ^*^ | 73433232 | GGTA/G | *KCNB2* | -0.034 | 0.0062 | 3.35e-08 | 30 | -0.009 | 0.0227 | 0.697 |
| 9 | rs7852678 | 7031134 | A/G | *KDM4C* | -0.039 | 0.0070 | 1.88e-08 | 32 | 0.012 | 0.0265 | 0.645 |
| 10 | rs10752269 | 12692902 | G/A | *CAMK1D* | 0.033 | 0.0056 | 4.40e-09 | 34 | 0.009 | 0.0210 | 0.655 |
| 10 | rs10764990 | 129152608 | G/A | *DOCK1* | 0.032 | 0.0058 | 2.56e-08 | 31 | 0.013 | 0.0216 | 0.550 |
| 11 | rs11022752 | 13307622 | A/G | *ARNTL* | -0.037 | 0.0064 | 4.94e-09 | 34 | 0.013 | 0.0239 | 0.580 |
| 11 | rs10741694 | 16286183 | T/C | *SOX6* | -0.039 | 0.0058 | 3.68e-11 | 44 | -0.003 | 0.0216 | 0.877 |
| 12 | 12:11271915 ^*^ | 11271915 | CA/C | *PRR4:TAS2R14* | -0.057 | 0.0078 | 2.61e-13 | 53 | 0.004 | 0.0252 | 0.877 |
| 12 | rs11065898 | 111862575 | C/T | *SH2B3* | -0.040 | 0.0068 | 2.75e-09 | 35 | -0.023 | 0.0255 | 0.376 |
| 13 | rs7321386 | 89213194 | T/C | *LINC00433* | -0.033 | 0.0057 | 4.54e-09 | 34 | -0.018 | 0.0213 | 0.389 |
| 13 | rs753207 | 111528111 | T/C | *ANKRD10* | -0.031 | 0.0057 | 3.73e-08 | 30 | 0.023 | 0.0213 | 0.275 |
| 15 | rs12591786 ^*^ | 60902512 | C/T | *RP11-219B17.1:RORA* | 0.049 | 0.0078 | 5.04e-10 | 39 | 0.027 | 0.0297 | 0.369 |
| 15 | rs12909335 ^*^ | 75214789 | T/A | *COX5A* | -0.076 | 0.0057 | 2.31e-40 | 177 | 0.016 | 0.3902 | 0.967 |
| 16 | rs9937521 ^*^ | 53799296 | C/T | *FTO* | 0.038 | 0.0057 | 2.14e-11 | 45 | -0.024 | 0.0214 | 0.261 |
| 16 | rs512404 | 63031551 | G/T | *RP11-96H17.1* | -0.037 | 0.0068 | 3.77e-08 | 30 | 0.016 | 0.0256 | 0.537 |
| 19 | rs2074550 | 19387743 | A/C | *SUGP1* | -0.042 | 0.0060 | 3.61e-12 | 48 | 0.000 | 0.0224 | 0.991 |
| 20 | rs2273447 | 62900120 | A/T | *PCMTD2* | -0.051 | 0.0070 | 2.86e-13 | 53 | -0.013 | 0.0262 | 0.614 |
| 21 | rs4817506 | 34348569 | G/A | *AP000282.2* | -0.032 | 0.0057 | 1.51e-08 | 32 | 0.015 | 0.0209 | 0.463 |
| 22 | rs4820593 | 24887087 | A/T | *ADORA2A-AS1:UPB1* | -0.066 | 0.0057 | 5.28e-31 | 134 | 0.007 | 0.0211 | 0.723 |
| 22 | rs73424602 | 41461176 | C/T | *Y_RNA* | 0.041 | 0.0058 | 6.81e-13 | 52 | 0.007 | 0.0215 | 0.740 |
|  |  |  |  |  |  |  |  |  |  |  |  |
| **Alcohol** | | | | | | | | | | | |
| 1 | rs780569 | 4569436 | T/A | *RP1-37J18.1* | 0.034 | 0.006 | 1.31e-08 | 32 | -0.005 | 0.0216 | 0.813 |
| 1 | rs6678286 | 174169619 | A/G | *RABGAP1L* | 0.033 | 0.006 | 4.08e-08 | 30 | 0.001 | 0.0236 | 0.956 |
| 2 | rs66523860 | 23880823 | A/G | *KLHL29* | 0.039 | 0.007 | 6.48e-09 | 34 | 0.004 | 0.0261 | 0.878 |
| 2 | 2:27748992 ^*^ | 27748992 | AT/A | *GCKR* | -0.09 | 0.006 | 1.36e-55 | 247 | -0.036 | 0.0212 | 0.089 |
| 2 | rs494904 | 45141180 | T/C | *RP11-89K21.1* | -0.04 | 0.006 | 1.69e-11 | 45 | -0.015 | 0.0216 | 0.496 |
| 2 | rs13390019 | 97797680 | T/C | *ANKRD36* | 0.047 | 0.008 | 5.14e-09 | 34 | -0.027 | 0.0318 | 0.387 |
| 2 | rs6436555 ^†^ | 157488277 | A/C | *GPD2* | 0.03 | 0.005 | 2.97e-08 | 31 | 0.055 | 0.0209 | 0.009 |
| 2 | 2:161338926 | 161338926 | TAAATA AAC/T | *RBMS1* | 0.035 | 0.006 | 1.12e-08 | 33 | -0.008 | 0.0239 | 0.731 |
| 2 | rs5836601 | 178155252 | C/CTTT | *NFE2L2:AC074286.1* | -0.04 | 0.007 | 6.42e-09 | 34 | 0.042 | 0.0249 | 0.094 |
| 2 | rs10188314 | 215402926 | C/T | *AC107218.3:VWC2L* | -0.03 | 0.005 | 5.79e-09 | 34 | 0.004 | 0.0210 | 0.852 |
| 3 | rs2051214 | 38560303 | C/A | *EXOG* | 0.032 | 0.006 | 8.17e-09 | 33 | -0.014 | 0.0217 | 0.532 |
| 3 | 3:49959570 ^†^ | 49959570 | CA/C | *MON1A* | 0.038 | 0.005 | 6.18e-12 | 47 | 0.066 | 0.0208 | 0.002 |
| 3 | rs142623271 | 70676591 | A/ATGTT T | *COX6CP6* | 0.035 | 0.006 | 6.85e-09 | 34 | 0.012 | 0.0233 | 0.606 |
| 3 | rs704255 | 71669656 | G/A | *RP11-154H23.3* | -0.03 | 0.006 | 2.00e-09 | 36 | 0.012 | 0.0214 | 0.580 |
| 3 | rs9822731 | 85405501 | T/C | *CADM2* | -0.04 | 0.007 | 5.35e-10 | 39 | 0.025 | 0.0253 | 0.319 |
| 4 | rs2858088 | 3268710 | A/G | *MSANTD1* | -0.04 | 0.006 | 4.24e-11 | 43 | -0.013 | 0.0216 | 0.561 |
| 4 | rs11940694 ^*^ | 39414993 | A/G | *KLB* | -0.08 | 0.006 | 1.43e-42 | 187 | -0.014 | 0.0211 | 0.506 |
| 4 | rs4864919 | 55579484 | T/A | *KIT* | -0.04 | 0.006 | 1.75e-11 | 45 | 0.009 | 0.0208 | 0.650 |
| 4 | rs759387670 | 67965761 | AGATT/A | *RNU6-699P* | -0.04 | 0.006 | 2.37e-09 | 36 | 0.012 | 0.0228 | 0.611 |
| 4 | rs29001570 | 99994405 | T/C | *ADH5* | 0.517 | 0.036 | 1.85e-47 | 209 | -0.067 | 0.1531 | 0.664 |
| 4 | rs13135092 | 103198082 | A/G | *SLC39A8* | 0.079 | 0.01 | 1.78e-15 | 63 | -0.014 | 0.0388 | 0.716 |
| 5 | rs4916723 | 87854395 | A/C | *LINC00461* | 0.039 | 0.006 | 1.90e-12 | 50 | 0.033 | 0.0213 | 0.124 |
| 5 | rs12521723 | 132273808 | A/T | *AFF4* | 0.042 | 0.007 | 1.10e-08 | 33 | 0.048 | 0.0286 | 0.093 |
| 5 | rs141688667 | 145660413 | T/TTTTA | *RBM27* | -0.03 | 0.006 | 3.78e-08 | 30 | -0.040 | 0.0238 | 0.096 |
| 6 | rs9482094 | 98364895 | A/G | *RP11-436D23.1* | 0.051 | 0.006 | 6.39e-20 | 83 | -0.025 | 0.0219 | 0.250 |
| 7 | rs12701714 | 39326147 | G/A | *POU6F2* | 0.031 | 0.005 | 1.65e-08 | 32 | -0.002 | 0.0211 | 0.922 |
| 7 | rs62466318 | 73042085 | C/T | *MLXIPL* | -0.05 | 0.007 | 2.25e-13 | 54 | -0.020 | 0.0263 | 0.456 |
| 7 | rs4726481 ^*^ | 141668403 | G/T | *MGAM* | 0.033 | 0.006 | 2.91e-09 | 35 | 0.003 | 0.0215 | 0.885 |
| 8 | rs1566085 | 142624527 | G/T | *AC138647.1* | -0.04 | 0.005 | 4.71e-11 | 43 | 0.003 | 0.0209 | 0.901 |
| 8 | 8:143485680 | 143485680 | CTG/C | *RP13-467H17.1* | 0.04 | 0.007 | 1.81e-08 | 32 | 0.045 | 0.0271 | 0.099 |
| 11 | rs7124396 | 47787434 | T/G | *FNBP4* | 0.039 | 0.005 | 7.50e-13 | 51 | 0.000 | 0.0211 | 0.993 |
| 11 | rs4309187 | 113412443 | A/C | *DRD2* | -0.04 | 0.006 | 2.36e-09 | 36 | -0.005 | 0.0222 | 0.826 |
| 11 | rs748919 | 133783232 | T/C | *IGSF9B* | 0.038 | 0.007 | 1.77e-08 | 32 | -0.016 | 0.0262 | 0.535 |
| 12 | rs12367809 | 50256063 | C/T | *RP11-70F11.7* | 0.031 | 0.006 | 3.30e-08 | 31 | 0.002 | 0.0219 | 0.916 |
| 12 | rs12425616 | 54634666 | T/C | *CBX5* | -0.05 | 0.007 | 2.76e-12 | 49 | 0.002 | 0.0284 | 0.932 |
| 12 | rs36159461 | 56468832 | G/GA | *ERBB3* | 0.032 | 0.006 | 1.23e-08 | 32 | -0.018 | 0.0213 | 0.404 |
| 13 | rs7338471 | 49981064 | G/T | *CAB39L* | -0.04 | 0.006 | 1.07e-09 | 37 | 0.010 | 0.0223 | 0.660 |
| 14 | rs17698314 | 56846715 | C/A | *RP11-930O11.1* | -0.04 | 0.006 | 9.26e-09 | 33 | 0.012 | 0.0246 | 0.621 |
| 16 | rs2286975 | 11114006 | G/A | *CLEC16A* | -0.03 | 0.006 | 2.75e-08 | 31 | -0.038 | 0.0236 | 0.103 |
| 16 | rs369028384 | 13746413 | CTT/C | *U95743.1* | 0.036 | 0.006 | 1.88e-08 | 32 | -0.003 | 0.0237 | 0.896 |
| 16 | 16:19994176 | 19994176 | CT/C | *GPR139* | -0.04 | 0.006 | 8.91e-13 | 51 | -0.010 | 0.0237 | 0.681 |
| 16 | rs78621285 | 22903022 | A/T | *HS3ST2* | 0.056 | 0.01 | 4.88e-09 | 34 | 0.022 | 0.0373 | 0.548 |
| 16 | rs7191618 | 28565667 | C/G | *CCDC101* | 0.055 | 0.006 | 3.49e-23 | 98 | 0.024 | 0.0214 | 0.260 |
| 16 | rs200720048 | 30066332 | CCT/C | *ALDOA* | 0.05 | 0.006 | 3.61e-19 | 80 | 0.003 | 0.0215 | 0.883 |
| 16 | rs112607901 | 31003411 | G/GAC | *STX1B* | -0.03 | 0.006 | 1.64e-08 | 32 | 0.023 | 0.0204 | 0.253 |
| 16 | rs9937521 ^*^ | 53799296 | C/T | *FTO* | 0.037 | 0.006 | 1.90e-11 | 45 | -0.024 | 0.0214 | 0.261 |
| 16 | rs113441031 | 69763280 | C/T | *CTD-2033A16.3* | 0.042 | 0.007 | 4.52e-09 | 34 | -0.026 | 0.0282 | 0.349 |
| 16 | rs36182990 | 71992584 | G/A | *PKD1L3* | -0.04 | 0.006 | 8.48e-12 | 47 | 0.005 | 0.0238 | 0.840 |
| 16 | rs1104608 | 73912588 | G/C | *RPSAP56* | 0.032 | 0.006 | 1.04e-08 | 33 | -0.028 | 0.0212 | 0.187 |
| 17 | rs8073177 | 7440584 | T/C | *Y_RNA* | -0.04 | 0.007 | 2.58e-09 | 35 | 0.012 | 0.0246 | 0.621 |
| 17 | rs650558 | 40721042 | C/T | *MLX* | 0.039 | 0.006 | 5.52e-10 | 38 | -0.053 | 0.0244 | 0.031 |
| 17 | 17:43971206 | 43971206 | CTAATT/ C | *MAPT-AS1* | 0.057 | 0.007 | 3.03e-17 | 71 | 0.011 | 0.0263 | 0.683 |
| 18 | rs1788820 | 21101944 | A/G | *C18orf8:NPC1* | -0.05 | 0.006 | 7.90e-17 | 69 | 0.010 | 0.0222 | 0.667 |
| 18 | rs1834144 | 40744790 | C/A | *RIT2* | -0.03 | 0.006 | 5.17e-10 | 39 | -0.026 | 0.0218 | 0.235 |
| 18 | rs624244 | 53183396 | G/A | *TCF4* | 0.033 | 0.006 | 1.63e-08 | 32 | 0.004 | 0.0215 | 0.848 |
| 18 | rs6567160 | 57829135 | T/C | *RP11-795H16.3* | 0.043 | 0.006 | 2.90e-11 | 44 | 0.027 | 0.0250 | 0.274 |
| 19 | rs8103840 ^*,†^ | 49254955 | C/T | *FUT1* | 0.032 | 0.005 | 4.93e-09 | 34 | 0.042 | 0.0209 | 0.046 |

Chr, chromosome; Ref. allele, reference allele; Alt. allele, alternative allele; SE, standard error.

^*^ Variants related to more than one dietary trait.

^†^ Variants associated with colorectal cancer (p<0.05).

^‡^ Variants not available in analysis of association with colorectal cancer.

**Table S2.** Heritability estimations for the proportion of dietary intake variance due to genetic differences between individuals and number of significant loci for dietary factors

| **Dietary factor** | **Heritability (%)** | **Standard deviation (%)** | **Number of significant loci** |
| --- | --- | --- | --- |
| Red meat | 5.81 | 0.18 | 983 |
| Processed meat | 5.42 | 0.34 | 293 |
| Poultry | 3.50 | 0.15 | 1 |
| Total fish | 5.58 | 0.17 | 1,792 |
| Milk | 9.01 | 0.21 | 3,315 |
| Cheese | 10.48 | 0.22 | 2,047 |
| Total fruits | 7.83 | 0.19 | 2,990 |
| Total vegetables | 5.30 | 0.17 | 997 |
| Coffee | 6.26 | 0.18 | 2,789 |
| Tea | 8.34 | 0.20 | 3,292 |
| Alcohol | 9.71 | 0.21 | 2,967 |

**Table S3.** Summary of dietary instrumental variables excluding variants associated with multiple dietary phenotypes or colorectal cancer risk

| **Dietary factor** | **No. SNPs** | **F-statistics** | | |
| --- | --- | --- | --- | --- |
|  |  | **Total** | **Men** | **Women** |
| Red meat | 13 | 406 | 242 | 172 |
| Processed meat | 9 | 252 | 161 | 91 |
| Total fish | 24 | 738 | 319 | 422 |
| Milk | 37 | 1,341 | 679 | 663 |
| Cheese | 54 | 1,877 | 847 | 1,051 |
| Total fruits | 75 | 2,696 | 1,599 | 1,182 |
| Total vegetables | 48 | 1,347 | 384 | 1,043 |
| Coffee | 26 | 1,285 | 636 | 646 |
| Tea | 28 | 1,012 | 476 | 536 |
| Alcohol | 49 | 1,789 | 813 | 1,076 |

SNP, single nucleotide polymorphism.

**Table S4.** Mendelian randomisation estimates for associations of genetically dietary intake with colorectal cancer risk using reduced list of variants

| **Dietary factor** | **Outcome** | **Total** | | **Men** | | **Women** | |
| --- | --- | --- | --- | --- | --- | --- | --- |
|  |  | **HR (95% CI)** | **P_pleiotropy_** | **HR (95% CI)** | **P_pleiotropy_** | **HR (95% CI)** | **P_pleiotropy_** |
| Red meat | Colorectal cancer | 1.03 (0.55-1.92) | 0.68 | 1.13 (0.57-2.27) | 0.49 | 0.87 (0.28-2.68) | 0.33 |
|  | Colon cancer | 1.45 (0.68-3.10) | 0.97 | 1.69 (0.71-4.03) | 0.61 | 1.12 (0.30-4.18) | 0.54 |
|  | Rectal cancer | 0.51 (0.17-1.51) | 0.39 | 0.58 (0.18-1.81) | 0.70 | 0.44 (0.05-3.83) | 0.46 |
| Processed meat | Colorectal cancer | 0.51 (0.23-1.13) | 0.66 | 0.51 (0.22-1.16) | 0.28 | 0.51 (0.09-2.99) | >0.99 |
|  | Colon cancer | 0.90 (0.34-2.38) | 0.88 | 0.88 (0.31-2.48) | 0.59 | 0.92 (0.12-7.32) | >0.99 |
|  | Rectal cancer | **0.16 (0.04-0.65)** | 0.17 | **0.21 (0.05-0.79)** | 0.69 | 0.11 (0.00-3.15) | 0.69 |
| Total fish | Colorectal cancer | 0.89 (0.59-1.34) | 0.87 | 0.90 (0.51-1.57) | 0.92 | 0.86 (0.47-1.61) | 0.67 |
|  | Colon cancer | 0.94 (0.56-1.55) | 0.79 | 0.91 (0.45-1.85) | 0.93 | 0.96 (0.46-1.98) | 0.67 |
|  | Rectal cancer | 0.80 (0.39-1.64) | 0.73 | 0.88 (0.35-2.20) | 0.88 | 0.66 (0.20-2.15) | 0.25 |
| Milk | Colorectal cancer | 1.19 (0.74-1.91) | 0.69 | 1.46 (0.81-2.63) | 0.12 | 0.86 (0.39-1.85) | 0.96 |
|  | Colon cancer | 1.08 (0.61-1.93) | 0.42 | 1.32 (0.63-2.79) | 0.20 | 0.82 (0.33-2.03) | 0.97 |
|  | Rectal cancer | 1.45 (0.63-3.30) | 0.53 | 1.72 (0.66-4.50) | 0.91 | 0.95 (0.21-4.25) | 0.77 |
| Cheese | Colorectal cancer | 0.99 (0.78-1.25) | 0.86 | 0.98 (0.71-1.34) | 0.78 | 1.03 (0.72-1.47) | 0.97 |
|  | Colon cancer | 1.07 (0.80-1.42) | 0.68 | 1.16 (0.78-1.73) | 0.55 | 0.98 (0.65-1.48) | 0.90 |
|  | Rectal cancer | 0.85 (0.57-1.29) | 0.20 | 0.73 (0.43-1.22) | 0.86 | 1.19 (0.60-2.33) | 0.47 |
| Total fruits | Colorectal cancer | **0.78 (0.65-0.95)** | >0.99 | 0.85 (0.68-1.06) | 0.99 | **0.69 (0.50-0.96)** | 0.99 |
|  | Colon cancer | **0.78 (0.62-0.98)** | 0.91 | 0.85 (0.64-1.12) | 076 | 0.69 (0.47-1.01) | 0.99 |
|  | Rectal cancer | 0.80 (0.58-1.10) | >0.99 | 0.86 (0.60-1.23) | >0.99 | 0.69 (0.37-1.28) | >0.99 |
| Total vegetables | Colorectal cancer | 0.84 (0.70-1.01) | >0.99 | 0.81 (0.60-1.09) | >0.99 | 0.87 (0.68-1.11) | 0.94 |
|  | Colon cancer | 0.80 (0.63-1.01) | 0.99 | 0.76 (0.51-1.12) | >0.99 | 0.83 (0.61-1.11) | >0.99 |
|  | Rectal cancer | 0.93 (0.69-1.25) | >0.99 | 0.89 (0.56-1.42) | >0.99 | 0.97 (0.64-1.47) | >0.99 |
| Coffee | Colorectal cancer | 1.12 (0.87-1.43) | 0.73 | 1.08 (0.80-1.46) | 0.83 | 1.16 (0.77-1.74) | 0.70 |
|  | Colon cancer | 1.07 (0.79-1.45) | 0.52 | 0.97 (0.66-1.42) | 0.87 | 1.21 (0.75-1.96) | 0.49 |
|  | Rectal cancer | 1.22 (0.80-1.87) | 0.92 | 1.29 (0.79-2.12) | 0.78 | 1.03 (0.47-2.24) | 0.09 |
| Tea | Colorectal cancer | 0.96 (0.77-1.19) | 0.95 | 1.02 (0.77-1.34) | 0.74 | 0.88 (0.63-1.24) | 0.88 |
|  | Colon cancer | 0.85 (0.65-1.10) | 0.59 | 0.85 (0.60-1.21) | 0.41 | 0.84 (0.56-1.25) | 0.31 |
|  | Rectal cancer | 1.23 (0.85-1.79) | 0.70 | 1.37 (0.87-2.16) | 0.60 | 1.00 (0.52-1.91) | 0.44 |
| Alcohol | Colorectal cancer | 1.01 (0.86-1.20) | 0.95 | 1.14 (0.91-1.42) | 0.96 | 0.88 (0.69-1.14) | 0.62 |
|  | Colon cancer | 1.01 (0.82-1.24) | 0.50 | 1.12 (0.85-1.48) | 0.53 | 0.91 (0.68-1.23) | 0.78 |
|  | Rectal cancer | 1.02 (0.76-1.36) | 0.78 | 1.18 (0.82-1.69) | 0.90 | 0.81 (0.50-1.32) | 0.53 |

HR, hazard ratio; CI, confidence interval. P-values for pleiotropy effects are obtained from global test in Mendelian Randomisation Pleiotropy RESidual Sum and Outlier (MR-PRESSO). Bold font indicates significant difference.

**Table S5.** Cox proportional hazard estimates for associations between dietary intake and colorectal cancer for total study population and sex-specific subgroups in the UK Biobank

| **Dietary factor** | **Total (N=374,004)** | | **Men (N=174,576)** | | **Women (N=199,428)** | |
| --- | --- | --- | --- | --- | --- | --- |
|  | **Crude HR (95% CI)** | **Adjusted HR (95% CI)** | **Crude HR (95% CI)** | **Adjusted HR (95% CI)** | **Crude HR (95% CI)** | **Adjusted HR (95% CI)** |
| Red meat | **1.07 (1.05-1.09)** | **1.05 (1.03-1.07)** | **1.08 (1.05-1.10)** | **1.06 (1.04-1.09)** | 1.03 (1.00-1.06) | 1.03 (1.00-1.06) |
| Processed meat | **1.08 (1.06-1.10)** | **1.03 (1.01-1.05)** | **1.05 (1.03-1.08)** | **1.04 (1.02-1.07)** | 1.00 (0.96-1.04) | 1.00 (0.96-1.04) |
| Poultry | 1.00 (0.98-1.02) | 1.00 (0.98-1.02) | 1.02 (0.99-1.05) | 1.01 (0.98-1.05) | 0.98 (0.95-1.02) | 0.98 (0.95-1.02) |
| Total fish | 0.98 (0.96-1.00) | 0.98 (0.97-1.00) | 0.99 (0.96-1.01) | 0.99 (0.96-1.01) | 0.98 (0.95-1.00) | 0.98 (0.95-1.01) |
| Milk | **0.94 (0.92-0.97)** | **0.95 (0.92-0.97)** | **0.94 (0.90-0.97)** | **0.95 (0.92-0.99)** | **0.94 (0.90-0.98)** | **0.94 (0.90-0.98)** |
| Cheese | 1.01 (1.00-1.03) | 1.01 (0.99-1.02) | 0.99 (0.97-1.02) | 0.99 (0.97-1.02) | 1.02 (0.99-1.04) | 1.02 (1.00-1.05) |
| Total fruits | **0.97 (0.95-0.98)** | 0.99 (0.98-1.01) | **0.97 (0.95-0.99)** | 0.98 (0.96-1.00) | 1.00 (0.98-1.02) | 1.01 (0.98-1.03) |
| Total vegetables | 0.99 (0.98-1.00) | 0.99 (0.98-1.00) | 0.99 (0.98-1.00) | 0.99 (0.98-1.00) | 0.99 (0.98-1.01) | 0.99 (0.98-1.01) |
| Coffee | 1.01 (1.00-1.03) | 1.00 (0.99-1.01) | 1.02 (1.00-1.03) | 1.01 (0.99-1.03) | 0.99 (0.97-1.01) | 0.98 (0.96-1.01) |
| Tea | **0.98 (0.97-0.99)** | **0.98 (0.97-0.99)** | **0.98 (0.96-0.99)** | 0.98 (0.97-1.00) | 0.98 (0.97-1.00) | 0.98 (0.97-1.00) |
| Alcohol | **1.04 (1.03-1.05)** | **1.03 (1.01-1.04)** | **1.04 (1.02-1.05)** | **1.04 (1.02-1.06)** | 1.00 (0.98-1.02) | 1.00 (0.98-1.02) |

HR, hazard ratio; CI, confidence interval. Multivariable regression model is adjusted for sex (except for sex-specific analyses), family history of colorectal cancer, household income, smoking, alcohol consumption (except for alcohol consumption exposure), physical activity, and body mass index. Bold font indicates significant difference.

**Table S6.** Cox proportional hazard estimates for associations between dietary intake and colon cancer for total study population and sex-specific subgroups in the UK Biobank

| **Dietary factor** | **Total (N=374,004)** | | **Men (N=174,576)** | | **Women (N=199,428)** | |
| --- | --- | --- | --- | --- | --- | --- |
|  | **Crude HR (95% CI)** | **Adjusted HR (95% CI)** | **Crude HR (95% CI)** | **Adjusted HR (95% CI)** | **Crude HR (95% CI)** | **Adjusted HR (95% CI)** |
| Red meat | **1.07 (1.05-1.10)** | **1.05 (1.03-1.08)** | **1.08 (1.05-1.11)** | **1.07 (1.03-1.10)** | 1.04 (1.00-1.08) | 1.04 (1.00-1.08) |
| Processed meat | **1.06 (1.03-1.09)** | 1.02 (1.00-1.05) | **1.05 (1.02-1.08)** | **1.04 (1.01-1.07)** | 1.00 (0.95-1.04) | 0.99 (0.95-1.04) |
| Poultry | 1.00 (0.97-1.02) | 0.99 (0.97-1.02) | 1.00 (0.96-1.04) | 0.99 (0.95-1.03) | 1.00 (0.96-1.04) | 1.00 (0.96-1.04) |
| Total fish | **0.97 (0.94-0.99)** | 0.97 (0.95-1.00) | 0.98 (0.95-1.01) | 0.99 (0.96-1.02) | **0.95 (0.92-0.99)** | **0.96 (0.92-0.99)** |
| Milk | **0.94 (0.91-0.98)** | **0.94 (0.91-0.98)** | **0.94 (0.89-0.98)** | 0.95 (0.90-1.00) | **0.94 (0.89-0.99)** | **0.94 (0.89-0.99)** |
| Cheese | 1.00 (0.98-1.02) | 1.00 (0.98-1.02) | 0.98 (0.95-1.01) | 0.98 (0.95-1.01) | 1.01 (0.98-1.04) | 1.02 (0.99-1.05) |
| Total fruits | 0.98 (0.97-1.00) | 1.01 (0.99-1.02) | 0.98 (0.96-1.01) | 0.99 (0.97-1.02) | 1.01 (0.99-1.04) | 1.02 (0.99-1.05) |
| Total vegetables | 0.99 (0.98-1.00) | 0.99 (0.98-1.01) | 0.99 (0.98-1.01) | 0.99 (0.98-1.01) | 0.99 (0.97-1.01) | 0.99 (0.98-1.01) |
| Coffee | 1.01 (0.99-1.03) | 1.00 (0.98-1.02) | 1.02 (1.00-1.04) | 1.01 (0.99-1.04) | 0.98 (0.96-1.01) | 0.98 (0.95-1.01) |
| Tea | **0.98 (0.96-0.99)** | **0.98 (0.97-0.99)** | **0.97 (0.95-0.99)** | 0.98 (0.96-1.00) | 0.98 (0.96-1.00) | 0.98 (0.96-1.00) |
| Alcohol | **1.03 (1.01-1.04)** | 1.02 (1.00-1.14) | **1.03 (1.01-1.04)** | **1.03 (1.01-1.05)** | 1.00 (0.98-1.02) | 1.01 (0.99-1.03) |

HR, hazard ratio; CI, confidence interval. Multivariable regression model is adjusted for sex (except for sex-specific analyses), family history of colorectal cancer, household income, smoking, alcohol consumption (except for alcohol consumption exposure), physical activity, and body mass index. Bold font indicates significant difference.

**Table S7.** Cox proportional hazard estimates for associations between dietary intake and rectal cancer for total study population and sex-specific subgroups in the UK Biobank

| **Dietary factor** | **Total (N=374,004)** | | **Men (N=174,576)** | | **Women (N=199,428)** | |
| --- | --- | --- | --- | --- | --- | --- |
|  | **Crude HR (95% CI)** | **Adjusted HR (95% CI)** | **Crude HR (95% CI)** | **Adjusted HR (95% CI)** | **Crude HR (95% CI)** | **Adjusted HR (95% CI)** |
| Red meat | **1.08 (1.04-1.11)** | **1.05 (1.03-1.07)** | **1.07 (1.03-1.11)** | **1.06 (1.02-1.10)** | **1.08 (1.04-1.11)** | 1.01 (0.95-1.08) |
| Processed meat | **1.11 (1.08-1.15)** | **1.03 (1.01-1.05)** | **1.06 (1.02-1.10)** | 1.04 (1.00-1.09) | **1.11 (1.08-1.15)** | 1.02 (0.95-1.09) |
| Poultry | 1.01 (0.97-1.05) | 1.01 (0.98-1.02) | **1.06 (1.01-1.11)** | 1.05 (1.00-1.11) | 1.01 (0.97-1.05) | 0.94 (0.88-1.01) |
| Total fish | 1.00 (0.97-1.03) | 0.98 (0.97-1.00) | 1.00 (0.96-1.04) | 0.99 (0.95-1.03) | 1.00 (0.97-1.03) | 1.03 (0.98-1.09) |
| Milk | 0.95 (0.90-1.00) | **0.95 (0.92-0.97)** | 0.94 (0.88-1.00) | 0.96 (0.90-1.02) | 0.95 (0.90-1.00) | 0.94 (0.86-1.03) |
| Cheese | 1.03 (1.00-1.06) | 1.01 (0.99-1.02) | 1.01 (0.98-1.05) | 1.01 (0.97-1.05) | 1.03 (1.00-1.06) | 1.03 (0.98-1.08) |
| Total fruits | **0.92 (0.90-0.95)** | 0.99 (0.98-1.01) | **0.95 (0.91-0.98)** | **0.96 (0.92-0.99)** | **0.92 (0.90-0.95)** | 0.96 (0.92-1.01) |
| Total vegetables | 0.98 (0.97-1.00) | 0.99 (0.98-1.00) | 0.99 (0.97-1.01) | 0.99 (0.97-1.01) | 0.98 (0.97-1.00) | 0.99 (0.96-1.02) |
| Coffee | 1.02 (0.99-1.04) | 1.00 (0.99-1.01) | 1.01 (0.98-1.04) | 1.00 (0.97-1.04) | 1.02 (0.99-1.04) | 1.00 (0.96-1.05) |
| Tea | 0.99 (0.97-1.01) | **0.99 (0.97-0.99)** | 0.99 (0.96-1.01) | 0.99 (0.96-1.01) | 0.99 (0.97-1.01) | 0.99 (0.95-1.02) |
| Alcohol | **1.06 (1.04-1.08)** | **1.04 (1.02-1.06)** | **1.06 (1.03-1.08)** | **1.06 (1.04-1.09)** | 1.00 (0.96-1.03) | 0.99 (0.96-1.03) |

HR, hazard ratio; CI, confidence interval. Multivariable regression model is adjusted for sex (except for sex-specific analyses), family history of colorectal cancer, household income, smoking, alcohol consumption (except for alcohol consumption exposure), physical activity, and body mass index. Bold font indicates significant difference.

**Table S8.** Significant loci for dietary intake identified from previous genome-wide association studies

| **Study** | **Nearby gene** | **Variant** | **Possible biological mechanism** | **Food item/ dietary pattern** | **Population** | **Sample size** |
| --- | --- | --- | --- | --- | --- | --- |
| Mompeo et al., 2022 (12) | *NEGR1*, *ARPP21*, 5q12.1, 8p23.1, 12q13.12, *FTO*, 18q21.32 | rs66495454, rs56331918, rs544711163, rs73195303, rs1054442, rs56094641, rs35614134 | *ARPP21, RP11-62H7.2, MFHAS1 and RHEBL1* | Dietary Approaches to Stop Hypertension | UK Biobank | 173,701 |
| Suzuki et al. 2021 (13) | 14q11.2 locus | rs4982753 | *SLC22A1* plays a role in olfaction in mammals  *SLC22A1* and *BCL2L2* associate with obesity in Korea  *HOMEZ* associates with waist-to-hip ratio adjusted for BMI in European population | Japanese dietary pattern | Japanese (J-MICC Study) | 14,079 |
| Suziki et al. 2020 (14) | *BRAP* | rs3782886 | Anthropometric or immune response-related trait in Europeans and Africans but unknown mechanism in the association with fish intake | Fish consumption | Japanese (J-MICC Study) | 16,584 |
|  | *ALDH2* | rs671 | Acetaldehyde in fish meat gave some unpleasant taste or smell in participants with defective *ALDH2* genotype, and thus they ate less amount of fish |  |  |  |
|  | *ACAD10* | rs11066015 | Encode a member of the acyl-CoA dehydrogenase family of enzymes (ACADs) that participate in the beta-oxidation of fatty acids in mitochondria |  |  |  |
|  | *NAA25* | rs11066132 | Not described |  |  |  |
|  | *HECTD4* | rs144504271 | Encode E3 ubiquitin protein ligase |  |  |  |
| Niarchou et al. 2020 (15) | *FGF21, IZUMO1, MEF2, MAMSTR, RASIP1,* and others | 29 SNPs | Not described | Meat-related diet | UK Biobank | 335,576 |
|  |  | 63 SNPs |  | Fish and plant-related diet |  |  |
| Meddens et al. 2020 (16) | *APOE* | rs429358 | Affect fatty acid metabolism | Dietary carbohydrate | UK Biobank, Lifelines, RSI/II/III, ALSPAC, Fenland, FHS, HRS, GARNET, HIPFX, WHIMS+, and EPIC-InterAct and DietGen | 268,922 |
|  | *APOE* | rs429358 | Affect fatty acid metabolism | Dietary fat |  |  |
|  | *ADH1B* | rs1229984 | Inhibit alcohol metabolism |  |  |  |
|  | *FGF21* | rs838133 | Influence sweet and alcohol taste preference via the liver-brain-endocrine axis | Dietary protein |  |  |
|  | *KLB* | rs13146907 | Code an essential cofactor to *FGF21* |  |  |  |
|  | *GCKR* | rs780094 | Affect carbohydrate metabolism |  |  |  |
|  | *AOPE* | rs429358 | Affect fatty acid metabolism | Dietary sugar |  | 235,391 |
| Matoba et al. 2020 (17) | *GCKR, ADH1B, ADH1B1, ADH1A1,* and *ALDH2* | rs1260326, rs1229984, rs304, rs8187929, and rs671 | *ALDH2* and *ADH1B* involve in alcohol metabolism | Alcohol | Japan Biobank | 165,084 |
|  | *MCL1, ENSA, GCKR, AGR3, AHR, ALDH2, CYP1A2, CSK,* and *ADORA2A-AS1* | rs6681426, rs1260326, rs4410790, rs671, rs58806801, rs5760444 | Caffeine is an antagonist of the adenosine A1/A2a receptors *ADORA1* and *ADORA2A*  *CYP2* has role in coffee consumption and caffeine metabolism | Coffee |  |  |
|  | *ALDH2* | rs671 |  | Tea, milk, yoghurt, nattom tofu, fish |  |  |
| Furukawa et al. 2020 (18) | *ALDH2* | rs2074356, rs144504271, and rs12231737 | *ALDH2* involves in alcohol metabolism | Black tea consumption | Japanese | 12,258 |
| Cole et al., 2020 (19) |  | 814 SNPs |  | 85 food items + 85 dietary patterns | UK Biobank | 455,146 |
| Zhong et al. 2019 (20) | *GCKR, ABCG2, AHR, POR,* and *CYP1A1/2* | rs1260326, rs1481012, rs4410790, rs17685, rs2472297 | Not described | Total bitter beverages | UK Biobank | 125,776 |
|  | *ANXA9, AHR, POR, CYP1A1/2,* and *CSDC2* | rs12405726, rs4410790, rs7791070, rs35855035, rs1057868, rs2472297, rs9607819 |  | Bitter non-alcoholic beverages |  | 125,776 |
|  | *GCKR, KLB, ADH1B,* and *AGBL2* | rs1260326, rs11940694, rs1229984, rs7935528 |  | Bitter alcoholic beverages |  | 376,372 |
|  | *SEC16B, TMEM18, OR8U8, AKAP6, MC4R,* and *SPECC1L-ADORA2A* | rs574367, rs10865548, rs1260326, rs117692895, rs4410790, rs4719497, rs12699844, rs73073176, rs34060476, rs1057868, rs597045, rs1956218, rs2472297, rs66723169, rs2330783 | *ADORA2A* encodes the adenosine 2A receptor, a key target for caffeine  *OR8U8* encodes a member of the olfactory receptor family and thus functions in smell perception  *SEC16B, TMEM18, MC4R*, and *AKAP6* are BMI-related variants and BMI was associated with high coffee consumption  *FTO*, *TMEM18*, and *MC4R* are highly expressed in hypothalamus, a brain region regulating reward and motivation aspects of eating behavior | Coffee |  | 375,833 |
|  | *FTO* | rs55872725 | BMI-related gene | Sugar sweetened beverages |  | 125,776 |
| Jia et al. 2019 (21) | *AHR* | rs10252701 | Involve in caffeine metabolism and  encode a ligand-activated transcription factor that is an upstream inducer of CYP1A1 and CYP1A2 expression | Coffee consumption | Japanese | 12,239 |
|  | *CUX2* | rs79105258 | Near *AHR* gene |  |  |  |
| Kranzler et al. 2019 (22) | *ADH1B, ADH1C, GCKR, SLC39A8,* and *FTO* | 13 SNPs | *ADH1B* and *ADH1C* are alcohol metabolism genes  *GCKR, SLC39A8,* and *FTO* are pleiotropic genes | Alcohol consumption level | 5 American populations | 274,424 |
|  | *ADH1B, ADH1C, SLC39A8, GCKR, ADH4, SIX3,* and *DRD2* | 10 SNPs |  | Alcohol use disorder |  |  |
| Hwang et al. 2019 (23) | *FTO* | rs11642841 | BMI-increasing allele increases the intake of energy, dietary fat, and protein | Total sugar intake | 3 European populations | 176,867 |
| Gelernter et al. 2019 (24) | *ADH1B, XPO7, RNU6-53P,* and *CRHR1* | rs1229984, rs7821592, rs1577857, rs77804065, and rs2066702 | *ADH1B* involves in alcohol metabolism  *XPO7* produces protein product mediating nuclear export of proteins  *CRHR1* produces protein product involving in stress and immune response | Habitual alcohol intake | US European and African American Veterans | 143,965 |
| Nakagawa-Senda et al. 2018 (25) | Unknown | rs2074356 | Not applicable | Coffee consumption | Japanese (J-MICC Study) | 11,261 |
| Jiang et al. 2018 (26) | *ANKRD33* and *FIGNL2* | rs10876214, rs9669605, and rs10783478 | *ANKRD33* relates to photoreceptor signaling and *FIGNL2* relates to ATP binding, but linkage mechanism to energy intake is unclear | Daily energy intake | Nurses' Health Study I and II and Health Professionals Follow-up Study | 18,773 |
|  | *LOC107984317* and *KLF12* | rs142343672, rs146169233, and rs61957289 |  | Daily energy expenditure |  |  |
| Mozaffarian et al. 2017 (27) | Chromosome 6 | rs9502823 | Not described | Fish intake (servings/day) | 17 European cohorts (CHARGE) | 86,467 |
| Guenard et al. 2017 (28) |  | 78 SNPs |  | Prudent pattern | Fatty Acid Sensor study | 210 |
|  |  | 27 SNPs |  | Western pattern |  |  |
| Clarke et al. 2017 (29) | *ADH1B, ADH1C, ADH5, KLB, GCKR, CADM2,* and *FAM69C* | 14 SNPs | *ADH1B, ADH1C,* and *ADH5* are alcohol metabolizing genes  *KLB* associates with alcohol consumption  *GCKR* encodes the glucokinase regulatory protein that is responsible for phosphorylation of glucose in the liver  *CADM2* is a brain expressed gene encoding cell adhesion molecule 2 and associates with processing speed, educational attainment, and lifetime cannabis. | Alcohol consumption | UK Biobank | 112,117 |
| Pirastu et al. 2016 (30) | *PDSS2* | rs6568479 | Code for an enzyme responsible for the synthesis of the prenyl side chain of coenzyme Q10 | Coffee consumption | 2 Italian populations | 2,938 |
| Cornelis et al. 2016 (31) | *AHR, CYP1A2, CYP2A6, GCKR, ABCG2,* and *POR* | Variants at 7p21, 15q24, 19q13.2, 2p24, 4q22, and 7q11.23 | *ABCG2* is expressed in apical membranes of the liver, kidney, intestine, and brain, and plays an important role in preventing absorption and excessive accumulation of numerous xenobiotic and endogenous substrates in certain tissues | Coffee consumption | UK Biobank | 502,650 |
| Rudkowska et al. 2015 (32) |  | 54 SNPs |  | Total fat intake | US (Laval University) | 541 |
| Wakai et al. 2013 (33) | *ADIPOQ* | rs822396 | Genetic polymorphisms of *ADIPOQ* around the rs822396 SNP might control blood adiponectin levels, and could be associated with a propensity to favor foods of high-energy density such as confectionery | Confectionery intake | Japan (HERPACC-II and J-MICC) | 5,430 |
| Baik et al. 2011 (34) | *12q24 locus* | 12 SNPs |  | Alcohol consumption | Korean men | 2,834 |

SNP, single-nucleotide polymorphism; J-MICC, Japan Multi-Institutional Collaborative Cohort; CHARGE, Childhood Autism Risks from Genetics and the Environment; HERPACC-II, Hospital-based Epidemiologic Research Program at Aichi Cancer Center II.

**Table S9.** Multivariable Mendelian randomization estimates for associations of genetically dietary intake with colorectal cancer risk using full list of variants

| **Dietary factor** | **Total** | **Men** | **Women** |
| --- | --- | --- | --- |
| **Red meat** |  |  |  |
| Colorectal cancer | **1.30 (1.19-1.43)** | **1.20 (1.05-1.37)** | 0.96 (0.82-1.13) |
| Colon cancer | **1.21 (1.08-1.35)** | **1.19 (1.01-1.40)** | 0.93 (0.77-1.13) |
| Rectal cancer | **1.51 (1.30-1.76)** | 1.21 (0.98-1.50) | 1.03 (0.76-1.40) |
| **Processed meat** |  |  |  |
| Colorectal cancer | **1.29 (1.18-1.41)** | **1.36 (1.21-1.53)** | 1.18 (0.99-1.41) |
| Colon cancer | **1.31 (1.18-1.46)** | **1.38 (1.19-1.60)** | **1.25 (1.02-1.54)** |
| Rectal cancer | **1.25 (1.07-1.46)** | **1.31 (1.08-1.60)** | 1.00 (0.71-1.42) |
| **Total fish** |  |  |  |
| Colorectal cancer | 1.01 (0.86-1.19) | 1.02 (0.83-1.26) | 1.19 (0.93-1.53) |
| Colon cancer | 1.19 (0.98-1.45) | 1.11 (0.85-1.43) | **1.45 (1.08-1.94)** |
| Rectal cancer | **0.71 (0.54-0.95)** | 0.90 (0.64-1.26) | 0.71 (0.44-1.13) |
| **Milk** |  |  |  |
| Colorectal cancer | 1.01 (0.96-1.06) | 1.02 (0.96-1.08) | 0.94 (0.87-1.02) |
| Colon cancer | 0.98 (0.92-1.04) | 1.01 (0.93-1.09) | 0.91 (0.83-1.00) |
| Rectal cancer | 1.06 (0.98-1.15) | 1.04 (0.94-1.14) | 1.00 (0.86-1.16) |
| **Cheese** |  |  |  |
| Colorectal cancer | **1.36 (1.21-1.53)** | **1.38 (1.17-1.63)** | 0.99 (0.83-1.19) |
| Colon cancer | **1.24 (1.08-1.43)** | **1.28 (1.04-1.58)** | 1.00 (0.80-1.23) |
| Rectal cancer | **1.63 (1.34-1.99)** | **1.56 (1.19-2.05)** | 0.98 (0.69-1.39) |
| **Total fruit** |  |  |  |
| Colorectal cancer | 0.95 (0.88-1.03) | 0.97 (0.88-1.07) | **0.86 (0.76-0.98)** |
| Colon cancer | **0.88 (0.80-0.97)** | 0.93 (0.82-1.06) | **0.78 (0.67-0.91)** |
| Rectal cancer | 1.13 (0.98-1.30) | 1.04 (0.88-1.23) | 1.12 (0.88-1.44) |
| **Total vegetables** |  |  |  |
| Colorectal cancer | **0.94 (0.90-0.98)** | 0.94 (0.88-1.00) | 0.98 (0.91-1.04) |
| Colon cancer | **0.94 (0.89-0.99)** | 0.95 (0.88-1.03) | 0.95 (0.88-1.03) |
| Rectal cancer | 0.93 (0.86-1.01) | 0.93 (0.84-1.03) | 1.04 (0.91-1.18) |
| **Coffee** |  |  |  |
| Colorectal cancer | 0.99 (0.97-1.01) | 0.99 (0.97-1.02) | 0.98 (0.94-1.01) |
| Colon cancer | 1.00 (0.97-1.02) | 1.00 (0.97-1.04) | 0.98 (0.94-1.02) |
| Rectal cancer | 0.98 (0.94-1.02) | 0.98 (0.93-1.02) | 0.96 (0.90-1.03) |
| **Tea** |  |  |  |
| Colorectal cancer | **0.97 (0.95-0.99)** | **0.96 (0.94-0.98)** | 0.99 (0.97-1.02) |
| Colon cancer | 0.98 (0.95-1.00) | **0.96 (0.93-0.99)** | 1.00 (0.97-1.04) |
| Rectal cancer | **0.96 (0.93-0.99)** | 0.96 (0.92-1.00) | 0.97 (0.92-1.03) |
| **Alcohol** |  |  |  |
| Colorectal cancer | 1.03 (0.95-1.12) | 0.99 (0.88-1.11) | 1.07 (0.93-1.22) |
| Colon cancer | 1.00 (0.91-1.11) | 0.94 (0.82-1.09) | 1.07 (0.92-1.25) |
| Rectal cancer | 1.08 (0.94-1.25) | 1.07 (0.89-1.29) | 1.06 (0.82-1.36) |

Data are presented as hazard ratios (95% confidence intervals). Bold font indicates significant difference.

GO biological process


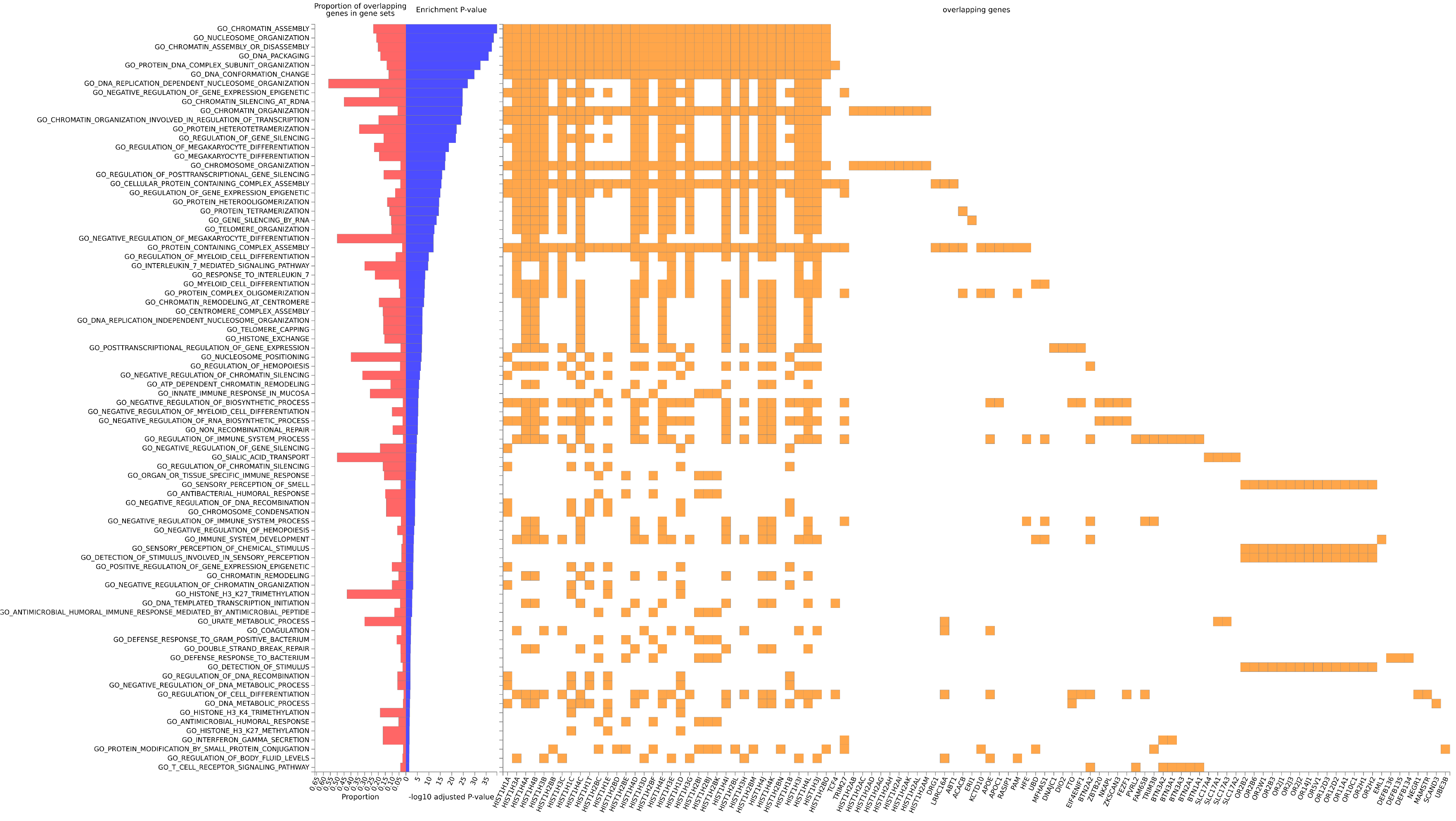


Figure S1 (cont.)

GO molecular functions


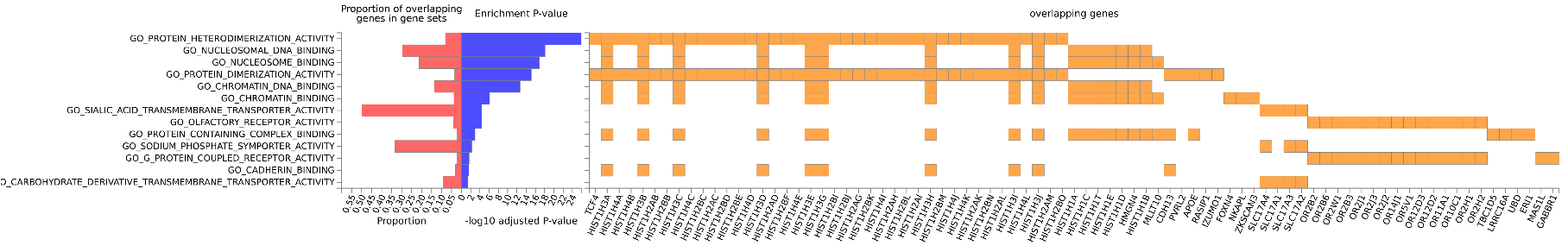


Wikipathways


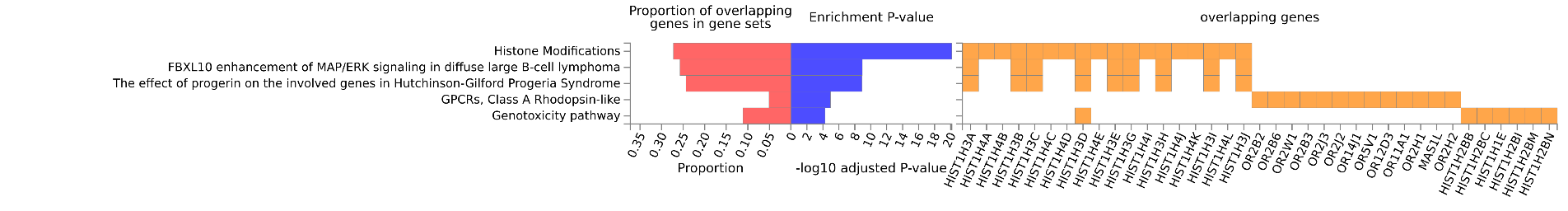


**Figure S1.** Gene set enrichment test to identify putative biological mechanisms of prioritized genes for fish intake

GO biological process


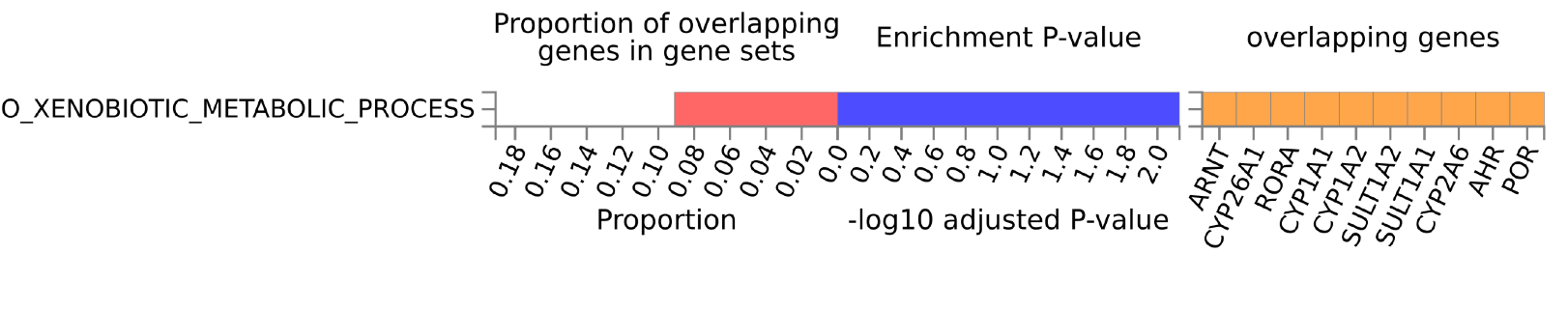


GO molecular functions


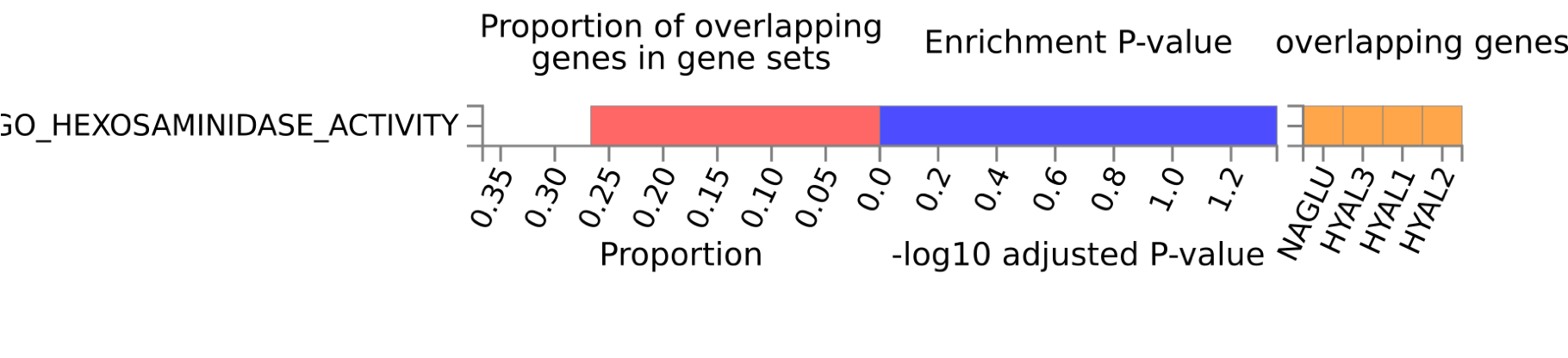


**Figure S2.** Gene set enrichment test to identify putative biological mechanisms of prioritized genes for milk intake

GO molecular functions


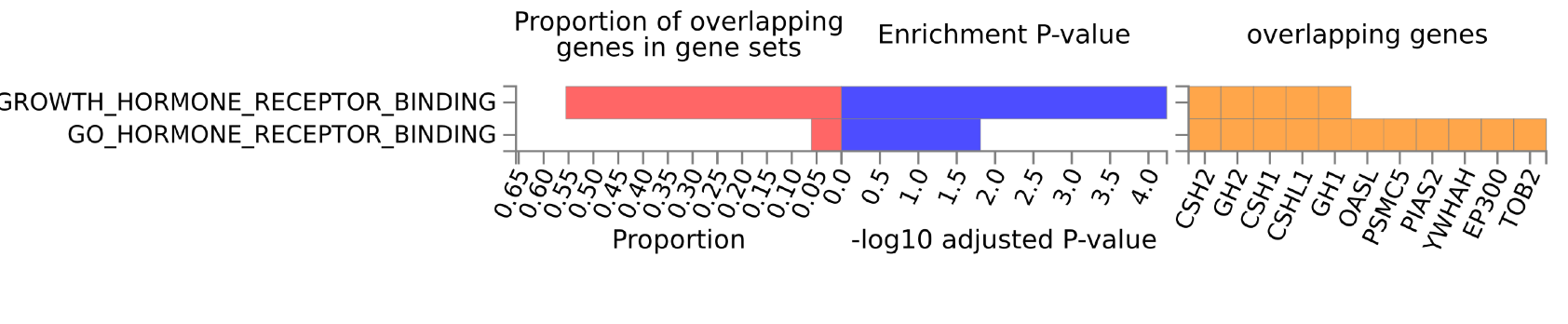


**Figure S3.** Gene set enrichment test to identify putative biological mechanisms of prioritized genes for cheese intake

GO molecular functions


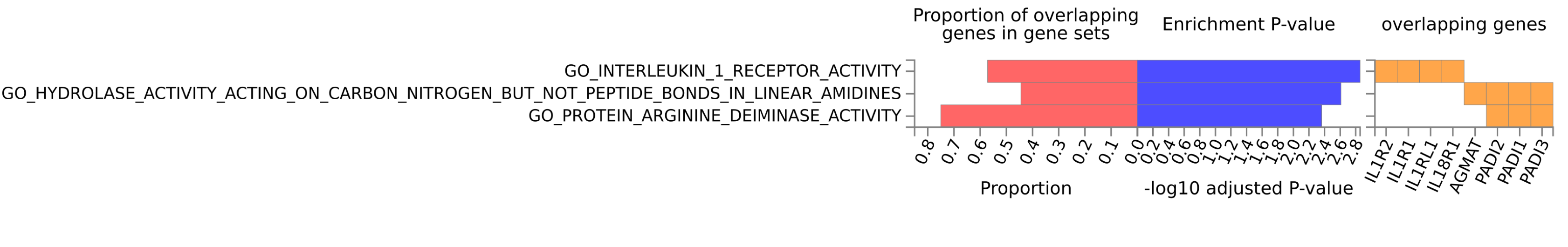


**Figure S4.** Gene set enrichment test to identify putative biological mechanisms of prioritized genes for fruit intake

GO biological processes


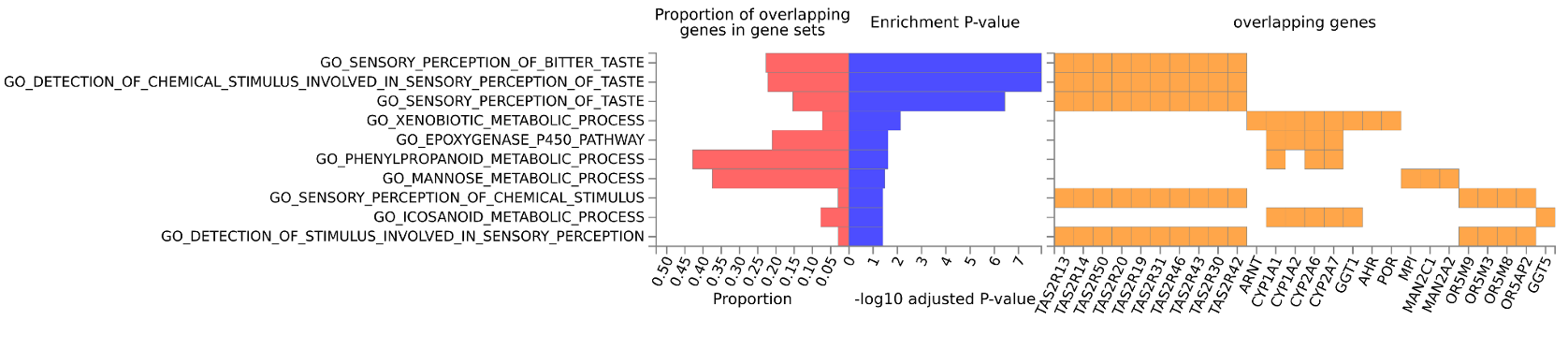


GO molecular functions


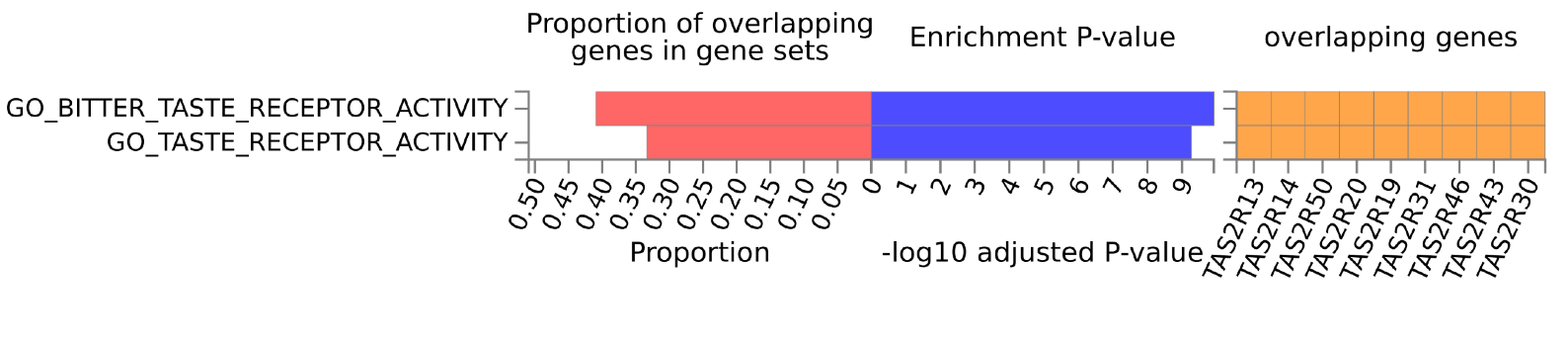


Wikipathways


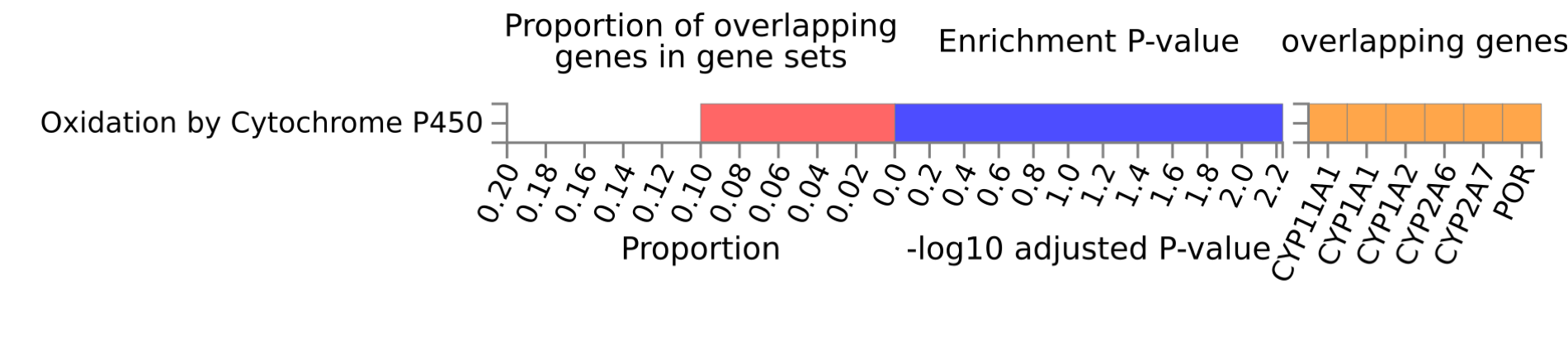


**Figure S5.** Gene set enrichment test to identify putative biological mechanisms of prioritized genes for coffee consumption

GO biological processes


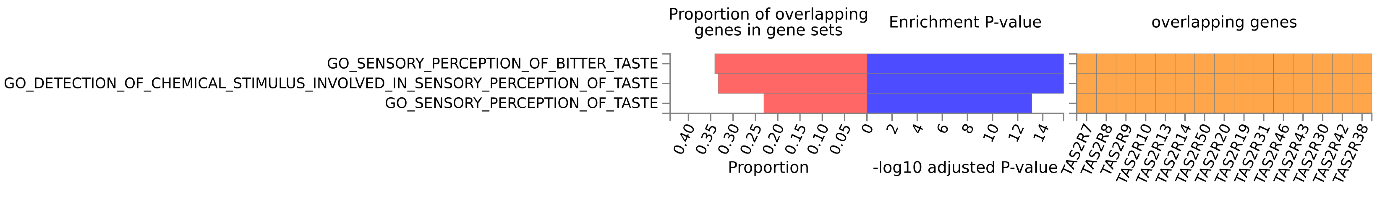


GO molecular functions


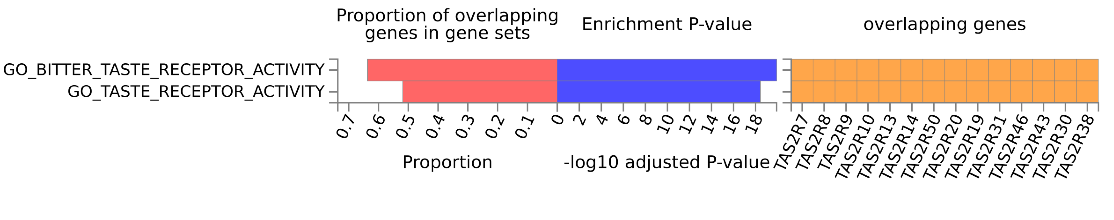


Wikipathways


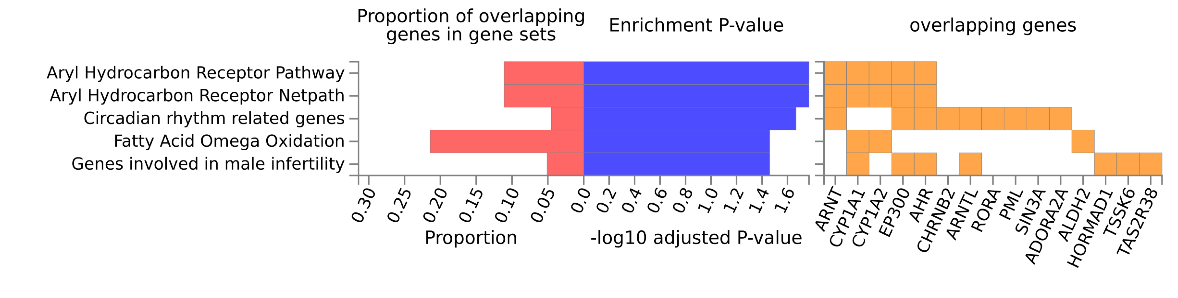


**Figure S6.** Gene set enrichment test to identify putative biological mechanisms of prioritized genes for tea consumption

GO biological processes


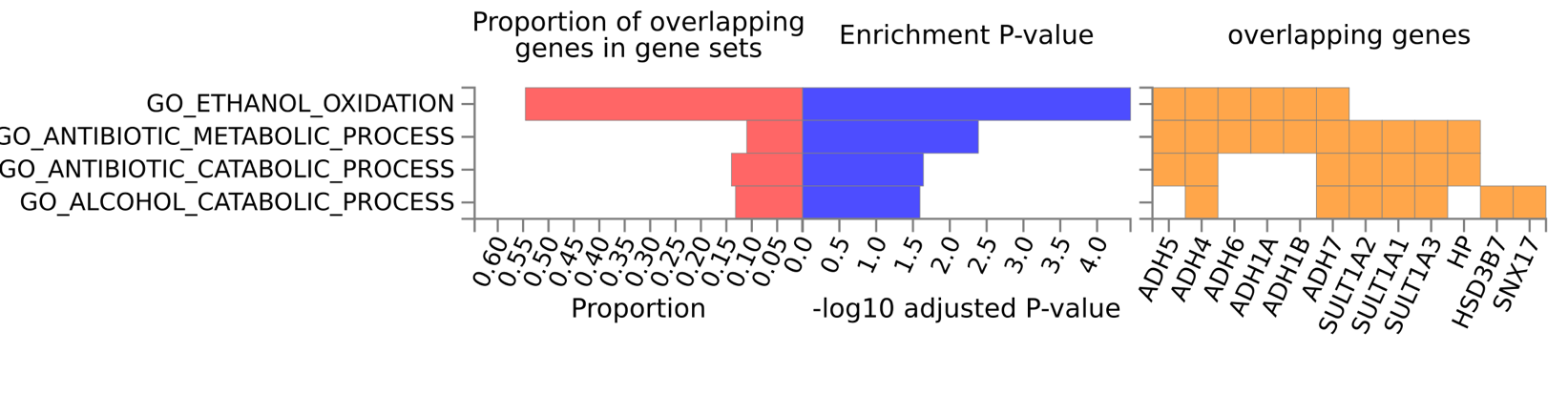


GO molecular functions


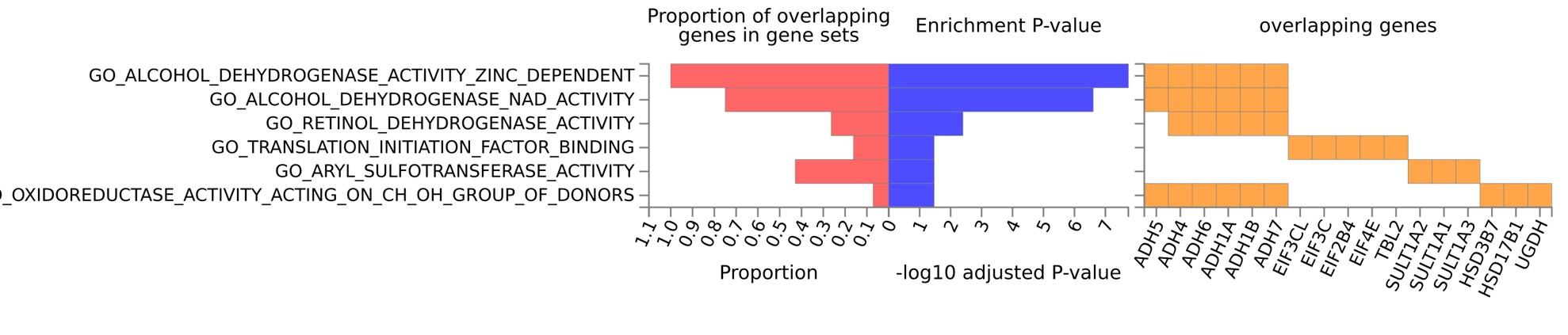


Wikipathways


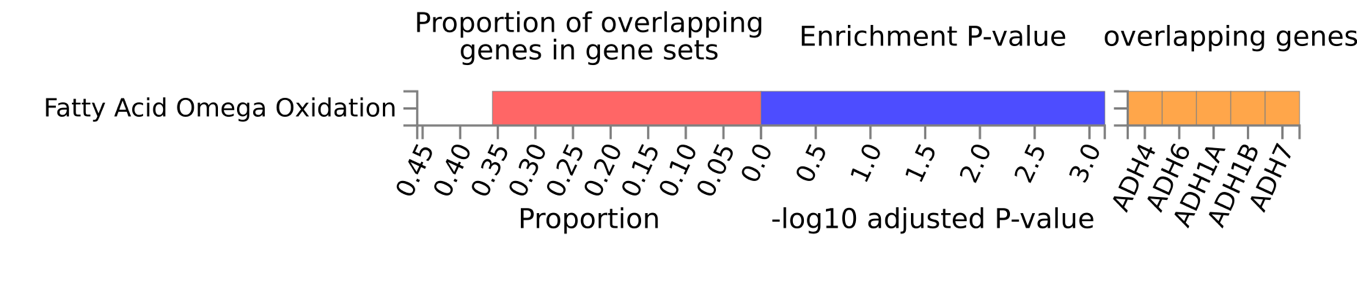


**Figure S7.** Gene set enrichment test to identify putative biological mechanisms of prioritized genes for alcohol consumption

**Figure S8.** Heatmap of pairwise genetic and phenotype correlation coefficients between food items

**Figure S9.** Sparse network for (A) Pearson correlations among allele scores and (B) genetic correlations of dietary factors

**Figure S10.** Summary of findings for genome-wide association study for food intake

**Figure S11.** Summary of findings for genetically predicted dietary intake and colorectal cancer risk

**References**

1. Yang J, Lee SH, Goddard ME, Visscher PM. GCTA: a tool for genome-wide complex trait analysis. Am J Hum Genet. 2011;88(1):76-82.

2. Jiang L, Zheng Z, Qi T, Kemper KE, Wray NR, Visscher PM, et al. A resource-efficient tool for mixed model association analysis of large-scale data. Nat Genet. 2019;51(12):1749-55.

3. Bycroft C, Freeman C, Petkova D, Band G, Elliott LT, Sharp K, et al. The UK Biobank resource with deep phenotyping and genomic data. Nature. 2018;562(7726):203-9.

4. Yang J, Benyamin B, McEvoy BP, Gordon S, Henders AK, Nyholt DR, et al. Common SNPs explain a large proportion of the heritability for human height. Nat Genet. 2010;42(7):565-9.

5. Turner S. Package ‘qqman’: Q-Q and Manhattan plots for GWAS data (version 0.1.8) 2022 [Available from: <https://github.com/stephenturner/qqman>].

6. Purcell S, Chang C. plink2 [Available from: <www.cog-genomics.org/plink/2.0/>].

7. Chang CC, Chow CC, Tellier LC, Vattikuti S, Purcell SM, Lee JJ. Second-generation PLINK: rising to the challenge of larger and richer datasets. Gigascience. 2015;4:7.

8. Watanabe K, Taskesen E, van Bochoven A, Posthuma D. Functional mapping and annotation of genetic associations with FUMA. Nat Commun. 2017;8(1):1826.

9. Speed D, Kaphle A, Balding DJ. SNP-based heritability and selection analyses: Improved models and new results. Bioessays. 2022;44(5):e2100170.

10. Wickham H, Chang W, Henry L, Pedersen TL, Takahashi K, Wilke C, et al. Package ‘ggplot2’: Create elegant data visualisations using the grammar of graphics 2022 [Available from: <https://ggplot2.tidyverse.org>].

11. Dey R, Zhou W, Kiiskinen T, Havulinna A, Elliott A, Karjalainen J, et al. Efficient and accurate frailty model approach for genome-wide survival association analysis in large-scale biobanks. Nat Commun. 2022;13(1):5437.

12. Mompeo O, Freidin MB, Gibson R, Hysi PG, Christofidou P, Segal E, et al. Genome-wide association analysis of over 170,000 individuals from the UK Biobank identifies seven loci associated with dietary approaches to stop hypertension (DASH) diet. Nutrients. 2022;14(20).

13. Suzuki H, Nakamura Y, Matsuo K, Imaeda N, Goto C, Narita A, et al. A genome-wide association study in Japanese identified one variant associated with a preference for a Japanese dietary pattern. Eur J Clin Nutr. 2021;75(6):937-45.

14. Suzuki T, Nakamura Y, Matsuo K, Oze I, Doi Y, Narita A, et al. A genome-wide association study on fish consumption in a Japanese population-the Japan Multi-Institutional Collaborative Cohort study. Eur J Clin Nutr. 2021;75(3):480-8.

15. Niarchou M, Byrne EM, Trzaskowski M, Sidorenko J, Kemper KE, McGrath JJ, et al. Genome-wide association study of dietary intake in the UK biobank study and its associations with schizophrenia and other traits. Transl Psychiatry. 2020;10(1):51.

16. Meddens SFW, de Vlaming R, Bowers P, Burik CAP, Linner RK, Lee C, et al. Genomic analysis of diet composition finds novel loci and associations with health and lifestyle. Mol Psychiatry. 2020.

17. Matoba N, Akiyama M, Ishigaki K, Kanai M, Takahashi A, Momozawa Y, et al. GWAS of 165,084 Japanese individuals identified nine loci associated with dietary habits. Nat Hum Behav. 2020;4(3):308-16.

18. Furukawa K, Igarashi M, Jia H, Nogawa S, Kawafune K, Hachiya T, et al. A genome-wide association study identifies the association between the 12q24 locus and black tea consumption in Japanese populations. Nutrients. 2020;12(10).

19. Cole JB, Florez JC, Hirschhorn JN. Comprehensive genomic analysis of dietary habits in UK Biobank identifies hundreds of genetic associations. Nat Commun. 2020;11(1):1467.

20. Zhong VW, Kuang A, Danning RD, Kraft P, van Dam RM, Chasman DI, et al. A genome-wide association study of bitter and sweet beverage consumption. Hum Mol Genet. 2019;28(14):2449-57.

21. Jia H, Nogawa S, Kawafune K, Hachiya T, Takahashi S, Igarashi M, et al. GWAS of habitual coffee consumption reveals a sex difference in the genetic effect of the 12q24 locus in the Japanese population. BMC Genet. 2019;20(1):61.

22. Kranzler HR, Zhou H, Kember RL, Vickers Smith R, Justice AC, Damrauer S, et al. Genome-wide association study of alcohol consumption and use disorder in 274,424 individuals from multiple populations. Nat Commun. 2019;10(1):1499.

23. Hwang LD, Lin C, Gharahkhani P, Cuellar-Partida G, Ong JS, An J, et al. New insight into human sweet taste: a genome-wide association study of the perception and intake of sweet substances. Am J Clin Nutr. 2019;109(6):1724-37.

24. Gelernter J, Sun N, Polimanti R, Pietrzak RH, Levey DF, Lu Q, et al. Genome-wide association study of maximum habitual alcohol intake in >140,000 U.S. European and African American Veterans yields novel risk loci. Biol Psychiatry. 2019;86(5):365-76.

25. Nakagawa-Senda H, Hachiya T, Shimizu A, Hosono S, Oze I, Watanabe M, et al. A genome-wide association study in the Japanese population identifies the 12q24 locus for habitual coffee consumption: The J-MICC Study. Sci Rep. 2018;8(1):1493.

26. Jiang L, Penney KL, Giovannucci E, Kraft P, Wilson KM. A genome-wide association study of energy intake and expenditure. PLoS One. 2018;13(8):e0201555.

27. Mozaffarian D, Dashti HS, Wojczynski MK, Chu AY, Nettleton JA, Mannisto S, et al. Genome-wide association meta-analysis of fish and EPA+DHA consumption in 17 US and European cohorts. PLoS One. 2017;12(12):e0186456.

28. Guenard F, Bouchard-Mercier A, Rudkowska I, Lemieux S, Couture P, Vohl MC. Genome-Wide Association Study of Dietary Pattern Scores. Nutrients. 2017;9(7).

29. Clarke TK, Adams MJ, Davies G, Howard DM, Hall LS, Padmanabhan S, et al. Genome-wide association study of alcohol consumption and genetic overlap with other health-related traits in UK Biobank (N=112 117). Mol Psychiatry. 2017;22(10):1376-84.

30. Pirastu N, Kooyman M, Robino A, van der Spek A, Navarini L, Amin N, et al. Non-additive genome-wide association scan reveals a new gene associated with habitual coffee consumption. Sci Rep. 2016;6:31590.

31. Cornelis MC, Kacprowski T, Menni C, Gustafsson S, Pivin E, Adamski J, et al. Genome-wide association study of caffeine metabolites provides new insights to caffeine metabolism and dietary caffeine-consumption behavior. Hum Mol Genet. 2016;25(24):5472-82.

32. Rudkowska I, Perusse L, Bellis C, Blangero J, Despres JP, Bouchard C, et al. Interaction between Common Genetic Variants and Total Fat Intake on Low-Density Lipoprotein Peak Particle Diameter: A Genome-Wide Association Study. J Nutrigenet Nutrigenomics. 2015;8(1):44-53.

33. Melkonian SC, Daniel CR, Hildebrandt MA, Tannir NM, Ye Y, Chow WH, et al. Joint association of genome-wide association study-identified susceptibility loci and dietary patterns in risk of renal cell carcinoma among non-Hispanic whites. Am J Epidemiol. 2014;180(5):499-507.

34. Baik I, Cho NH, Kim SH, Han BG, Shin C. Genome-wide association studies identify genetic loci related to alcohol consumption in Korean men. Am J Clin Nutr. 2011;93(4):809-16.
