# Supplementary material for: Genetically predicted dietary intake and risks of colorectal cancer: a Mendelian randomisation study
Source: BMC Cancer. 2024 Sep 17;24:1153. doi: 10.1186/s12885-024-12923-1 (PMC11409808; doi:10.1186/s12885-024-12923-1)
Supplement: Supplementary file 3 — Supplementary Material 3. [file 12885_2024_12923_MOESM3_ESM.docx]

**Additional file 3: Appendix**

By obtaining summary statistics of more than 11 million SNPs from the most recent comprehensive GWAS for dietary intake (1), we identified 231 variants associated with the intake of red meat (n=9), processed meat (n=7), poultry (n=1), total fish (n=12), milk (n=50), cheese (n=38), total fruits (n=41), total vegetables (n=42), coffee (n=11), tea (n=13), and alcohol (n=16) which were either not available in the previous study or did not reach the significance level (p<5x10^-8^). Of these, almost half of SNPs (n=107) were not available in Cole et al.’s study due to their smaller scale of imputed genetic data, and 70 SNPs reached the threshold for suggestive significance (5e-8≤p<1e-5). Among 54 loci that did not reach the suggestive significance level (p≥1e-5), 41 loci were for milk intake. However, the amount of daily milk consumption was not evaluated in the previous study, and we identified the remaining 12 novel loci for red meat (rs12144834, *AL592205.1*; rs150877559, *DLEU1*; rs12938702, *ST6GALNAC1*; and rs7251466, *ZNF574*), poultry (rs34473833, *CDH11*), total fish (rs4600686, *RNU6-812P*), cheese (rs12472445, *RBMS1*; rs4886168, *RNU7-88P*; and rs276950, *LINC01082*), total fruits (rs34156224, *AQP4-AS1:CHST9*), and coffee (rs2682909, *RP11-307C19.2*). Since the previous study performed genome-wide association analysis for types of milk only, all genomic risk loci for our estimated milk intake were either unavailable or did not reach a significance level of 5x10^-8^, and thus were determined to be novel loci in this study.

By including more than double SNPs compared to the previous study and adjusting for familial relatedness, the point estimates of heritability from summary statistics were slightly lower than those calculated in Cole et al.’s study (processed meat, 5.42% vs. 6.6%; poultry, 3.50% vs. 4.9%; cheese, 10.48% vs. 10.8%; coffee, 6.26% vs. 7.9%; tea, 8.34% vs. 9.1%; and alcohol, 12.1% vs. 9.71%)) (1). Nevertheless, no statistical tests were available to inform the significant difference. The heritability of food groups (red meat, total fish, total fruits, and total vegetables) appeared to be in the range of the heritability of corresponding food items. However, we were unable to compare the heritability of milk because milk intake was not assessed as a quantitative trait in the previous study.

**Summary statistics of genomic risk loci identified from genome-wide association analysis of red meat consumption**

| **Chr** | **Variant** | **Position** | **Reference allele** | **Alternative allele** | **RAF** | **Beta** | **SE** | **P_FastGWA_^*^** | **P_beef_^†^** | **P_pork_^†^** | **P_lamb/mutton_^†^** |
| --- | --- | --- | --- | --- | --- | --- | --- | --- | --- | --- | --- |
| 1 | rs2055145 | 45926495 | C | G | 0.762 | 0.0217 | 0.0036 | 2.06e-09 | 2.30e-04 | 4.60e-07 | 2.00e-06 |
| 1 | rs10789340 | 72940273 | A | G | 0.376 | 0.0196 | 0.0032 | 8.15e-10 | 3.40e-14 | 4.80e-06 | 8.00e-09 |
| 1 | rs11210240 ^‡^ | 73896101 | C | A | 0.812 | 0.0220 | 0.0039 | 2.46e-08 | 2.80e-07 | 2.60e-02 | 6.00e-02 |
| 1 | rs12144834 ^‡^ | 97352352 | T | G | 0.981 | -0.0678 | 0.0118 | 1.08e-08 | 9.30e-05 | 1.50e-04 | 4.60e-03 |
| 2 | rs1451077 | 147901207 | G | A | 0.415 | 0.0187 | 0.0031 | 2.63e-09 | 2.00e-07 | 7.20e-09 | 3.30e-07 |
| 3 | rs61791721 ^‡^ | 135804550 | T | A | 0.770 | 0.0212 | 0.0037 | 7.51e-09 | 1.90e-07 | 9.70e-07 | 2.70e-07 |
| 5 | rs576361041 ^‡^ | 43358175 | G | A | 1.000 | -0.7259 | 0.1329 | 4.73e-08 | NA | NA | NA |
| 5 | 5:124101388 ^‡^ | 124101388 | ATAT | A | 0.923 | 0.0335 | 0.0059 | 1.48e-08 | NA | NA | NA |
| 6 | rs4486004 | 26167710 | G | T | 0.780 | 0.0253 | 0.0037 | 1.09e-11 | 4.10e-09 | 3.60e-10 | 2.30e-06 |
| 7 | rs554034302 ^‡^ | 138227194 | G | T | 0.999 | -0.2674 | 0.0487 | 4.12e-08 | NA | NA | NA |
| 9 | rs141229573 | 15576114 | T | TATC | 0.525 | 0.0283 | 0.0031 | 7.74e-20 | 1.30e-13 | 1.80e-10 | 4.10e-18 |
| 13 | rs150877559 ^‡^ | 50701733 | G | A | 0.974 | -0.0537 | 0.0098 | 4.83e-08 | 2.00e-04 | 6.00e-03 | 8.30e-04 |
| 16 | rs12931387 | 5676432 | A | C | 0.808 | -0.0249 | 0.0039 | 2.26e-10 | 3.30e-04 | 3.10e-04 | 3.50e-09 |
| 17 | rs12938702 ^‡^ | 74607174 | C | T | 0.228 | 0.0215 | 0.0037 | 5.70e-09 | 1.40e-04 | 9.10e-04 | 6.00e-02 |
| 19 | rs7251466 ^‡^ | 42576952 | G | C | 0.890 | -0.0295 | 0.0049 | 2.29e-09 | 3.10e-07 | 5.00e-04 | 1.90e-05 |

Chr, chromosome; RAF, reference allele frequency; SE, standard error; NA, not available.

^*^ P-values were calculated in the present study using FastGWA tool for linear mixed model framework.

^†^ P-values were obtained from summary statistics of more than 11 million single nucleotide polymorphisms for overall beef, pork, and lamb/mutton intake in previous study. Summary statistic data can be downloaded at <https://personal.broadinstitute.org/ryank/Cole_UKB_Diet_GWAS.tar>. Source: Cole JB, Florez JC, Hirschhorn JN. Comprehensive genomic analysis of dietary habits in UK Biobank identifies hundreds of genetic associations. Nat Commun. 2020;11(1):1467.

^‡^ Loci were either not available in Cole et al.’s study or not reach significant level of 5e-08.

**Summary statistics of genomic risk loci identified from genome-wide association analysis of processed meat consumption**

| **Chr** | **Variant** | **Position** | **Reference allele** | **Alternative allele** | **RAF** | **Beta** | **SE** | **P_FastGWA_^*^** | **P_processed meat_^†^** |
| --- | --- | --- | --- | --- | --- | --- | --- | --- | --- |
| 2 | rs17676243 ^‡^ | 173322809 | G | A | 0.797 | 0.0204 | 0.0037 | 3.59e-08 | 8.10e-08 |
| 3 | rs9809856 | 18227421 | A | G | 0.521 | -0.0178 | 0.0030 | 2.11e-09 | 4.80e-09 |
| 3 | rs6765179 ^‡^ | 25276416 | G | A | 0.690 | 0.0184 | 0.0032 | 9.07e-09 | 3.60e-07 |
| 3 | rs13091492 | 81891476 | A | G | 0.625 | -0.0185 | 0.0031 | 1.40e-09 | 2.90e-10 |
| 4 | rs7695118 ^‡^ | 149012794 | A | G | 0.404 | 0.0184 | 0.0030 | 1.51e-09 | 4.40e-08 |
| 8 | rs2980508 | 8171732 | G | A | 0.511 | 0.0194 | 0.0030 | 5.76e-11 | 5.20e-09 |
| 8 | rs113442811 | 10772255 | C | CACAGA  AGA | 0.508 | -0.0230 | 0.0030 | 9.45e-15 | 3.40e-13 |
| 9 | rs147845411 ^‡^ | 24756659 | G | T | 0.983 | -0.0661 | 0.0120 | 3.68e-08 | 4.70e-07 |
| 11 | rs11030328 ^‡^ | 28447142 | G | A | 0.516 | -0.0167 | 0.0030 | 1.84e-08 | 8.00e-08 |
| 11 | rs61880662 ^‡^ | 30901989 | A | G | 0.851 | 0.0246 | 0.0043 | 9.52e-09 | 1.60e-08 |
| 11 | rs11032362 ^‡^ | 33759092 | G | A | 0.910 | 0.0323 | 0.0052 | 3.61e-10 | 3.20e-08 |
| 19 | rs8103840 | 49254955 | C | T | 0.531 | -0.0196 | 0.0030 | 5.90e-11 | 2.90e-09 |

Chr, chromosome; RAF, reference allele frequency; SE, standard error.

^*^ P-values were calculated in the present study using FastGWA tool for linear mixed model framework.

^†^ P-values were obtained from summary statistics of more than 11 million single nucleotide polymorphisms for processed meat intake frequency in previous study. Summary statistic data can be downloaded at <https://personal.broadinstitute.org/ryank/Cole_UKB_Diet_GWAS.tar>. Source: Cole JB, Florez JC, Hirschhorn JN. Comprehensive genomic analysis of dietary habits in UK Biobank identifies hundreds of genetic associations. Nat Commun. 2020;11(1):1467.

^‡^ Loci were either not available in Cole et al.’s study or not reach significant level of 5e-08.

**Summary statistics of genomic risk loci identified from genome-wide association analysis of poultry consumption**

| **Chr** | **Variant** | **Position** | **Reference allele** | **Alternative allele** | **RAF** | **Beta** | **SE** | **P_FastGWA_^*^** | **P_poultry_^†^** |
| --- | --- | --- | --- | --- | --- | --- | --- | --- | --- |
| 16 | rs34473833 ^‡^ | 64876684 | A | G | 0.984 | 0.0588 | 0.0108 | 4.61e-08 | 1.30e-05 |

Chr, chromosome; RAF, reference allele frequency; SE, standard error.

^*^ P-values were calculated in the present study using FastGWA tool for linear mixed model framework.

^†^ P-values were obtained from summary statistics of more than 11 million single nucleotide polymorphisms for overall poultry intake frequency in previous study. Summary statistic data can be downloaded at <https://personal.broadinstitute.org/ryank/Cole_UKB_Diet_GWAS.tar>. Source: Cole JB, Florez JC, Hirschhorn JN. Comprehensive genomic analysis of dietary habits in UK Biobank identifies hundreds of genetic associations. Nat Commun. 2020;11(1):1467.

^‡^ Loci was not reach significant level of 5e-08.

**Summary statistics of genomic risk loci identified from genome-wide association analysis of fish consumption**

| **Chr** | **Variant** | **Position** | **Reference allele** | **Alternative allele** | **RAF** | **Beta** | **SE** | **P_FastGWA_^*^** | **P_oily fish_^†^** | **P_non-oily fish_^†^** |
| --- | --- | --- | --- | --- | --- | --- | --- | --- | --- | --- |
| 1 | rs1460943 ^‡^ | 72813129 | C | T | 0.400 | -0.0196 | 0.0035 | 2.31e-08 | 6.40e-08 | 1.30e-03 |
| 1 | rs12566777 ^‡^ | 112590831 | T | C | 0.651 | 0.0214 | 0.0036 | 3.85e-09 | 5.10e-06 | 9.90e-03 |
| 2 | rs4600686 ^‡^ | 79145715 | T | C | 0.837 | 0.0259 | 0.0047 | 2.96e-08 | 9.80e-04 | 6.20e-05 |
| 3 | rs6577598 ^‡^ | 17885531 | T | A | 0.520 | -0.0212 | 0.0035 | 1.17e-09 | 7.70e-07 | 1.40e-05 |
| 3 | rs12630658 ^‡^ | 25122238 | T | C | 0.420 | -0.0203 | 0.0035 | 6.46e-09 | 6.80e-07 | 8.80e-06 |
| 3 | 3:114916925 ^‡^ | 114916925 | CT | C | 0.300 | 0.0206 | 0.0038 | 4.98e-08 | NA | NA |
| 5 | rs469250 ^‡^ | 102140615 | G | C | 0.367 | 0.0195 | 0.0036 | 4.48e-08 | 6.70e-07 | 2.70e-03 |
| 6 | rs3734543 | 26468545 | G | C | 0.878 | 0.0370 | 0.0053 | 1.81e-12 | 5.60e-15 | 1.10e-08 |
| 7 | rs11767283 | 121947456 | A | G | 0.779 | -0.0228 | 0.0042 | 4.99e-08 | 6.20e-14 | 6.10e-02 |
| 8 | 8:8661026 ^‡^ | 8661026 | CA | C | 0.512 | -0.0220 | 0.0035 | 2.65e-10 | NA | NA |
| 8 | rs9650651 | 10267540 | C | A | 0.589 | -0.0229 | 0.0035 | 6.49e-11 | 5.00e-13 | 1.10e-04 |
| 8 | rs5891918 | 64603887 | G | GT | 0.278 | -0.0236 | 0.0039 | 1.14e-09 | 1.30e-12 | 5.00e-06 |
| 10 | rs12251016 | 21821918 | A | T | 0.657 | 0.0244 | 0.0036 | 1.68e-11 | 3.00e-19 | 2.50e-04 |
| 11 | rs535669 ^‡^ | 28729030 | A | G | 0.691 | 0.0203 | 0.0037 | 4.99e-08 | 7.50e-07 | 1.30e-05 |
| 12 | rs35287743 | 110057250 | G | T | 0.883 | 0.0476 | 0.0054 | 1.17e-18 | 1.90e-19 | 3.30e-10 |
| 13 | rs7336980 | 59437165 | C | G | 0.724 | 0.0231 | 0.0039 | 2.16e-09 | 4.20e-11 | 7.00e-06 |
| 14 | rs7146955 | 29750244 | A | G | 0.410 | 0.0221 | 0.0035 | 2.66e-10 | 6.40e-14 | 5.40e-08 |
| 14 | rs200738790 ^‡^ | 80721903 | G | GA | 0.999 | 0.4094 | 0.0745 | 3.93e-08 | NA | NA |
| 14 | rs12887132 ^‡^ | 100284679 | C | G | 0.595 | 0.0196 | 0.0035 | 2.41e-08 | 1.20e-06 | 2.90e-06 |
| 16 | rs9889161 | 51495068 | G | T | 0.642 | 0.0218 | 0.0036 | 1.49e-09 | 1.70e-11 | 7.30e-05 |
| 16 | rs7187250 | 53810546 | C | A | 0.607 | -0.0261 | 0.0035 | 1.35e-13 | 1.30e-17 | 2.70e-12 |
| 16 | rs11859365 | 83683945 | A | C | 0.747 | -0.0253 | 0.0040 | 1.64e-10 | 2.60e-23 | 3.40e-06 |
| 18 | rs7238896 | 1840658 | A | G | 0.859 | -0.0297 | 0.0050 | 2.31e-09 | 8.40e-09 | 1.40e-04 |
| 18 | rs7240986 | 53195249 | G | A | 0.630 | -0.0207 | 0.0036 | 7.40e-09 | 6.50e-09 | 3.10e-03 |
| 18 | rs242641 | 58853208 | C | T | 0.699 | 0.0214 | 0.0038 | 1.29e-08 | 7.30e-09 | 3.10e-06 |
| 19 | rs429358 | 45411941 | T | C | 0.844 | -0.0380 | 0.0047 | 1.16e-15 | 1.20e-11 | 4.00e-07 |
| 19 | rs8103840 | 49254955 | C | T | 0.531 | -0.0278 | 0.0035 | 1.34e-15 | 5.70e-18 | 1.10e-19 |
| 22 | 22:31871253 ^‡^ | 31871253 | GA | G | 0.687 | -0.0205 | 0.0038 | 4.83e-08 | NA | NA |

Chr, chromosome; RAF, reference allele frequency; SE, standard error; NA, not available.

^*^ P-values were calculated in the present study using FastGWA tool for linear mixed model framework.

^†^ P-values were obtained from summary statistics of more than 11 million single nucleotide polymorphisms for overall oily and non-oily fish intake in previous study. Summary statistic data can be downloaded at <https://personal.broadinstitute.org/ryank/Cole_UKB_Diet_GWAS.tar>. Source: Cole JB, Florez JC, Hirschhorn JN. Comprehensive genomic analysis of dietary habits in UK Biobank identifies hundreds of genetic associations. Nat Commun. 2020;11(1):1467.

^‡^ Novel loci were determined as either not available in Cole et al.’s study or not reach significant level of 5e-08.

**Summary statistics of genomic risk loci identified from genome-wide association analysis of milk consumption**

| **Chr** | **Variant** | **Position** | **Reference allele** | **Alternative allele** | **RAF** | **Beta** | **SE** | **P_FastGWA_^*^** | **P_full cream_^†^** | **P_semi-skimmed_^†^** | **P_skimmed_^†^** |
| --- | --- | --- | --- | --- | --- | --- | --- | --- | --- | --- | --- |
| 1 | rs201406724 | 150781915 | T | TA | 0.652 | -0.0166 | 0.0024 | 5.41e-12 | 0.31 | 0.61 | 0.91 |
| 1 | rs11264235 | 154631081 | C | T | 0.720 | -0.0157 | 0.0025 | 7.03e-10 | 0.61 | 0.53 | 0.9 |
| 1 | rs2901785 | 174104743 | G | A | 0.552 | 0.0184 | 0.0023 | 9.65e-16 | 0.61 | 0.13 | 0.91 |
| 2 | 2:27748992 | 27748992 | AT | A | 0.381 | -0.0162 | 0.0024 | 6.51e-12 | NA | NA | NA |
| 2 | rs183195584 | 48345073 | T | A | 0.992 | 0.0751 | 0.0133 | 1.55e-08 | 0.027 | 0.05 | 0.035 |
| 2 | 2:58364214 | 58364214 | CAT | C | 0.731 | 0.0148 | 0.0026 | 9.98e-09 | NA | NA | NA |
| 2 | rs4611605 | 157112515 | A | T | 0.740 | -0.0150 | 0.0026 | 1.06e-08 | 0.037 | 0.054 | 0.012 |
| 3 | rs13082065 | 35731993 | C | T | 0.588 | -0.0129 | 0.0023 | 2.77e-08 | 0.99 | 0.58 | 0.42 |
| 3 | rs57462170 | 50239803 | G | A | 0.891 | -0.0205 | 0.0037 | 2.06e-08 | 0.003 | 0.0043 | 0.031 |
| 4 | rs11940694 | 39414993 | A | G | 0.394 | 0.0150 | 0.0023 | 1.84e-10 | 0.25 | 0.024 | 0.0024 |
| 4 | rs6819372 | 67970101 | A | G | 0.468 | 0.0130 | 0.0023 | 1.33e-08 | 0.24 | 0.8 | 0.33 |
| 4 | rs2199936 | 89045331 | A | G | 0.114 | -0.0405 | 0.0036 | 9.59e-30 | 0.29 | 0.37 | 0.15 |
| 5 | rs7730403 | 7429254 | G | A | 0.564 | 0.0138 | 0.0023 | 1.79e-09 | 0.051 | 0.028 | 0.13 |
| 5 | rs12518404 | 60341996 | T | C | 0.569 | -0.0148 | 0.0023 | 1.24e-10 | 0.53 | 0.23 | 0.19 |
| 5 | rs12658032 | 103904226 | A | G | 0.356 | -0.0134 | 0.0024 | 1.56e-08 | 0.75 | 0.35 | 0.72 |
| 5 | 5:124728469 | 124728469 | AG | A | 0.636 | -0.0138 | 0.0024 | 6.40e-09 | NA | NA | NA |
| 5 | rs35124713 | 151951108 | G | A | 0.727 | 0.0150 | 0.0026 | 4.45e-09 | 0.84 | 0.86 | 0.88 |
| 6 | rs2465018 | 51241140 | G | A | 0.769 | -0.0189 | 0.0027 | 3.56e-12 | 0.68 | 0.16 | 0.96 |
| 6 | rs9490002 | 98331134 | G | A | 0.540 | -0.0145 | 0.0023 | 2.54e-10 | 0.19 | 0.064 | 0.16 |
| 6 | rs1101559 | 127047683 | C | T | 0.517 | 0.0125 | 0.0023 | 3.99e-08 | 0.25 | 0.74 | 0.89 |
| 6 | rs139797380 | 137244957 | C | G | 0.992 | 0.0977 | 0.0133 | 1.93e-13 | 0.13 | 0.44 | 0.23 |
| 7 | rs4410790 | 17284577 | T | C | 0.365 | -0.0645 | 0.0024 | 4.72e-164 | 0.29 | 0.54 | 0.16 |
| 7 | rs1476765 | 32320458 | G | T | 0.379 | 0.0130 | 0.0024 | 4.92e-08 | 0.53 | 0.27 | 0.41 |
| 7 | 7:73042302 | 73042302 | GCTTT | G | 0.867 | -0.0356 | 0.0034 | 2.57e-26 | NA | NA | NA |
| 7 | rs17685 | 75616105 | G | A | 0.722 | -0.0345 | 0.0025 | 3.45e-42 | 0.041 | 0.36 | 0.4 |
| 8 | 8:73433232 | 73433232 | GGTA | G | 0.299 | -0.0137 | 0.0025 | 4.31e-08 | NA | NA | NA |
| 8 | rs2737216 | 116630079 | A | T | 0.439 | 0.0137 | 0.0023 | 3.26e-09 | 0.028 | 0.0066 | 0.0012 |
| 10 | 10:87339574 | 87339574 | CA | C | 0.434 | 0.0135 | 0.0023 | 4.36e-09 | NA | NA | NA |
| 10 | rs10786069 | 94845546 | C | T | 0.470 | 0.0157 | 0.0023 | 8.18e-12 | 0.091 | 0.0013 | 0.00096 |
| 10 | rs12256016 | 131476007 | A | G | 0.737 | -0.0159 | 0.0026 | 1.01e-09 | 0.39 | 0.054 | 0.031 |
| 11 | rs71480157 | 27610041 | T | C | 0.817 | 0.0186 | 0.0030 | 3.91e-10 | 0.79 | 0.41 | 0.56 |
| 11 | rs78059714 | 46157568 | C | T | 0.823 | -0.0166 | 0.0030 | 2.87e-08 | 0.004 | 0.0086 | 0.028 |
| 12 | 12:111854285 | 111854285 | GT | G | 0.797 | -0.0184 | 0.0029 | 1.40e-10 | NA | NA | NA |
| 12 | rs7957424 | 120894318 | G | A | 0.442 | 0.0129 | 0.0023 | 1.81e-08 | 0.0099 | 0.00019 | 0.011 |
| 13 | rs2937338 | 55999207 | A | G | 0.265 | 0.0148 | 0.0026 | 8.38e-09 | 0.026 | 0.0009 | 0.0068 |
| 14 | rs71101691 | 29716334 | C | CT | 0.460 | -0.0126 | 0.0023 | 4.35e-08 | 0.076 | 0.1 | 0.021 |
| 14 | rs4509952 | 98869431 | G | A | 0.399 | -0.0128 | 0.0023 | 3.92e-08 | 0.64 | 0.94 | 0.81 |
| 15 | rs12591786 | 60902512 | C | T | 0.842 | 0.0203 | 0.0032 | 1.57e-10 | 0.16 | 0.58 | 0.59 |
| 15 | rs12909335 | 75214789 | T | G | 0.439 | -0.0386 | 0.0023 | 1.71e-63 | 0.97 | 0.93 | 0.76 |
| 16 | 16:18840320 | 18840320 | CA | C | 0.602 | 0.0158 | 0.0023 | 1.60e-11 | NA | NA | NA |
| 16 | rs378421 | 28754684 | G | A | 0.582 | -0.0136 | 0.0023 | 4.62e-09 | 0.82 | 0.31 | 0.028 |
| 16 | rs62039910 | 64694053 | G | A | 0.919 | 0.0231 | 0.0042 | 3.56e-08 | 0.37 | 0.00027 | 0.00096 |
| 17 | rs12600469 | 40834073 | G | T | 0.376 | -0.0157 | 0.0024 | 2.28e-11 | 0.036 | 0.087 | 0.25 |
| 18 | 18:57835314 | 57835314 | CT | C | 0.676 | -0.0155 | 0.0025 | 2.58e-10 | NA | NA | NA |
| 19 | rs11555274 | 13207284 | G | C | 0.884 | -0.0203 | 0.0035 | 9.78e-09 | 0.13 | 0.052 | 0.35 |
| 19 | rs3764567 | 19440066 | C | T | 0.343 | 0.0177 | 0.0024 | 1.61e-13 | 0.023 | 0.011 | 0.1 |
| 19 | rs12459249 | 41339896 | T | C | 0.333 | -0.0149 | 0.0024 | 7.82e-10 | 0.062 | 0.43 | 0.64 |
| 20 | rs6033239 | 11845930 | G | T | 0.635 | 0.0139 | 0.0024 | 3.91e-09 | 0.52 | 0.89 | 0.55 |
| 20 | rs6129077 | 59791342 | C | G | 0.324 | -0.0135 | 0.0024 | 2.79e-08 | 0.82 | 0.26 | 0.051 |
| 20 | rs73147887 | 62890294 | C | G | 0.775 | -0.0241 | 0.0028 | 2.97e-18 | 0.029 | 0.00087 | 0.01 |

Chr, chromosome; RAF, reference allele frequency; SE, standard error; NA, not available.

^*^ P-values were calculated in the present study using FastGWA tool for linear mixed model framework.

^†^ P-values were obtained from summary statistics of more than 11 million single nucleotide polymorphisms for milk type intake (full cream vs. never, semi-skimmed vs. never, skimmed vs. never, soy milk vs. never) in previous study. Summary statistic data can be downloaded at <https://personal.broadinstitute.org/ryank/Cole_UKB_Diet_GWAS.tar>. Source: Cole JB, Florez JC, Hirschhorn JN. Comprehensive genomic analysis of dietary habits in UK Biobank identifies hundreds of genetic associations. Nat Commun. 2020;11(1):1467.

All loci were either not available in Cole et al.’s study or not reach significant level of 5e-08.

**Summary statistics of genomic risk loci identified from genome-wide association analysis of cheese consumption**

| **Chr** | **Variant** | **Position** | **Reference allele** | **Alternative allele** | **RAF** | **Beta** | **SE** | **P_FastGWA_^*^** | **P_cheese_^†^** |
| --- | --- | --- | --- | --- | --- | --- | --- | --- | --- |
| 1 | rs7513705 ^‡^ | 93666349 | T | C | 0.350 | -0.0242 | 0.0040 | 2.05e-09 | 4.70e-06 |
| 1 | rs6685323 ^‡^ | 154295592 | C | T | 0.690 | 0.0233 | 0.0042 | 2.35e-08 | 5.80e-06 |
| 2 | rs72790304 | 24108557 | G | A | 0.826 | 0.0319 | 0.0051 | 3.69e-10 | 1.10e-09 |
| 2 | rs543059375 ^‡^ | 43910835 | C | A | 0.999 | 0.5276 | 0.0965 | 4.53e-08 | NA |
| 2 | rs549814 | 45153508 | C | T | 0.672 | -0.0440 | 0.0041 | 1.82e-26 | 2.10e-32 |
| 2 | rs12475594 ^‡^ | 58433375 | A | G | 0.823 | -0.0304 | 0.0051 | 2.09e-09 | 6.20e-06 |
| 2 | rs12472445 ^‡^ | 161309867 | G | C | 0.253 | 0.0246 | 0.0044 | 3.17e-08 | 4.90e-05 |
| 2 | rs1014444 ^‡^ | 162868858 | A | G | 0.673 | -0.0241 | 0.0041 | 5.27e-09 | 2.40e-07 |
| 2 | rs1514755 ^‡^ | 166299635 | A | G | 0.758 | -0.0247 | 0.0045 | 4.65e-08 | 2.40e-07 |
| 3 | rs191087010 ^‡^ | 36713977 | T | C | 0.990 | 0.1124 | 0.0205 | 4.31e-08 | 6.60e-06 |
| 3 | 3:49800212 ^‡^ | 49800212 | CT | C | 0.492 | 0.0302 | 0.0039 | 1.25e-14 | NA |
| 3 | rs62245792 | 68410652 | T | A | 0.849 | 0.0312 | 0.0055 | 1.05e-08 | 2.30e-09 |
| 3 | rs2271054 ^‡^ | 107763040 | G | A | 0.821 | 0.0283 | 0.0051 | 3.19e-08 | 3.40e-06 |
| 3 | rs2054710 ^‡^ | 161090616 | C | T | 0.693 | -0.0232 | 0.0042 | 3.14e-08 | 1.30e-07 |
| 3 | rs79184944 ^‡^ | 161549397 | T | A | 0.865 | -0.0343 | 0.0057 | 1.33e-09 | 6.00e-07 |
| 4 | rs113591949 ^‡^ | 17851945 | A | G | 0.874 | 0.0385 | 0.0059 | 5.48e-11 | 1.50e-07 |
| 4 | rs10938397 | 45182527 | A | G | 0.566 | 0.0244 | 0.0039 | 3.94e-10 | 3.80e-10 |
| 4 | rs11425887 ^‡^ | 80938689 | C | CA | 0.260 | -0.0263 | 0.0044 | 2.16e-09 | 1.70e-06 |
| 4 | rs13107325 | 103188709 | C | T | 0.926 | 0.0466 | 0.0074 | 2.48e-10 | 5.10e-11 |
| 4 | rs4692708 | 170228542 | A | C | 0.747 | -0.0261 | 0.0045 | 5.51e-09 | 1.20e-08 |
| 5 | rs2202268 ^‡^ | 62966917 | T | G | 0.393 | 0.0229 | 0.0040 | 7.59e-09 | 1.60e-06 |
| 5 | rs12522093 ^‡^ | 153603147 | T | A | 0.576 | 0.0216 | 0.0039 | 3.52e-08 | 3.40e-06 |
| 6 | rs975303 | 19028788 | A | G | 0.817 | -0.0311 | 0.0050 | 4.96e-10 | 6.30e-11 |
| 6 | rs75397441 ^‡^ | 26022392 | C | T | 0.910 | -0.0380 | 0.0067 | 1.65e-08 | 1.30e-06 |
| 6 | rs62412526 ^‡^ | 56553969 | G | T | 0.768 | 0.0262 | 0.0046 | 9.37e-09 | 5.00e-08 |
| 7 | 7:2206114 ^‡^ | 2206114 | TA | T | 0.776 | 0.0299 | 0.0046 | 1.15e-10 | NA |
| 7 | 7:18152033 ^‡^ | 18152033 | CA | C | 0.806 | 0.0282 | 0.0049 | 9.95e-09 | NA |
| 7 | rs12672200 | 115461436 | G | A | 0.675 | 0.0238 | 0.0041 | 8.28e-09 | 4.40e-08 |
| 7 | rs56182580 | 132691858 | T | C | 0.717 | 0.0257 | 0.0043 | 2.26e-09 | 1.10e-09 |
| 7 | rs60198071 | 140144929 | A | G | 0.726 | -0.0247 | 0.0044 | 1.60e-08 | 6.00e-09 |
| 8 | rs7012814 | 9173358 | G | A | 0.525 | 0.0307 | 0.0039 | 2.98e-15 | 5.40e-14 |
| 8 | rs77883185 ^‡^ | 85620323 | C | T | 0.946 | 0.0483 | 0.0087 | 2.72e-08 | 3.80e-06 |
| 9 | rs7047365 ^‡^ | 124630333 | T | C | 0.512 | -0.0212 | 0.0039 | 3.71e-08 | 4.40e-07 |
| 11 | 11:43623050 ^‡^ | 43623050 | CTTTT | C | 0.386 | -0.0234 | 0.0040 | 4.29e-09 | NA |
| 11 | rs17854357 | 65601560 | G | C | 0.833 | 0.0311 | 0.0052 | 1.80e-09 | 7.10e-11 |
| 11 | 11:77297159 ^‡^ | 77297159 | CTGAG | C | 0.919 | 0.0456 | 0.0072 | 2.48e-10 | NA |
| 11 | rs11604424 | 116651115 | C | T | 0.201 | -0.0267 | 0.0048 | 2.93e-08 | 2.00e-08 |
| 12 | rs4963739 ^‡^ | 24191827 | T | C | 0.497 | 0.0213 | 0.0039 | 3.64e-08 | 9.20e-07 |
| 12 | rs112890481 | 49859149 | C | CT | 0.820 | -0.0299 | 0.0050 | 3.08e-09 | 2.90e-10 |
| 12 | rs1024853 | 107291383 | C | G | 0.563 | 0.0222 | 0.0039 | 1.40e-08 | 7.80e-09 |
| 12 | rs61953351 ^‡^ | 121456616 | G | T | 0.750 | -0.0245 | 0.0045 | 3.66e-08 | 5.20e-07 |
| 12 | rs7299943 ^‡^ | 123593485 | T | A | 0.802 | -0.0273 | 0.0049 | 1.85e-08 | 3.80e-07 |
| 13 | 13:58354282 ^‡^ | 58354282 | TA | T | 0.267 | -0.0300 | 0.0044 | 7.84e-12 | NA |
| 13 | rs34190997 ^‡^ | 59350679 | G | GTA | 0.883 | -0.0350 | 0.0061 | 1.20e-08 | 4.40e-06 |
| 13 | rs4886168 ^‡^ | 60073056 | T | C | 0.837 | 0.0291 | 0.0053 | 3.65e-08 | 2.30e-05 |
| 13 | rs116066016 ^‡^ | 66293017 | A | C | 1.000 | -0.6402 | 0.1160 | 3.44e-08 | NA |
| 14 | rs10143659 ^‡^ | 30145432 | A | T | 0.584 | 0.0228 | 0.0039 | 6.74e-09 | 1.10e-07 |
| 15 | rs1473781 ^‡^ | 41818917 | G | A | 0.655 | 0.0239 | 0.0041 | 4.36e-09 | 3.80e-07 |
| 16 | rs76632611 | 7742888 | C | T | 0.867 | -0.0314 | 0.0057 | 4.45e-08 | 1.10e-08 |
| 16 | rs276950 ^‡^ | 86243437 | T | C | 0.847 | 0.0296 | 0.0054 | 4.17e-08 | 7.40e-05 |
| 17 | rs684214 | 40696915 | C | T | 0.717 | 0.0266 | 0.0043 | 5.53e-10 | 2.70e-08 |
| 17 | rs79475968 | 44326895 | T | C | 0.812 | 0.0339 | 0.0050 | 1.24e-11 | 1.70e-12 |
| 17 | rs919109 ^‡^ | 46675977 | G | C | 0.861 | -0.0343 | 0.0056 | 8.94e-10 | 1.20e-06 |
| 17 | 17:62009101 ^‡^ | 62009101 | CTT | C | 0.746 | -0.0300 | 0.0044 | 1.45e-11 | NA |
| 18 | rs2960578 | 21143739 | T | G | 0.501 | -0.0271 | 0.0039 | 2.27e-12 | 5.60e-17 |
| 18 | rs7237482 ^‡^ | 44317079 | A | T | 0.577 | -0.0241 | 0.0039 | 7.36e-10 | 4.30e-07 |
| 20 | rs6029941 | 35519475 | G | A | 0.456 | 0.0271 | 0.0039 | 2.89e-12 | 1.10e-11 |
| 22 | rs35821760 ^‡^ | 32398185 | A | T | 0.733 | 0.0243 | 0.0044 | 2.91e-08 | 1.30e-06 |
| 22 | rs28741121 | 42025823 | G | A | 0.829 | -0.0356 | 0.0052 | 5.71e-12 | 6.40e-12 |

Chr, chromosome; RAF, reference allele frequency; SE, standard error; NA, not available.

^*^ P-values were calculated in the present study using FastGWA tool for linear mixed model framework.

^†^ P-values were obtained from summary statistics of more than 11 million single nucleotide polymorphisms for overall cheese intake in previous study. Summary statistic data can be downloaded at <https://personal.broadinstitute.org/ryank/Cole_UKB_Diet_GWAS.tar>. Source: Cole JB, Florez JC, Hirschhorn JN. Comprehensive genomic analysis of dietary habits in UK Biobank identifies hundreds of genetic associations. Nat Commun. 2020;11(1):1467.

^‡^ Loci were either not available in Cole et al.’s study or not reach significant level of 5e-08.

**Summary statistics of genomic risk loci identified from genome-wide association analysis of fruit consumption**

| **Chr** | **Variant** | **Position** | **Reference allele** | **Alternative allele** | **RAF** | **Beta** | **SE** | **P_FastGWA_^*^** | **P_fresh fruit_^†^** | **P_dried fruit_^†^** |
| --- | --- | --- | --- | --- | --- | --- | --- | --- | --- | --- |
| 1 | rs144200595 ^‡^ | 17859305 | G | A | 1.000 | -1.7369 | 0.2810 | 6.37e-10 | NA | NA |
| 1 | 1:26199000 ^‡^ | 26199000 | ATT | A | 0.450 | 0.0227 | 0.0042 | 4.64e-08 | NA | NA |
| 1 | rs1620977 | 72729142 | A | G | 0.267 | 0.0413 | 0.0047 | 7.53e-19 | 3.00e-25 | 2.90e-12 |
| 1 | rs61421373 | 204560440 | C | T | 0.779 | -0.0284 | 0.0050 | 1.14e-08 | 4.10e-10 | 3.60e-06 |
| 1 | rs112087954 ^‡^ | 205179021 | C | T | 1.000 | -2.2062 | 0.3933 | 2.03e-08 | NA | NA |
| 2 | rs10188334 | 653874 | C | T | 0.829 | 0.0401 | 0.0055 | 3.02e-13 | 5.50e-13 | 1.80e-03 |
| 2 | rs535987588 ^‡^ | 16794221 | G | A | 1.000 | -0.9733 | 0.1334 | 2.99e-13 | NA | NA |
| 2 | rs4953150 | 45157336 | C | T | 0.657 | 0.0238 | 0.0044 | 4.71e-08 | 7.60e-17 | 6.20e-01 |
| 2 | rs12477242 | 58452792 | C | G | 0.661 | -0.0260 | 0.0044 | 2.39e-09 | 4.70e-08 | 3.10e-02 |
| 2 | rs6731782 | 60224329 | A | T | 0.456 | 0.0262 | 0.0041 | 2.16e-10 | 1.10e-11 | 3.00e-04 |
| 2 | rs2710641 | 63149265 | G | A | 0.634 | -0.0240 | 0.0043 | 1.85e-08 | 3.00e-08 | 9.30e-01 |
| 2 | rs576565334 ^‡^ | 102164766 | T | A | 1.000 | -1.6073 | 0.2556 | 3.22e-10 | NA | NA |
| 2 | rs111915841 | 225467840 | G | C | 0.679 | -0.0252 | 0.0044 | 1.21e-08 | 5.60e-10 | 5.90e-06 |
| 2 | rs568572432 ^‡^ | 232572795 | C | T | 1.000 | -0.8833 | 0.1617 | 4.72e-08 | NA | NA |
| 3 | rs13069655 | 21025726 | G | C | 0.495 | -0.0228 | 0.0041 | 3.49e-08 | 3.90e-13 | 9.20e-02 |
| 3 | rs10490869 | 35635145 | A | T | 0.789 | 0.0309 | 0.0051 | 1.09e-09 | 1.10e-08 | 3.70e-06 |
| 3 | rs12637791 | 85525323 | T | G | 0.352 | 0.0238 | 0.0043 | 3.02e-08 | 2.50e-11 | 1.60e-01 |
| 3 | rs7647305 | 185834290 | T | C | 0.214 | -0.0279 | 0.0050 | 2.92e-08 | 3.90e-10 | 2.20e-02 |
| 4 | rs547606538 ^‡^ | 7704769 | A | G | 0.998 | -0.2716 | 0.0496 | 4.36e-08 | NA | NA |
| 4 | rs357842 | 60004974 | A | G | 0.660 | 0.0266 | 0.0044 | 1.09e-09 | 4.10e-11 | 4.90e-04 |
| 4 | rs183649060 ^‡^ | 69697924 | A | T | 1.000 | -1.0953 | 0.1969 | 2.64e-08 | NA | NA |
| 4 | rs140675951 ^‡^ | 108300048 | T | A | 1.000 | -1.1749 | 0.2138 | 3.89e-08 | NA | NA |
| 5 | rs6864095 | 60545515 | G | T | 0.520 | -0.0251 | 0.0041 | 1.19e-09 | 3.70e-11 | 3.30e-05 |
| 5 | rs114489117 | 92948485 | T | A | 0.893 | -0.0377 | 0.0067 | 1.48e-08 | 6.20e-10 | 5.40e-02 |
| 5 | rs10070258 | 93980014 | T | G | 0.693 | 0.0270 | 0.0045 | 1.35e-09 | 6.90e-10 | 1.80e-01 |
| 6 | rs764511845 | 51215155 | TAAAAG  AGTAAA  AGAAAA  G | T | 0.765 | -0.0401 | 0.0049 | 1.66e-16 | 7.70e-24 | 2.20e-02 |
| 6 | rs764511845 | 51215155 | TAAAAG  AGTAAA  AGAAAA  GAA | T | 0.769 | -0.0398 | 0.0049 | 5.74e-16 | 7.70e-24 | 2.20e-02 |
| 6 | rs149365686 | 98783306 | T | TTCTC | 0.487 | 0.0278 | 0.0041 | 1.53e-11 | 1.70e-09 | 6.20e-06 |
| 6 | rs561713124 ^‡^ | 104498086 | G | A | 1.000 | -0.9364 | 0.1653 | 1.48e-08 | NA | NA |
| 6 | rs539376176 ^‡^ | 122169947 | A | C | 1.000 | -0.8433 | 0.1451 | 6.18e-09 | NA | NA |
| 6 | rs183000662 ^‡^ | 129167476 | C | T | 1.000 | -1.1382 | 0.1753 | 8.34e-11 | NA | NA |
| 6 | rs559782145 ^‡^ | 134001863 | C | G | 1.000 | -1.2798 | 0.2056 | 4.79e-10 | NA | NA |
| 7 | rs587612927 ^‡^ | 74035427 | C | T | 1.000 | -0.8564 | 0.1503 | 1.22e-08 | NA | NA |
| 7 | rs572190897 ^‡^ | 99186455 | A | C | 1.000 | -1.6565 | 0.2959 | 2.17e-08 | NA | NA |
| 7 | rs34475839 | 133622287 | C | A | 0.823 | 0.0323 | 0.0054 | 2.36e-09 | 8.50e-10 | 2.40e-05 |
| 7 | rs6967154 | 143713124 | A | T | 0.627 | -0.0591 | 0.0042 | 4.93e-44 | 7.50e-64 | 9.80e-05 |
| 7 | rs2533200 | 153489074 | C | G | 0.514 | 0.0239 | 0.0041 | 8.08e-09 | 6.60e-09 | 2.00e-09 |
| 8 | rs531625906 | 10197291 | T | TC | 0.768 | 0.0320 | 0.0049 | 8.42e-11 | 1.80e-13 | 1.90e-02 |
| 8 | rs7005201 ^‡^ | 59901813 | G | T | 0.302 | -0.0249 | 0.0045 | 2.89e-08 | 7.50e-08 | 3.40e-02 |
| 9 | rs115540638 | 34191992 | A | T | 0.782 | 0.0291 | 0.0050 | 5.31e-09 | 1.50e-12 | 3.70e-03 |
| 9 | rs943617 ^‡^ | 114937852 | A | G | 0.224 | 0.0276 | 0.0049 | 2.44e-08 | 1.20e-05 | 8.80e-06 |
| 9 | rs144105946 ^‡^ | 119148887 | T | G | 1.000 | -1.6412 | 0.2438 | 1.67e-11 | NA | NA |
| 10 | rs10828266 | 22098701 | A | G | 0.283 | -0.0460 | 0.0046 | 9.45e-24 | 1.30e-28 | 2.30e-17 |
| 10 | rs144373510 ^‡^ | 55166107 | A | G | 1.000 | -0.5991 | 0.1072 | 2.32e-08 | NA | NA |
| 10 | rs12245149 | 65321147 | C | A | 0.513 | 0.0233 | 0.0041 | 1.52e-08 | 1.00e-10 | 8.80e-08 |
| 10 | 10:126684759 ^‡^ | 126684759 | GC | G | 0.120 | -0.0411 | 0.0064 | 1.78e-10 | NA | NA |
| 11 | rs10128597 | 8694830 | G | A | 0.726 | -0.0285 | 0.0046 | 8.33e-10 | 2.00e-10 | 6.40e-06 |
| 11 | rs3763874 | 9546587 | G | A | 0.584 | -0.0268 | 0.0042 | 1.38e-10 | 3.90e-08 | 2.30e-02 |
| 11 | rs61877052 ^‡^ | 10074294 | A | C | 0.961 | 0.0591 | 0.0106 | 2.15e-08 | 4.50e-07 | 4.40e-02 |
| 11 | rs11032362 | 33759092 | G | A | 0.910 | -0.0411 | 0.0072 | 9.96e-09 | 3.20e-11 | 2.90e-01 |
| 11 | rs569920322 ^‡^ | 72856001 | A | G | 1.000 | -0.9253 | 0.1648 | 1.96e-08 | NA | NA |
| 11 | rs563891191 ^‡^ | 98324748 | C | T | 1.000 | -1.3394 | 0.2328 | 8.74e-09 | NA | NA |
| 12 | rs546444490 ^‡^ | 18725611 | C | A | 1.000 | -0.9026 | 0.1508 | 2.18e-09 | NA | NA |
| 12 | 12:90604769 ^‡^ | 90604769 | GA | G | 0.269 | 0.0266 | 0.0046 | 1.01e-08 | NA | NA |
| 13 | rs573488836 ^‡^ | 88887863 | A | G | 1.000 | -1.3583 | 0.2278 | 2.48e-09 | NA | NA |
| 14 | rs34162196 | 22038125 | C | T | 0.899 | 0.0593 | 0.0068 | 3.30e-18 | 5.10e-24 | 8.70e-18 |
| 14 | 14:43982395 ^‡^ | 43982395 | TA | T | 1.000 | -1.6765 | 0.3028 | 3.08e-08 | NA | NA |
| 14 | rs2370982 | 79890677 | C | T | 0.785 | -0.0275 | 0.0050 | 4.64e-08 | 2.70e-17 | 8.50e-01 |
| 15 | rs561549058 ^‡^ | 37047924 | G | A | 1.000 | -0.7598 | 0.1335 | 1.24e-08 | NA | NA |
| 15 | rs11073269 ^‡^ | 38120848 | T | A | 0.510 | 0.0227 | 0.0041 | 4.51e-08 | 2.50e-06 | 1.50e-03 |
| 16 | rs140227131 ^‡^ | 5910535 | G | A | 1.000 | -0.6253 | 0.1113 | 1.95e-08 | NA | NA |
| 16 | rs7205037 | 7624265 | G | C | 0.537 | 0.0228 | 0.0041 | 3.36e-08 | 1.90e-09 | 3.80e-02 |
| 16 | 16:19269275 ^‡^ | 19269275 | GT | G | 0.384 | 0.0238 | 0.0043 | 2.57e-08 | NA | NA |
| 16 | rs572940299 ^‡^ | 25421313 | T | C | 1.000 | -1.3339 | 0.2289 | 5.59e-09 | NA | NA |
| 16 | rs112539866 | 30050078 | C | CA | 0.538 | -0.0238 | 0.0042 | 1.05e-08 | 6.60e-10 | 2.60e-05 |
| 16 | rs567194467 ^‡^ | 57467817 | G | T | 1.000 | -1.0095 | 0.1719 | 4.28e-09 | NA | NA |
| 16 | rs2967193 | 64288692 | T | C | 0.424 | 0.0247 | 0.0042 | 3.15e-09 | 2.70e-08 | 6.00e-03 |
| 16 | rs67541953 | 73605538 | A | AT | 0.516 | 0.0246 | 0.0041 | 2.93e-09 | 3.50e-15 | 4.10e-06 |
| 16 | rs531881647 ^‡^ | 77577429 | G | C | 1.000 | -1.2879 | 0.2090 | 7.23e-10 | NA | NA |
| 17 | rs529552269 ^‡^ | 25669886 | T | C | 1.000 | -0.7853 | 0.1366 | 8.97e-09 | NA | NA |
| 18 | rs34156224 ^‡^ | 24588876 | G | GT | 0.600 | 0.0231 | 0.0042 | 4.69e-08 | 1.70e-05 | 1.00e-04 |
| 18 | rs949017 | 44814404 | A | G | 0.444 | -0.0288 | 0.0042 | 5.57e-12 | 6.90e-17 | 1.20e-01 |
| 18 | rs17773329 | 57944239 | A | G | 0.688 | -0.0367 | 0.0045 | 1.70e-16 | 1.30e-15 | 6.50e-04 |
| 18 | rs527897590 ^‡^ | 65038135 | T | G | 1.000 | -1.0321 | 0.1694 | 1.10e-09 | NA | NA |
| 19 | rs555698994 ^‡^ | 3068897 | A | T | 1.000 | -1.1680 | 0.1881 | 5.33e-10 | NA | NA |
| 19 | rs429358 | 45411941 | T | C | 0.844 | -0.0484 | 0.0057 | 1.27e-17 | 6.70e-15 | 1.30e-31 |
| 19 | rs2302593 | 46196634 | C | G | 0.513 | -0.0255 | 0.0041 | 5.61e-10 | 3.20e-15 | 1.90e-04 |
| 19 | rs8103840 | 49254955 | C | T | 0.531 | 0.0230 | 0.0042 | 3.04e-08 | 3.20e-11 | 2.80e-01 |
| 20 | rs78580419 ^‡^ | 42107712 | C | T | 1.000 | -1.4935 | 0.2574 | 6.58e-09 | NA | NA |
| 20 | rs2425835 | 44901836 | A | G | 0.547 | -0.0237 | 0.0041 | 1.04e-08 | 2.70e-08 | 5.20e-02 |
| 20 | rs549171695 ^‡^ | 52300532 | C | T | 1.000 | -1.0752 | 0.1925 | 2.33e-08 | NA | NA |
| 22 | rs6006228 ^‡^ | 30112070 | C | G | 0.575 | 0.0235 | 0.0042 | 1.69e-08 | 1.70e-07 | 7.20e-04 |

Chr, chromosome; RAF, reference allele frequency; SE, standard error; NA, not available.

^*^ P-values were calculated in the present study using FastGWA tool for linear mixed model framework.

^†^ P-values were obtained from summary statistics of more than 11 million single nucleotide polymorphisms for pieces of fresh and dried fruit intake in previous study. Summary statistic data can be downloaded at <https://personal.broadinstitute.org/ryank/Cole_UKB_Diet_GWAS.tar>. Source: Cole JB, Florez JC, Hirschhorn JN. Comprehensive genomic analysis of dietary habits in UK Biobank identifies hundreds of genetic associations. Nat Commun. 2020;11(1):1467.

^‡^ Loci were either not available in Cole et al.’s study or not reach significant level of 5e-08.

**Summary statistics of genomic risk loci identified from genome-wide association analysis of vegetable consumption**

| **Chr** | **Variant** | **Position** | **Reference allele** | **Alternative allele** | **RAF** | **Beta** | **SE** | **P_FastGWA_^*^** | **P_cooked vegetable_^†^** | **P_raw vegetable_^†^** |
| --- | --- | --- | --- | --- | --- | --- | --- | --- | --- | --- |
| 1 | rs531494375 ^‡^ | 11461163 | C | T | 1.000 | -1.8276 | 0.3015 | 1.34e-09 | NA | NA |
| 1 | rs372842590 ^‡^ | 91241265 | G | A | 1.000 | -1.2682 | 0.2265 | 2.14e-08 | NA | NA |
| 1 | 1:153761809 ^‡^ | 153761809 | AT | A | 0.441 | 0.0498 | 0.0069 | 5.62e-13 | NA | NA |
| 2 | rs12714415 | 651430 | T | C | 0.838 | 0.0619 | 0.0093 | 3.42e-11 | 1.60e-04 | 2.80e-09 |
| 2 | rs548801702 ^‡^ | 4646677 | G | A | 1.000 | -1.5982 | 0.2862 | 2.35e-08 | NA | NA |
| 2 | rs564723867 ^‡^ | 20714286 | C | T | 1.000 | -1.2336 | 0.2221 | 2.78e-08 | NA | NA |
| 2 | rs1963398 | 79706878 | A | G | 0.327 | 0.0447 | 0.0072 | 5.51e-10 | 1.20e-10 | 1.00e-08 |
| 2 | rs552320005 ^‡^ | 134226620 | C | A | 0.999 | -1.0479 | 0.1822 | 8.86e-09 | NA | NA |
| 3 | rs570287640 ^‡^ | 7801663 | G | T | 1.000 | -4.9220 | 0.7238 | 1.05e-11 | NA | NA |
| 3 | rs7619139 | 25110415 | T | A | 0.411 | -0.0562 | 0.0069 | 3.72e-16 | 3.50e-12 | 7.00e-22 |
| 3 | rs140084370 ^‡^ | 109434812 | C | G | 1.000 | -1.9844 | 0.3445 | 8.37e-09 | NA | NA |
| 3 | rs150482236 ^‡^ | 116476055 | C | T | 1.000 | -1.3716 | 0.2378 | 8.02e-09 | NA | NA |
| 3 | rs532503391 ^‡^ | 117675492 | G | A | 1.000 | -1.1961 | 0.2129 | 1.94e-08 | NA | NA |
| 3 | rs527623148 ^‡^ | 122761126 | T | C | 1.000 | -1.5570 | 0.2380 | 6.08e-11 | NA | NA |
| 3 | rs558817978 ^‡^ | 148472234 | G | C | 1.000 | -1.7385 | 0.2689 | 1.01e-10 | NA | NA |
| 3 | rs9822804 ^‡^ | 186159741 | G | T | 1.000 | -2.8318 | 0.4540 | 4.44e-10 | NA | NA |
| 4 | rs13115614 ^‡^ | 136488540 | A | G | 0.787 | -0.0471 | 0.0083 | 1.42e-08 | 9.30e-07 | 2.30e-06 |
| 5 | rs562634338 ^‡^ | 4691055 | A | C | 1.000 | -1.2364 | 0.2107 | 4.38e-09 | NA | NA |
| 7 | rs139549768 ^‡^ | 54932540 | A | T | 1.000 | -3.7828 | 0.4597 | 1.89e-16 | NA | NA |
| 7 | rs145929636 ^‡^ | 57391143 | C | G | 1.000 | -4.0848 | 0.5178 | 3.04e-15 | NA | NA |
| 7 | rs140838198 ^‡^ | 64802985 | C | G | 1.000 | -2.3789 | 0.4108 | 7.01e-09 | NA | NA |
| 7 | rs139017236 ^‡^ | 67258155 | T | C | 1.000 | -2.5547 | 0.3999 | 1.67e-10 | NA | NA |
| 7 | rs541682859 ^‡^ | 138591620 | T | C | 1.000 | -1.4050 | 0.2370 | 3.07e-09 | NA | NA |
| 8 | rs1879957 | 8544808 | T | C | 0.521 | 0.0383 | 0.0068 | 1.76e-08 | 3.70e-09 | 2.60e-02 |
| 8 | rs10101292 ^‡^ | 10856474 | T | C | 0.572 | 0.0406 | 0.0069 | 3.49e-09 | 1.60e-07 | 1.50e-05 |
| 8 | rs547244654 ^‡^ | 37341987 | T | C | 1.000 | -1.6828 | 0.2991 | 1.85e-08 | NA | NA |
| 8 | rs190344992 ^‡^ | 63934378 | G | A | 1.000 | -1.9618 | 0.3459 | 1.41e-08 | NA | NA |
| 8 | 8:83459367 ^‡^ | 83459367 | GTTTC | G | 1.000 | -1.9698 | 0.2872 | 6.96e-12 | NA | NA |
| 9 | rs561147080 ^‡^ | 15400432 | G | C | 1.000 | -1.5819 | 0.2628 | 1.76e-09 | NA | NA |
| 9 | 9:23474196 ^‡^ | 23474196 | CACA | C | 1.000 | -2.7180 | 0.4869 | 2.38e-08 | NA | NA |
| 9 | rs577213322 ^‡^ | 120405271 | G | GA | 1.000 | -2.3450 | 0.3675 | 1.76e-10 | NA | NA |
| 9 | rs1411350 | 128670179 | A | G | 0.327 | 0.0451 | 0.0072 | 4.11e-10 | 1.00e-04 | 2.80e-12 |
| 10 | rs34646324 | 22255363 | A | AT | 0.288 | -0.0464 | 0.0076 | 9.13e-10 | 9.70e-06 | 6.50e-16 |
| 10 | rs189761289 ^‡^ | 45193614 | G | A | 1.000 | -2.2087 | 0.3168 | 3.13e-12 | NA | NA |
| 11 | rs73446263 ^‡^ | 37271445 | T | C | 1.000 | -1.2704 | 0.2288 | 2.81e-08 | NA | NA |
| 11 | rs149255293 ^‡^ | 106270546 | G | A | 1.000 | -1.5307 | 0.2610 | 4.50e-09 | NA | NA |
| 11 | rs567235405 ^‡^ | 132903412 | A | C | 1.000 | -2.4449 | 0.4323 | 1.55e-08 | NA | NA |
| 12 | rs12821585 | 109886361 | T | C | 0.896 | 0.0665 | 0.0111 | 2.21e-09 | 5.10e-07 | 1.80e-08 |
| 13 | rs142801749 | 59469245 | A | AAAG | 0.687 | 0.0436 | 0.0073 | 2.64e-09 | 2.20e-08 | 1.00e-09 |
| 15 | rs567612175 ^‡^ | 35780145 | A | T | 1.000 | -1.8703 | 0.2771 | 1.49e-11 | NA | NA |
| 15 | rs181495534 ^‡^ | 54039101 | A | T | 1.000 | -3.3383 | 0.4483 | 9.60e-14 | NA | NA |
| 15 | rs144175081 ^‡^ | 69808671 | G | A | 1.000 | -1.0613 | 0.1945 | 4.89e-08 | NA | NA |
| 16 | rs112653268 ^‡^ | 68115810 | A | G | 1.000 | -2.8695 | 0.4046 | 1.32e-12 | NA | NA |
| 17 | rs543035465 ^‡^ | 10160118 | T | C | 1.000 | -1.5904 | 0.2872 | 3.06e-08 | NA | NA |
| 18 | rs376756052 ^‡^ | 12226371 | C | T | 1.000 | -2.6781 | 0.4739 | 1.59e-08 | NA | NA |
| 18 | rs145430506 ^‡^ | 63572933 | T | C | 1.000 | -2.1350 | 0.3662 | 5.54e-09 | NA | NA |
| 18 | rs111538418 ^‡^ | 77498088 | T | C | 1.000 | -2.3224 | 0.3793 | 9.21e-10 | NA | NA |
| 21 | rs574416335 ^‡^ | 33908541 | C | T | 1.000 | -0.9782 | 0.1760 | 2.71e-08 | NA | NA |
| 22 | rs1023469 ^‡^ | 42759341 | T | C | 0.607 | 0.0381 | 0.0070 | 4.63e-08 | 6.50e-07 | 2.10e-02 |
| 22 | rs572729180 ^‡^ | 46670726 | T | G | 1.000 | -1.9621 | 0.3413 | 9.00e-09 | NA | NA |

Chr, chromosome; RAF, reference allele frequency; SE, standard error; NA, not available.

^*^ P-values were calculated in the present study using FastGWA tool for linear mixed model framework.

^†^ P-values were obtained from summary statistics of more than 11 million single nucleotide polymorphisms for tablespoons of cooked and raw vegetables in previous study. Summary statistic data can be downloaded at <https://personal.broadinstitute.org/ryank/Cole_UKB_Diet_GWAS.tar>. Source: Cole JB, Florez JC, Hirschhorn JN. Comprehensive genomic analysis of dietary habits in UK Biobank identifies hundreds of genetic associations. Nat Commun. 2020;11(1):1467.

^‡^ Loci were either not available in Cole et al.’s study or not reach significant level of 5e-08.

**Summary statistics of genomic risk loci identified from genome-wide association analysis of coffee consumption**

| **Chr** | **Variant** | **Position** | **Reference allele** | **Alternative allele** | **RAF** | **Beta** | **SE** | **P_FastGWA_^*^** | **P_coffee_^†^** |
| --- | --- | --- | --- | --- | --- | --- | --- | --- | --- |
| 1 | rs10305752 ^‡^ | 150783239 | A | AG | 0.977 | 0.0827 | 0.0147 | 1.84e-08 | 1.30e-06 |
| 1 | rs574367 | 177873210 | G | T | 0.788 | -0.0305 | 0.0054 | 1.35e-08 | 3.30e-10 |
| 2 | rs71415991 | 630089 | A | ATCTAT  AATCTA  TCTCTA  TG | 0.169 | -0.0509 | 0.0059 | 5.50e-18 | 1.20e-19 |
| 2 | 2:27748992 ^‡^ | 27748992 | AT | A | 0.381 | -0.0396 | 0.0045 | 2.75e-18 | NA |
| 2 | rs12989746 ^‡^ | 49368391 | G | T | 0.750 | -0.0282 | 0.0051 | 2.83e-08 | 3.10e-07 |
| 3 | rs71326934 ^‡^ | 50536383 | C | G | 0.866 | 0.0377 | 0.0065 | 5.86e-09 | 6.20e-08 |
| 4 | rs1263412 ^‡^ | 2858005 | A | G | 0.693 | 0.0300 | 0.0048 | 3.58e-10 | 4.70e-06 |
| 4 | rs140590745 ^‡^ | 23930409 | A | C | 0.907 | 0.0435 | 0.0078 | 2.05e-08 | 1.40e-06 |
| 4 | rs2199936 | 89045331 | A | G | 0.114 | -0.0528 | 0.0069 | 2.05e-14 | 1.10e-14 |
| 5 | rs12519880 | 7391434 | C | A | 0.716 | 0.0342 | 0.0049 | 2.12e-12 | 4.40e-10 |
| 5 | rs304132 | 88215594 | A | G | 0.427 | 0.0258 | 0.0044 | 6.69e-09 | 2.80e-09 |
| 6 | rs2465037 | 51179260 | C | A | 0.657 | 0.0318 | 0.0046 | 7.68e-12 | 2.90e-13 |
| 6 | rs139797380 | 137244957 | C | G | 0.992 | 0.1521 | 0.0256 | 2.78e-09 | 4.40e-08 |
| 7 | rs4410790 | 17284577 | T | C | 0.366 | -0.1218 | 0.0046 | 8.90e-157 | 1.10e-150 |
| 7 | rs2067150 | 70051412 | A | AGTATA  ATTATG  ATTAG | 0.830 | 0.0331 | 0.0059 | 2.42e-08 | 5.40e-09 |
| 7 | rs34060476 | 73037956 | A | G | 0.866 | -0.0611 | 0.0065 | 2.67e-21 | 1.60e-23 |
| 7 | rs1057868 | 75615006 | C | T | 0.715 | -0.0619 | 0.0049 | 3.27e-37 | 5.10e-36 |
| 8 | rs13271359 | 109114426 | C | T | 0.740 | 0.0283 | 0.0050 | 1.94e-08 | 1.40e-09 |
| 9 | rs1014307 ^‡^ | 16053793 | C | T | 0.542 | 0.0254 | 0.0045 | 1.45e-08 | 4.80e-07 |
| 11 | rs597045 | 56272114 | A | T | 0.695 | 0.0264 | 0.0048 | 4.45e-08 | 1.40e-08 |
| 11 | rs181359370 ^‡^ | 84774265 | T | C | 0.999 | -0.4155 | 0.0701 | 3.07e-09 | NA |
| 12 | 12:11271915 ^‡^ | 11271915 | CA | C | 0.160 | 0.0422 | 0.0061 | 4.89e-12 | NA |
| 12 | rs11060255 | 122677724 | G | A | 0.470 | -0.0243 | 0.0044 | 4.13e-08 | 3.00e-08 |
| 15 | rs2017998 | 74721905 | G | C | 0.379 | -0.0662 | 0.0045 | 3.57e-48 | 1.20e-46 |
| 15 | rs2682909 ^‡^ | 77880927 | G | C | 0.638 | 0.0254 | 0.0046 | 3.05e-08 | 3.70e-05 |
| 15 | rs2521501 | 91437388 | A | T | 0.677 | 0.0296 | 0.0047 | 3.96e-10 | 1.80e-09 |
| 16 | rs28562191 | 53799303 | C | T | 0.580 | -0.0356 | 0.0045 | 1.47e-15 | 3.60e-23 |
| 17 | rs2905855 | 46081915 | G | A | 0.563 | 0.0270 | 0.0044 | 1.24e-09 | 7.80e-09 |
| 17 | rs57918684 ^‡^ | 60150383 | G | A | 0.846 | -0.0349 | 0.0061 | 1.07e-08 | 1.50e-07 |
| 18 | rs476828 | 57852587 | T | C | 0.762 | -0.0482 | 0.0052 | 8.93e-21 | 2.00e-23 |
| 19 | rs12459249 | 41339896 | T | C | 0.333 | -0.0346 | 0.0047 | 1.12e-13 | 2.00e-13 |
| 20 | rs6062362 | 62911229 | A | G | 0.562 | -0.0336 | 0.0045 | 4.39e-14 | 6.80e-12 |
| 22 | rs181251778 | 24901968 | A | G | 0.986 | 0.1258 | 0.0188 | 2.14e-11 | 4.50e-10 |

Chr, chromosome; RAF, reference allele frequency; SE, standard error; NA, not available.

^*^ P-values were calculated in the present study using FastGWA tool for linear mixed model framework.

^†^ P-values were obtained from summary statistics of more than 11 million single nucleotide polymorphisms for cups of coffee consumption in previous study. Summary statistic data can be downloaded at <https://personal.broadinstitute.org/ryank/Cole_UKB_Diet_GWAS.tar>. Source: Cole JB, Florez JC, Hirschhorn JN. Comprehensive genomic analysis of dietary habits in UK Biobank identifies hundreds of genetic associations. Nat Commun. 2020;11(1):1467.

^‡^ Loci were either not available in Cole et al.’s study or not reach significant level of 5e-08.

**Summary statistics of genomic risk loci identified from genome-wide association analysis of tea consumption**

| **Chr** | **Variant** | **Position** | **Reference allele** | **Alternative allele** | **RAF** | **Beta** | **SE** | **P_FastGWA_^*^** | **P_tea_^†^** |
| --- | --- | --- | --- | --- | --- | --- | --- | --- | --- |
| 1 | rs201406724 | 150781915 | T | TA | 0.652 | -0.0391 | 0.0060 | 5.48e-11 | 3.10e-10 |
| 1 | 1:154608308 ^‡^ | 154608308 | TGACTA  AATTCT  GTCTGT  CCTTTG | T | 0.775 | -0.0373 | 0.0068 | 3.89e-08 | NA |
| 1 | rs146180628 | 174413325 | G | GT | 0.859 | 0.0640 | 0.0082 | 7.81e-15 | 9.70e-14 |
| 1 | rs2813703 ^‡^ | 216913261 | G | A | 0.428 | -0.0326 | 0.0057 | 1.09e-08 | 2.00e-06 |
| 2 | rs114278367 ^‡^ | 47958631 | A | G | 0.975 | 0.1037 | 0.0182 | 1.30e-08 | 1.70e-06 |
| 2 | rs725452 | 58511300 | A | G | 0.790 | 0.0389 | 0.0069 | 2.12e-08 | 9.70e-09 |
| 3 | 3:42244986 ^‡^ | 42244986 | CTTT | C | 0.263 | -0.0353 | 0.0064 | 4.47e-08 | NA |
| 3 | rs6771054 | 89489529 | T | C | 0.595 | -0.0355 | 0.0058 | 7.68e-10 | 2.60e-11 |
| 4 | rs28863537 ^‡^ | 67932876 | T | A | 0.600 | -0.0345 | 0.0058 | 2.30e-09 | 9.90e-08 |
| 4 | rs2199936 | 89045331 | A | G | 0.114 | -0.0702 | 0.0089 | 2.36e-15 | 3.70e-15 |
| 5 | rs76823566 | 60413507 | T | TA | 0.571 | -0.0320 | 0.0057 | 2.01e-08 | 3.20e-08 |
| 5 | rs192084998 | 152077481 | G | A | 0.704 | 0.0407 | 0.0062 | 5.32e-11 | 7.40e-13 |
| 6 | rs2465018 | 51241140 | G | A | 0.769 | -0.0553 | 0.0067 | 2.05e-16 | 1.00e-17 |
| 6 | rs139797380 | 137244957 | C | G | 0.992 | 0.2337 | 0.0329 | 1.13e-12 | 1.20e-10 |
| 7 | rs1010123 ^‡^ | 13318566 | T | A | 0.768 | -0.0410 | 0.0067 | 9.07e-10 | 1.20e-07 |
| 7 | rs4410790 | 17284577 | T | C | 0.366 | -0.1150 | 0.0059 | 9.38e-86 | 2.20e-69 |
| 7 | rs6462899 | 39296489 | T | A | 0.376 | -0.0324 | 0.0058 | 2.87e-08 | 9.70e-09 |
| 7 | rs17685 | 75616105 | G | A | 0.722 | -0.0665 | 0.0063 | 3.96e-26 | 1.30e-20 |
| 7 | rs4726481 | 141668403 | G | T | 0.602 | -0.0369 | 0.0058 | 1.72e-10 | 6.70e-11 |
| 8 | rs7012814 ^‡^ | 9173358 | G | A | 0.525 | 0.0323 | 0.0057 | 1.29e-08 | 6.50e-08 |
| 8 | rs80318442 ^‡^ | 34285545 | T | G | 0.951 | -0.0749 | 0.0133 | 1.92e-08 | 5.20e-07 |
| 8 | 8:73433232 ^‡^ | 73433232 | GGTA | G | 0.299 | -0.0341 | 0.0062 | 3.35e-08 | NA |
| 9 | rs7852678 | 7031134 | A | G | 0.791 | -0.0394 | 0.0070 | 1.88e-08 | 2.60e-08 |
| 10 | rs10752269 | 12692902 | G | A | 0.493 | 0.0331 | 0.0056 | 4.40e-09 | 4.40e-09 |
| 10 | rs10764990 | 129152608 | G | A | 0.395 | 0.0321 | 0.0058 | 2.56e-08 | 9.90e-10 |
| 11 | rs11022752 | 13307622 | A | G | 0.731 | -0.0373 | 0.0064 | 4.94e-09 | 7.20e-10 |
| 11 | rs10741694 | 16286183 | T | C | 0.373 | -0.0386 | 0.0058 | 3.68e-11 | 7.70e-13 |
| 12 | 12:11271915 ^‡^ | 11271915 | CA | C | 0.160 | -0.0574 | 0.0078 | 2.61e-13 | NA |
| 12 | rs11065898 | 111862575 | C | T | 0.775 | -0.0404 | 0.0068 | 2.75e-09 | 3.30e-08 |
| 13 | rs7321386 ^‡^ | 89213194 | T | C | 0.424 | -0.0334 | 0.0057 | 4.54e-09 | 2.00e-07 |
| 13 | rs753207 ^‡^ | 111528111 | T | C | 0.569 | -0.0315 | 0.0057 | 3.73e-08 | 1.10e-05 |
| 15 | rs12591786 | 60902512 | C | T | 0.842 | 0.0488 | 0.0078 | 5.04e-10 | 5.80e-11 |
| 15 | rs12909335 | 75214789 | T | G | 0.439 | -0.0756 | 0.0057 | 2.31e-40 | 1.50e-32 |
| 16 | rs9937521 | 53799296 | C | T | 0.579 | 0.0383 | 0.0057 | 2.14e-11 | 2.00e-10 |
| 16 | rs512404 | 63031551 | G | T | 0.775 | -0.0375 | 0.0068 | 3.77e-08 | 3.40e-08 |
| 19 | rs2074550 | 19387743 | A | C | 0.664 | -0.0415 | 0.0060 | 3.61e-12 | 4.60e-11 |
| 20 | rs2273447 | 62900120 | A | T | 0.795 | -0.0511 | 0.0070 | 2.86e-13 | 2.10e-10 |
| 21 | rs4817506 ^‡^ | 34348569 | G | A | 0.499 | -0.0321 | 0.0057 | 1.51e-08 | 8.50e-07 |
| 22 | rs4820593 | 24887087 | A | T | 0.415 | -0.0665 | 0.0057 | 5.28e-31 | 1.80e-32 |
| 22 | rs73424602 | 41461176 | C | T | 0.598 | 0.0413 | 0.0058 | 6.81e-13 | 6.50e-10 |

Chr, chromosome; RAF, reference allele frequency; SE, standard error; NA, not available.

^*^ P-values were calculated in the present study using FastGWA tool for linear mixed model framework.

^†^ P-values were obtained from summary statistics of more than 11 million single nucleotide polymorphisms for cups of tea consumption in previous study. Summary statistic data can be downloaded at <https://personal.broadinstitute.org/ryank/Cole_UKB_Diet_GWAS.tar>. Source: Cole JB, Florez JC, Hirschhorn JN. Comprehensive genomic analysis of dietary habits in UK Biobank identifies hundreds of genetic associations. Nat Commun. 2020;11(1):1467.

^‡^ Loci were either not available in Cole et al.’s study or not reach significant level of 5e-08.

**Summary statistics of genomic risk loci identified from genome-wide association analysis of alcohol consumption**

| **Chr** | **Variant** | **Position** | **Reference allele** | **Alternative allele** | **RAF** | **Beta** | **SE** | **P_FastGWA_^*^** | **P_alcohol_^†^** |
| --- | --- | --- | --- | --- | --- | --- | --- | --- | --- |
| 1 | rs780569 | 4569436 | T | A | 0.813 | 0.0343 | 0.0060 | 1.31E-08 | 1.20E-09 |
| 1 | rs6678286 ^‡^ | 174169619 | A | G | 0.956 | 0.0333 | 0.0061 | 4.08E-08 | 3.90E-06 |
| 2 | rs66523860 | 23880823 | A | G | 0.878 | 0.0388 | 0.0067 | 6.48E-09 | 8.80E-10 |
| 2 | 2:27748992 ^‡^ | 27748992 | AT | A | 0.089 | -0.0882 | 0.0056 | 1.36E-55 | NA |
| 2 | rs494904 | 45141180 | T | C | 0.496 | -0.0376 | 0.0056 | 1.69E-11 | 1.70E-11 |
| 2 | rs13390019 | 97797680 | T | C | 0.387 | 0.0475 | 0.0081 | 5.14E-09 | 8.30E-10 |
| 2 | rs6436555 ^‡^ | 157488277 | A | C | 0.009 | 0.0302 | 0.0054 | 2.97E-08 | 8.30E-06 |
| 2 | 2:161338926 ^‡^ | 161338926 | TAAATA  AAC | T | 0.731 | 0.0351 | 0.0061 | 1.12E-08 | NA |
| 2 | rs5836601 | 178155252 | C | CTTT | 0.094 | -0.0385 | 0.0066 | 6.42E-09 | 2.00E-08 |
| 2 | rs10188314 | 215402926 | C | T | 0.852 | -0.0317 | 0.0054 | 5.79E-09 | 1.80E-08 |
| 3 | rs2051214 ^‡^ | 38560303 | C | A | 0.532 | 0.0321 | 0.0056 | 8.17E-09 | 3.10E-07 |
| 3 | 3:49959570 ^‡^ | 49959570 | CA | C | 0.002 | 0.0378 | 0.0055 | 6.18E-12 | NA |
| 3 | rs142623271 ^‡^ | 70676591 | A | ATGTTT | 0.606 | 0.0347 | 0.0060 | 6.85E-09 | 3.20E-05 |
| 3 | rs704255 | 71669656 | G | A | 0.580 | -0.0331 | 0.0055 | 2.00E-09 | 4.00E-08 |
| 3 | rs9822731 | 85405501 | T | C | 0.319 | -0.0404 | 0.0065 | 5.35E-10 | 6.30E-11 |
| 4 | rs2858088 | 3268710 | A | G | 0.561 | -0.0369 | 0.0056 | 4.24E-11 | 1.60E-10 |
| 4 | rs11940694 | 39414993 | A | G | 0.506 | -0.0766 | 0.0056 | 1.43E-42 | 5.50E-51 |
| 4 | rs4864919 | 55579484 | T | A | 0.650 | -0.0370 | 0.0055 | 1.75E-11 | 8.50E-09 |
| 4 | rs759387670 | 67965761 | AGATT | A | 0.611 | -0.0351 | 0.0059 | 2.37E-09 | 2.10E-08 |
| 4 | rs29001570 | 99994405 | T | C | 0.664 | 0.5175 | 0.0358 | 1.85E-47 | 2.50E-53 |
| 4 | rs13135092 | 103198082 | A | G | 0.716 | 0.0788 | 0.0099 | 1.78E-15 | 1.60E-19 |
| 5 | rs4916723 | 87854395 | A | C | 0.124 | 0.0391 | 0.0056 | 1.90E-12 | 8.50E-11 |
| 5 | rs12521723 | 132273808 | A | T | 0.093 | 0.0420 | 0.0074 | 1.10E-08 | 4.30E-09 |
| 5 | rs141688667 ^‡^ | 145660413 | T | TTTTA | 0.096 | -0.0337 | 0.0061 | 3.78E-08 | 9.00E-08 |
| 6 | rs9482094 | 98364895 | A | G | 0.250 | 0.0514 | 0.0056 | 6.39E-20 | 1.40E-13 |
| 7 | rs12701714 ^‡^ | 39326147 | G | A | 0.922 | 0.0307 | 0.0054 | 1.65E-08 | 7.90E-07 |
| 7 | rs62466318 | 73042085 | C | T | 0.456 | -0.0495 | 0.0068 | 2.25E-13 | 3.00E-13 |
| 7 | rs4726481 | 141668403 | G | T | 0.885 | 0.0331 | 0.0056 | 2.91E-09 | 2.10E-11 |
| 8 | rs1566085 | 142624527 | G | T | 0.901 | -0.0362 | 0.0055 | 4.71E-11 | 4.20E-12 |
| 8 | 8:143485680 ^‡^ | 143485680 | CTG | C | 0.099 | 0.0405 | 0.0072 | 1.81E-08 | NA |
| 11 | rs7124396 | 47787434 | T | G | 0.993 | 0.0392 | 0.0055 | 7.50E-13 | 1.30E-13 |
| 11 | rs4309187 ^‡^ | 113412443 | A | C | 0.826 | -0.0350 | 0.0059 | 2.36E-09 | 6.00E-07 |
| 11 | rs748919 | 133783232 | T | C | 0.535 | 0.0380 | 0.0067 | 1.77E-08 | 3.40E-12 |
| 12 | rs12367809 ^‡^ | 50256063 | C | T | 0.916 | 0.0312 | 0.0057 | 3.30E-08 | 2.80E-07 |
| 12 | rs12425616 | 54634666 | T | C | 0.932 | -0.0510 | 0.0073 | 2.76E-12 | 1.00E-10 |
| 12 | rs36159461 ^‡^ | 56468832 | G | GA | 0.404 | 0.0325 | 0.0057 | 1.23E-08 | 1.90E-06 |
| 13 | rs7338471 | 49981064 | G | T | 0.660 | -0.0371 | 0.0061 | 1.07E-09 | 8.20E-10 |
| 14 | rs17698314 | 56846715 | C | A | 0.621 | -0.0363 | 0.0063 | 9.26E-09 | 1.50E-08 |
| 16 | rs2286975 ^‡^ | 11114006 | G | A | 0.103 | -0.0337 | 0.0061 | 2.75E-08 | 2.70E-07 |
| 16 | rs369028384 | 13746413 | CTT | C | 0.896 | 0.0357 | 0.0064 | 1.88E-08 | 1.10E-09 |
| 16 | 16:19994176 ^‡^ | 19994176 | CT | C | 0.681 | -0.0435 | 0.0061 | 8.91E-13 | NA |
| 16 | rs78621285 | 22903022 | A | T | 0.548 | 0.0560 | 0.0096 | 4.88E-09 | 4.50E-08 |
| 16 | rs7191618 | 28565667 | C | G | 0.260 | 0.0546 | 0.0055 | 3.49E-23 | 2.10E-26 |
| 16 | rs200720048 | 30066332 | CCT | C | 0.883 | 0.0496 | 0.0055 | 3.61E-19 | 1.00E-16 |
| 16 | rs112607901 | 31003411 | G | GAC | 0.253 | -0.0338 | 0.0060 | 1.64E-08 | 2.00E-08 |
| 16 | rs9937521 | 53799296 | C | T | 0.261 | 0.0370 | 0.0055 | 1.90E-11 | 1.60E-12 |
| 16 | rs113441031 | 69763280 | C | T | 0.349 | 0.0424 | 0.0072 | 4.52E-09 | 1.00E-11 |
| 16 | rs36182990 | 71992584 | G | A | 0.840 | -0.0419 | 0.0061 | 8.48E-12 | 3.10E-10 |
| 16 | rs1104608 | 73912588 | G | C | 0.187 | 0.0317 | 0.0055 | 1.04E-08 | 1.50E-08 |
| 17 | rs8073177 | 7440584 | T | C | 0.621 | -0.0388 | 0.0065 | 2.58E-09 | 2.40E-08 |
| 17 | rs650558 | 40721042 | C | T | 0.031 | 0.0389 | 0.0063 | 5.52E-10 | 4.80E-08 |
| 17 | 17:43971206 ^‡^ | 43971206 | CTAATT | C | 0.683 | 0.0565 | 0.0067 | 3.03E-17 | NA |
| 18 | rs1788820 | 21101944 | A | G | 0.667 | -0.0475 | 0.0057 | 7.90E-17 | 4.80E-16 |
| 18 | rs1834144 | 40744790 | C | A | 0.235 | -0.0349 | 0.0056 | 5.17E-10 | 7.50E-09 |
| 18 | rs624244 | 53183396 | G | A | 0.848 | 0.0327 | 0.0058 | 1.63E-08 | 1.80E-10 |
| 18 | rs6567160 | 57829135 | T | C | 0.274 | 0.0426 | 0.0064 | 2.90E-11 | 2.10E-11 |
| 19 | rs8103840 | 49254955 | C | T | 0.046 | 0.0321 | 0.0055 | 4.93E-09 | 8.60E-12 |

Chr, chromosome; RAF, reference allele frequency; SE, standard error; NA, not available.

^*^ P-values were calculated in the present study using FastGWA tool for linear mixed model framework.

^†^ P-values were obtained from summary statistics of more than 11 million single nucleotide polymorphisms for cups of tea consumption in previous study. Summary statistic data can be downloaded at <https://personal.broadinstitute.org/ryank/Cole_UKB_Diet_GWAS.tar>. Source: Cole JB, Florez JC, Hirschhorn JN. Comprehensive genomic analysis of dietary habits in UK Biobank identifies hundreds of genetic associations. Nat Commun. 2020;11(1):1467.

^‡^ Loci were either not available in Cole et al.’s study or not reach significant level of 5e-08.

**Summary of genome-wide association findings for selected food items in Cole et al.’s study**

| **Food item** | **No. significant variants (p<5e-8)** | **No. shared significant variants** | **Heritability (%)** |
| --- | --- | --- | --- |
| Beef | 1,528 | 497 | 7.6 |
| Pork | 802 |  | 5.1 |
| Lamb/ mutton | 2,663 |  | 4.9 |
| Processed meat | 1,461 | - | 6.6 |
| Poultry | 172 | - | 4.9 |
| Oily fish | 10,340 | 313 | 8.9 |
| Non-oily fish | 456 |  | 4.6 |
| Milk  Full cream vs. never  Semi-skimmed vs. never  Skimmed vs. never | 1  77  88 | 0 | 24.3  4.2  9.8 |
| Cheese | 3,053 | - | 10.8 |
| Fresh fruit | 9,312 | 694 | 10.8 |
| Dried fruit | 1,807 |  | 7.5 |
| Cooked vegetable | 2,334 | 242 | 7.3 |
| Raw vegetable | 4,388 |  | 7.3 |
| Coffee | 5,121 | - | 7.9 |
| Tea | 5,853 | - | 9.1 |
| Alcohol | 10,489 | - | 12.1 |

Summary statistics of more than 11 million single nucleotide polymorphisms are obtained at <https://personal.broadinstitute.org/ryank/Cole_UKB_Diet_GWAS.tar>

Source: Cole JB, Florez JC, Hirschhorn JN. Comprehensive genomic analysis of dietary habits in UK Biobank identifies hundreds of genetic associations. Nat Commun. 2020;11(1):1467.

**Number of significant variants for food intake comparing with previous study**

| **Current study** |  | **Cole et al.’s study ^*^** |  |  |  |
| --- | --- | --- | --- | --- | --- |
| **Food item** | **Genomic risk loci** | **Food item** | **Not available** | **Suggestive significant SNPs (5e-08≤ p<1e-05)** | **Non-significant SNPs (p≥1e-5)** |
| Red meat | 15 | Beef, pork, lamb | 3 | 2 | 4 |
| Processed meat | 12 | Processed meat | 0 | 7 | 0 |
| Poultry | 1 | Poultry | 0 | 0 | 1 |
| Total fish | 28 | Oily fish, non-oily fish | 4 | 7 | 1 |
| Milk | 50 | Milk type | 9 | 0 | 41 |
| Cheese | 59 | Cheese | 9 | 25 | 3 |
| Total fruits | 82 | Fresh fruits, dried fruits | 35 | 5 | 1 |
| Total vegetables | 50 | Cooked vegetables, raw vegetables | 39 | 3 | 0 |
| Coffee | 33 | Coffee | 3 | 7 | 1 |
| Tea | 40 | Tea | 4 | 8 | 1 |
| Alcohol | 57 | Alcohol | 6 | 9 | 1 |
| Total | 399 |  | 107 | 73 | 54 |

^*^ Source: Cole JB, Florez JC, Hirschhorn JN. Comprehensive genomic analysis of dietary habits in UK Biobank identifies hundreds of genetic associations. Nat Commun. 2020;11(1):1467.
